# Synthesis of glycoconjugate fragments of mycobacterial phosphatidylinositol mannosides and lipomannan

**DOI:** 10.3762/bjoc.7.47

**Published:** 2011-03-28

**Authors:** Benjamin Cao, Jonathan M White, Spencer J Williams

**Affiliations:** 1 School of Chemistry and Bio21 Molecular Science and Biotechnology Institute, University of Melbourne, Parkville, Victoria, Australia, Fax: +61 3 9347 8124; Tel: +61 3 8344 2422

**Keywords:** fluorescently-labelled sugars, glycoconjugates, lipomannan, mycobacteria, tuberculosis

## Abstract

*Mycobacterium tuberculosis*, the causitive agent of tuberculosis (TB), possesses a complex cell wall containing mannose-rich glycophospholids termed phosphatidylinositol mannosides (PIMs), lipomannan (LM), and lipoarabinomannan (LAM). These glycophospholipids play important roles in cell wall function and host–pathogen interactions. Synthetic PIM/LM/LAM substructures are useful biochemical tools to delineate and dissect the fine details of mannose glycophospholipid biosynthesis and their interactions with host cells. We report the efficient synthesis of a series of azidooctyl di- and trimannosides possessing the following glycan structures: α-Man-1,6-α-Man, α-Man-1,6-α-Man-1,6-α-Man, α-Man-1,2-α-Man-1,6-α-Man and 2,6-di-(α-Man)-α-Man. The synthesis includes the use of non-benzyl protecting groups compatible with the azido group and preparation of the branched trisaccharide structure 2,6-di-(α-Man)-α-Man through a double glycosylation of a 3,4-butanediacetal-protected mannoside. The azidooctyl groups of these synthetic mannans were elaborated to fluorescent glycoconjugates and squaric ester derivatives useful for further conjugation studies.

## Introduction

The incidence of TB is now at an all-time historical high with over 2 billion people infected globally [1]. TB is the leading infectious killer of people with HIV/AIDS and is second only to HIV/AIDS as an infectious cause of death for adults [2]. It is sobering that it has been more than 40 years since the last frontline TB drug (rifampicin) was deployed [3]. Drug resistance is now widespread and growing, underscoring the need for the development of new therapies to bolster the physician's armamentarium for TB control [3]. Many existing TB drugs target aspects of mycobacterial cell wall biosynthesis (e.g., thiacetazone, isoniazid, ethambutol, pyrazinamide, and ethionamide) with the cell wall of the tubercule bacillus being widely agreed as a promising target for new drugs [4–5]. The cell wall of all mycobacteria is especially rich in lipids and polysaccharides, with the major component being a macromolecule composed of mycolic acids, arabinogalactan, and peptidoglycan, termed the mycolyl–arabinogalactan–peptidoglycan complex [6–7]. One intriguing class of cell wall associated molecules are those based on a phosphatidylinositol (PI) core, which include the PIMs, LM, and LAM [8].

**Scheme 1:**
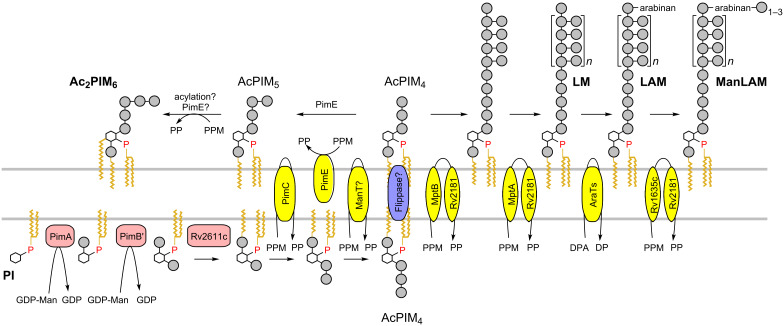
Indicative topology model for the biosynthesis of the glycophospholipids PIMs, LM and LAM in mycobacteria. The timing for translocation of PIM intermediates across the membrane is unclear. Hexagon = *myo*-inositol; closed circle = mannose; P = phosphate.

Through studies with gene deletion mutants of mycobacterial strains, several steps in the biosynthesis of the PIMs, LM and LAM have been shown to be essential for bacterial survival and it is now well appreciated that they are crucial cell-surface molecules that mediate host–pathogen interactions [8–9]. Biochemical studies support the general biosynthetic relationship PIMs → LM → LAM, although it is also clear that Ac_2_PIM_2_ and Ac_2_PIM_6_ represent important metabolic end products in their own right [10]. Scheme 1 summarizes the biosynthesis of the mannan core of the PIMs, LM and LAM. PIM biosynthesis commences with the stepwise transfer of two mannosyl residues onto inositol, catalyzed by the GDP-mannose dependent α-mannosyltransferases PimA [11] and PimB' [12–13], followed by acylation by the acyltransferase (Rv2611c) to give AcPIM_2_ [14–15]. Additional α-1,6-mannosylations of AcPIM_2_ give rise to AcPIM_3_ and AcPIM_4_, the last of which is hypothesized to be a key biosynthetic precursor for the synthesis of the so-called polar PIMs, AcPIM_5_ and Ac_2_PIM_6_, and LM and LAM [16].

The biosynthesis of LM and LAM (Scheme 1) commences from AcPIM_4_ with the installation of a linear α-1,6-linked mannan backbone on the terminal mannose [17]. Two α-1,6-mannosyltransferases, MptB and MptA, have been identified to be involved in the elongation of the LM backbone [18–19]. The linear backbone is then elaborated with single α-1,2-linked mannose residues to give mature LM [20]. LAM is formed by addition of arabinan to the penultimate mannose residue of LM, and is subsequently capped with a variety of groups including inositol phosphate, 5-methylthioxylose and its sulfoxide, and short 1,2-mannose oligomers [7].

Studies into the biosynthesis of the PIMs, LM and LAM have been greatly facilitated by the development of glycomimetic compounds. Homogeneous synthetic substructures have been used to deconvolute aspects of substrate recognition by biosynthetic enzymes and the structural determinants of host–pathogen interactions including antibody recognition and immune pattern-recognition systems such as the dendritic cell specific intercellular adhesion molecule-grabbing non-integrin (DC-SIGN) [8]. Thus, while total syntheses of many PIM structures have now been reported, the synthesis of substructures remains a worthwhile endeavor as these are useful to clarify fine details of enzymatic substrate recognition and are substantially easier to prepare [4,8]. As a shining example, synthetic octyl α-1,6-linked oligomannoside analogues of the 1,6-mannan core are effective substrates for mycobacterial cell free systems [21–23], and were used to confirm the activity of the polymerizing α-1,6-mannosyltransferases MptB and MptA [18–19], and to demonstrate functional compartmentalization of PPM synthase activity and MptB/MptA [24]. They have also been used as glycolipid substrates supporting the development of inhibitors of PIM/LM/LAM biosynthesis [25–28]. Various 1,2-linked aminooctyl oligomannosides corresponding to the capping groups of ManLAM were prepared and conjugated to carrier proteins and used to study antibody reactivity in a serological TB assay [29–30]. A complete set of the phosphoglycan head groups of PIM_1_–PIM_6_ with a thiol linker in place of the diacylglycerol were prepared and, following immobilization on glass slides, their binding to the lectin DC-SIGN was assessed [31].

**Figure 1:**
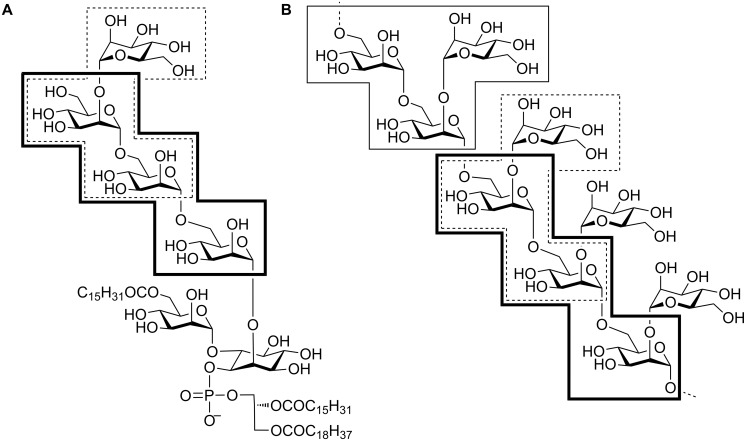
Chemical structures of (A) a representative PIM, AcPIM_5_ and (B) a mannan fragment of LM from mycobacteria. Boxes denote the relationship of PIMs and LM to trisaccharide fragments synthesized in this study.

**Figure 2:**
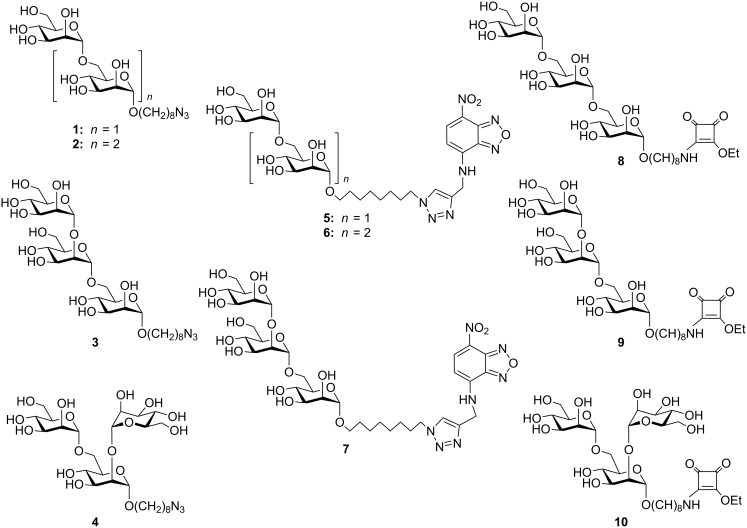
Target di- and trisaccharide glycoconjugate fragments of PIMs and LM.

Significant questions remain in the area of PIM/LM/LAM biosynthesis that could be assisted by suitable well-defined mannan substructures. For example, the identity of the α-1,2-mannosyltransferase(s) involved in the conversion of AcPIM_4_ → AcPIM_5_ → Ac_2_PIM_6_ remain incompletely characterized [17]. Similarly, the timing of the introduction of the single α-1,2-mannose residues onto the α-1,6-linked mannan core versus the elongation of this core is unclear [32]. For these reasons, we have undertaken the synthesis of a suite of fragments of the PIMs and LM, **1**–**4**, and report their elaboration into glycoconjugates **5**–**10** for use as biological reagents to study PIM/LM/LAM biosynthesis and immunogenicity (Figure 1 and Figure 2). The azidooctyl aglycon has particular utility in this regard because of its (i) lipophilicity allowing biphasic partitioning between butanol/water or purification by reversed-phase extraction, (ii) ability to be reduced to an aminooctyl chain for use in squarate conjugation chemistry, and (iii) capacity to be conjugated with fluorescent terminal alkynes using the Cu(I)-catalyzed azide–alkyne cycloaddition (CuAAC) reaction [33–34].

## Results and Discussion

Since their introduction by Palcic and co-workers [35], hydrophobic alkyl glycosides have proven to be valuable derivatives for enzymatic assays, as their lipophilic nature allows easy product isolation by either reversed-phase chromatography or simple solvent partitioning. Incorporation of an azidooctyl group confers many of the same benefits as an octyl aglycon, with the additional advantage that the azido group may be elaborated into glycoconjugates. However, the synthesis of azidooctyl glycosides can be challenging as the use of reductively-removed protecting groups such as benzyl ethers must be avoided owing to their incompatibility with the azido group when using H_2_/Pd or Na/NH_3_. We therefore sought to develop a synthesis based on the use of esters, silyl ethers and acetals only.

### Synthesis of monosaccharide building blocks

**Scheme 2:**
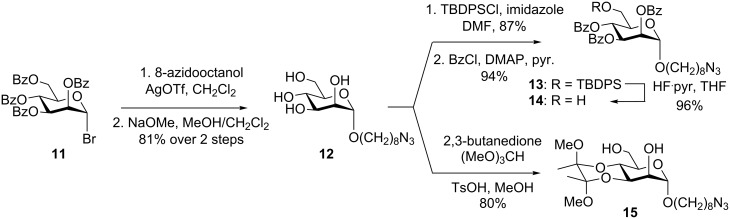
Synthesis of azidooctyl alcohol **14** and diol **15**.

Glycosidation of 8-azidooctan-1-ol (Supporting Information File 1) using glycosyl bromide **11** [36] in the presence of AgOTf, and debenzoylation of the crude product gave **12** in 81% yield over 2 steps (Scheme 2). Regioselective silylation of the primary alcohol of **12** with TPSCl followed by benzoylation of the remaining hydroxyl groups afforded the glycoside **13**, which was desilylated with HF·pyridine complex to yield **14**. This chemoselective transformation uses conditions that are similar to those reported by Tam et al. [23], and result in desilylation in a significantly shorter period than that previously reported using HCl in MeOH/Et_2_O [22]. The diol **15** was prepared by treatment of **12** with 2,3-butanedione and trimethyl orthoformate in the presence of catalytic acid in refluxing MeOH (Scheme 2) [37]. Trace amounts of the corresponding methyl glycoside were also obtained, arising from limited methanolysis of the glycosidic linkage.

**Scheme 3:**
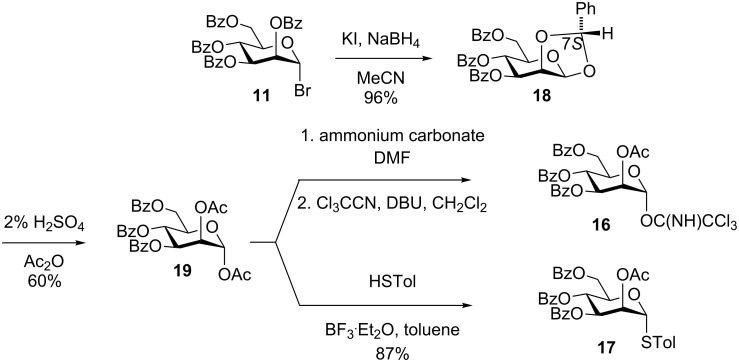
Synthesis of mannosyl donors **16** and **17**.

**Figure 3:**
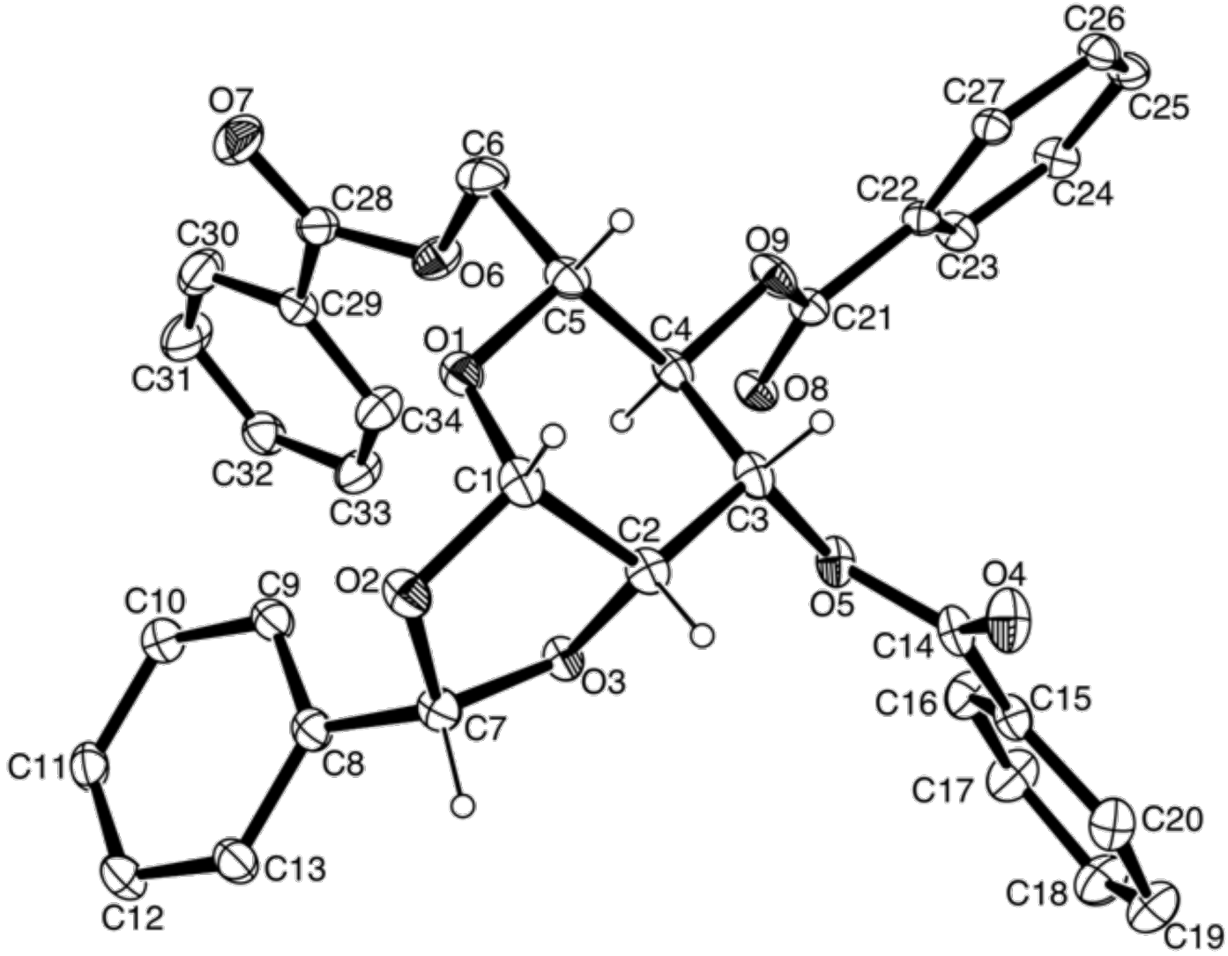
ORTEP plot of single crystal X-ray determination of (7*S*)-**18**. Thermal ellipsoids denote 20% electron probability.

For α-mannosylation of primary and secondary alcohols, the mannosyl donors **16** [38] and **17** were used. Treatment of glycosyl bromide **11** with NaBH_4_/KI in MeCN [39] afforded the crystalline 1,2-*O*-benzylidene acetal **18** as a single diastereoisomer in quantitative yield (Scheme 3). The stereochemistry of the benzylidene acetal **18** formed by this method has been studied by Suzuki et al. who assigned the product as the (7*S*)-stereoisomer (but reported it as the (7*R*)-isomer) by observation of a nuclear Overhauser effect transfer between the methine proton of the benzylidene acetal and H2 [40]. Unambiguous stereochemical assignment of (7*S*)-**18** was achieved by single crystal X-ray analysis as shown in Figure 3, and is consistent with stereoselective delivery of hydride to the *exo*-face of the intermediate dioxolenium ion. Acetolysis of the benzylidene acetal **18** using 2% H_2_SO_4_/Ac_2_O provided diacetate **19** in 60% yield. Compound **19** was converted into the trichloroacetimidate **16** following the approach of Kong and coworkers [38] or to the thioglycoside **17** by treatment with *p*-thiocresol and BF_3_·Et_2_O in toluene.

### Assembly of mannosides 1–4

**Scheme 4:**
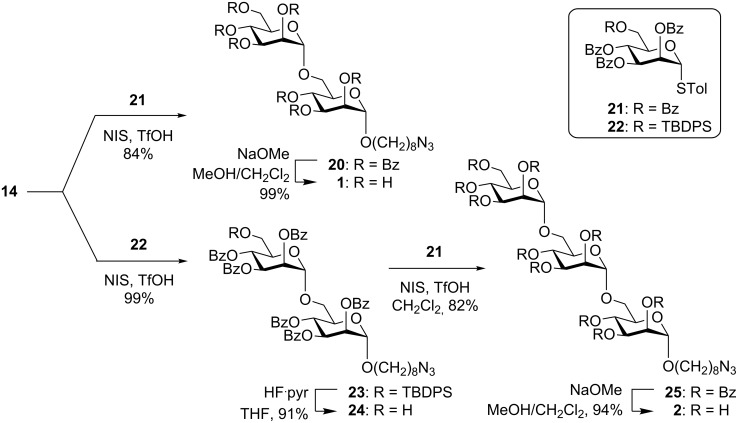
Synthesis of disaccharide **1** and trisaccharide **2**.

Synthesis of the protected disaccharide **20** was achieved by glycosylation of **14** with the thioglycoside donor **21** [41] using NIS/TfOH in 84% yield (Scheme 4). The protected trisaccharide **25** was prepared by an approach similar to that reported for the corresponding octyl trisaccharide [22–23]. Thus glycosylation of **14** with the silylated donor **22** using NIS/TfOH afforded the silylated disaccharide **23** (99%). Compound **23** was desilylated using HF·pyridine and the primary alcohol **24** was glycosylated using thioglycoside **21** to give the trimannoside **25**. Evidence for the exclusive formation of the α-anomer in all mannosylations in this work was obtained through measurement of the ^1^*J*_C,H_ coupling constants for the anomeric carbons of the newly formed products. Each coupling constant was >170 Hz, thereby showing that all new *O*-glycosidic linkages were α-configured (Supporting Information File 1) [42]. Global debenzoylation of disaccharide **20** and trisaccharide **25** with catalytic NaOMe in MeOH/CH_2_Cl_2_ provided **1** and **2** in yields of 99% and 94%, respectively.

**Scheme 5:**
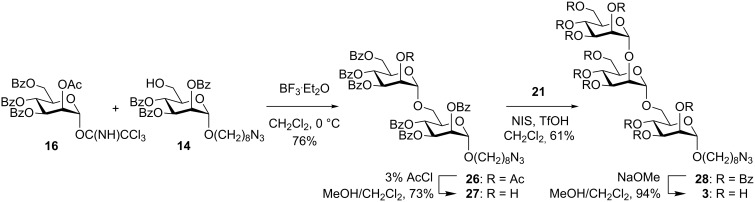
Synthesis of trisaccharide **3**.

Our strategy towards the synthesis of the trisaccharide **3** sought to utilize a glycosyl donor possessing a 2-*O*-acetyl group with benzoyl groups at the remaining positions, anticipating that selective deacetylation post-glycosylation could be achieved to allow the subsequent synthesis of the α-Man-1,2-Man linkage (Scheme 5). Activation of a mixture of alcohol **14** and trichloroacetimidate **16** at 0 °C with 0.1 equiv of BF_3_·Et_2_O provided disaccharide **26** in only 21% yield, with a 1,2-glycosyl orthoester as the major product (40%). Orthoesters are common by-products of glycosylation reactions and typically rearrange under acidic conditions to give *trans*-linked glycosides [43]. Thus, **14** and **16** were treated with 0.25 equiv of an alternative Lewis acid, TMSOTf, and allowed to react for a longer time to allow the intermediate orthoester to isomerize. Under these conditions the disaccharide **26** was isolated in an improved yield of 63%. Also isolated was a 6-*O*-trimethylsilyl ether (6%), resulting from the reaction of alcohol **14** with TMSOTf. Better still, treatment of **14** and **16** with 0.25 equiv of BF_3_·Et_2_O afforded **26** in 76% yield. A similar outcome was obtained using NIS/TfOH activation of thioglycoside donor **17** to furnish disaccharide **26** in 72% yield. Selective deacetylation of **26** was achieved by acidic transesterification using 3% AcCl in MeOH/CH_2_Cl_2_ to give the secondary alcohol **27** in 73% yield. Mannosylation of **27** using donor **21** under NIS/TfOH activation gave trisaccharide **28** in 61% yield, and global debenzoylation proceeded smoothly to give **3**.

**Scheme 6:**
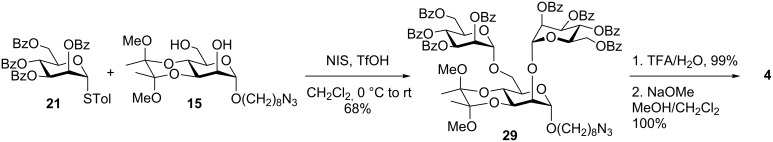
Synthesis of trisaccharide **4**.

The trisaccharide **4** was prepared by simultaneous glycosylation of the 2 and 6 positions of acceptor **15** (Scheme 6). Treatment of the diol **15** with 4 equiv of donor **21**, and NIS/TfOH afforded the protected trisaccharide **29** in 68% yield. Deprotection was achieved by sequential treatment with TFA/H_2_O and NaOMe/MeOH to afford the trisaccharide **4**.

### Synthesis of glycoconjugates 5–10

**Scheme 7:**
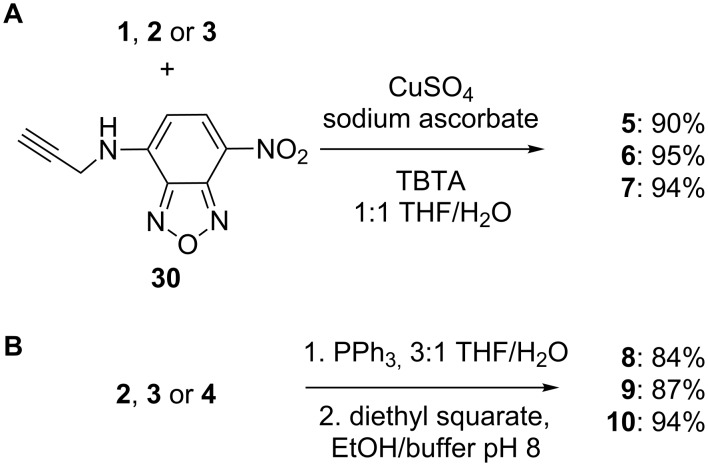
Synthesis of glycoconjugates **5**–**7** and **8**–**10**.

Nitrobenzodiazole (NBD) dyes are useful fluorescent labels owing to their small size, low cost, superior water solubility relative to other common alternatives, and ability to be excited using visible, rather than ultraviolet light [44]. Hindsgaul and coworkers have reported the use of glycoconjugates with NBD dyes to streamline the detection of carbohydrate-lectin interactions and report that the NBD group displayed substantially less nonspecific interaction with proteins over other fluorescent dyes [45]. Using alkynyl-NBD **30** (prepared in one step from NBD chloride and propargylamine) [34], the disaccharide **1** and trisaccharides **2** and **3** were coupled upon treatment with CuSO_4_, sodium ascorbate and the Cu(I)-stabilizing ligand tris(benzyltriazolylmethyl)amine (TBTA) (Scheme 7) [46–47]. The resulting dye-labelled glycoconjugates **5**–**7** were isolated in 90–95% yields and possessed excellent fluorescent properties with λ_ex_ = 400 nm and λ_em_ = 530 nm.

The squarate diester methodology introduced by Tietze and coworkers [48] and recently refined by the Kováč group [49] for the attachment of amine-derivatized carbohydrates to carrier proteins has particular advantages over other linker methodologies as diethyl squarate is commerically available and exhibits good selectivity in each coupling step, with the intermediate squaramate ester being sufficiently stable to allow its purification and storage. It should be noted that a key limitation of the methodology is the potential immunogenicity of the squarate group [50]. The three trisaccharides **2**–**4** were reduced to the aminooctyl derivatives by treatment with Ph_3_P in THF/water, and treated with diethyl squarate according to the procedure of Kováč (Scheme 7) [49]. Purification by reversed-phase chromatography afforded the ethyl squaramyl derivatives **8**–**10** in 84–94% yields.

## Conclusion

We report the synthesis of four di/trisaccharide fragments of mycobacterial PIMs/LM/LAM and their elaboration to fluorescently-labelled glycoconjugates and haptens for the preparation of antigens. A readily prepared and crystalline 1,2-*O*-benzylidene acetal **18** has been used as a central precursor for the preparation of 2-*O*-acetyl mannosyl donors **16** and **17**. The synthetic routes are compatible with the azido group.

## Supporting Information

File 1 Experimental part.

File 2 ^1^H and ^13^C NMR spectra for new compounds and fluorescence spectra for **5**–**7**.
The crystallographic data file for the structure reported in this paper has been deposited with the Cambridge Crystallographic Data Centre as file CCDC 804936 and is available on request from http://www.ccdc.cam.ac.uk/.
