# Supplementary material for: Synthesis of glycoconjugate fragments of mycobacterial phosphatidylinositol mannosides and lipomannan
Source: Beilstein J Org Chem. 2011 Mar 28;7:369–77. doi: 10.3762/bjoc.7.47 (PMC3079179; doi:10.3762/bjoc.7.47)
Supplement: File 1 — Experimental part. [file Beilstein_J_Org_Chem-07-369-s001.pdf]

Supporting Information File 1

for

Synthesis of glycoconjugate fragments of mycobacterial phosphatidylinositol mannosides and lipomannan

Benjamin Cao, Jonathan M. White and Spencer J. Williams*

Address: School of Chemistry and Bio21 Molecular Science and Biotechnology Institute, University of Melbourne, Parkville, Victoria, Australia, Fax: +61 3 9347 8124; Tel: +61 3 8344 2422

E-mail: Spencer J. Williams* - sjwill@unimelb.edu.au

* Corresponding author

Experimental part

General methods: Pyridine was distilled over KOH before use. Dichloromethane and THF were dried over alumina according to the method of Pangborn and coworkers [1]. Reactions were monitored by thin layer chromatography (TLC) performed on Merck Silica Gel 60 F_{254}. Detection was effected by charring in a mixture of 5% sulfuric acid in methanol, vanillin stain (6% vanillin, 1% H_2SO_4 in EtOH), 10% phosphomolybdic acid in EtOH, and/or visualising with UV light. Flash chromatography was performed according to the method of Still et al. [2] with Merck Silica Gel 60. Melting points were obtained using a Reichert–Jung hotstage microscope (corrected). Optical rotations were obtained using a JASCO DIP-1000 polarimeter. $[\alpha]_D$ values are given in 10^{-1} cm^2 g^{-1}. ^1H and ^{13}C NMR spectra were recorded

with an Inova 500 instrument. Elemental analyses were conducted by C.M.A.S. (Belmont, Victoria). High resolution mass spectrometry was performed on a Finnigan hybrid LTQ-FT mass spectrometer (Thermo Electron Corp.). Fluorescence data was acquired on a Thermo-Electron Varioskan multimode plate reader at an excitation wavelength of 400 nm.

Synthesis of 8-azidooctan-1-ol

A mixture of NaN_3 (1.95 g, 30.0 mmol) and 8-chlorooctan-1-ol (3.29 g, 19.9 mmol) in DMSO (5 mL) was heated to 80 °C and stirred for 18 h. The mixture was diluted with EtOAc (30 mL) and washed thoroughly with H_2O (10 × 10 mL). The organic phase was dried ($MgSO_4$) and evaporated to afford 8-azidooctan-1-ol (3.42 g), δ_H (500 MHz, $CDCl_3$) 1.19–1.63 (12H, m, $HOCH_2(CH_2)_6CH_2N_3$), 3.26 (2H, t, J = 7.0 Hz, CH_2N_3), 3.64 (2H, m, $HOCH_2$); δ_C (125 MHz, $CDCl_3$) 25.57–32.56 (6C, $HOCH_2(CH_2)_6CH_2N_3$), 51.33 (1C, CH_2N_3), 62.70 (1C, $HOCH_2$).

Azidooctyl α-D-mannopyranoside (12)

AgOTf (1.18 g, 4.57 mmol) was added to a stirred mixture of 2,3,4,6-tetra-*O*-benzoyl-α-D-mannosyl bromide **11** (2.32 g, 3.52 mmol), 8-azidooctanol (904 mg, 5.28 mmol) and freshly activated powdered 4 Å molecular sieves in dry CH_2Cl_2 (30 mL) at rt. The mixture was stirred overnight under a N_2 atmosphere and then filtered through a layer of Celite®. The filtrate was concentrated under reduced pressure to give the crude azidooctyl mannoside as a viscous oil. The residue was dissolved in a mixture of CH_2Cl_2/MeOH (2:1, 15 mL) and a solution of NaOMe/MeOH (1.5 M, 1 mL) added. The mixture was stirred at rt for 2 h and then neutralized with Amberlite 120R resin (H^+ form). The mixture was filtered and the filtrate concentrated under reduced pressure. The residue was diluted with water (10 mL) and washed with EtOAc. The organic phase was extracted with water (5 × 10 mL) and the combined aqueous phases concentrated under reduced pressure. Flash chromatography (27:2:1 EtOAc/MeOH/H_2O) of the residue afforded the tetraol **12**

(954 mg, 81%) as a pale yellow oil. $[\alpha]_D$ +49 (*c* 0.52 in MeOH); δ_H (500 MHz, d_4-methanol) 1.39–1.65 (12H, m, $OCH_2(CH_2)_6CH_2N_3$), 3.30 (2H, t, J = 7.0 Hz, CH_2N_3), 3.44 (1H, ddd, J = 6.5, 9.5, 12.5 Hz, OCH_2CH_2), 3.54 (1H, m, H5), 3.63 (1H, dd, $J_{3,4}$ = 9.5, $J_{4,5}$ = 9.5 Hz, H4), 3.70–3.78 (3H, m, H3,6a,OCH_2CH_2), 3.80 (1H, dd, $J_{1,2}$ = 1.5, $J_{2,3}$ = 3.5 Hz, H2), 3.85 (1H, dd, $J_{5,6}$ = 2.5, $J_{6,6}$ = 11.5 Hz, H6b), 4.75 (1H, d, H1); δ_C (100 MHz, d_4-methanol) 27.25, 27.77, 29.90, 30.20, 30.40, 30.56 (6C, $OCH_2(CH_2)_6CH_2N_3$), 52.45 (1C, CH_2N_3), 62.95, 68.55, 68.66, 72.30, 72.69, 74.58 (6C, C2,3,4,5,6,OCH_2CH_2), 101.55 (1C, C1); HRMS (ESI+) *m/z* 356.1792 ($C_{14}H_{27}N_3O_6$ $[M + Na]^+$ requires 356.1792).

8-Azidooctyl 2,3,4-tri-*O*-benzoyl-6-*O*-(*tert*-butyldiphenylsilyl)-α-D-mannopyranoside (13)

A mixture of *tert*-butylchlorodiphenylsilane (607 μL, 2.34 mmol), tetraol **12** (650 mg, 1.95 mmol) and imidazole (330 mg, 4.88 mmol) in DMF (2 mL) was stirred at 35 °C for 4 h, then quenched with water (3 mL) and stirred for a further 10 min before being diluted with EtOAc (50 mL). The mixture was washed sequentially with water (3 × 10 mL), aq. HCl (1 M, 10 mL) and sat. aq. $NaHCO_3$ (10 mL). The organic extract was dried ($MgSO_4$) and the solvent evaporated under reduced pressure to yield a residue, which was purified by flash chromatography (60:40 EtOAc/petroleum ether) to afford 8-azidooctyl 6-*O*-(*tert*-butyldiphenylsilyl)-α-D-mannopyranoside (970 mg, 87%) as a pale yellow syrup. $[\alpha]_D$ +19 (*c* 1.1 in $CHCl_3$), δ_H (500 MHz, $CDCl_3$) 1.07 (9H, s, *tert*-butyl), 1.31–1.61 (12H, m, $OCH_2(CH_2)_6CH_2N_3$), 2.32, 2.68, 3.00 (3H, br s, OH), 3.25 (2H, t, J = 7.0 Hz, CH_2N_3), 3.36 (1H, ddd, J = 6.5, 9.5, 13.0 Hz, OCH_2CH_2), 3.59–3.67 (2H, m, H5,OCH_2CH_2), 3.79–3.94 (5H, m, H2,3,4,6a,6b), 4.78 (1H, d, $J_{1,2}$ = 1.5 Hz, H1), 7.38–7.70 (10H, m, Ph); δ_C (125 MHz, $CDCl_3$) 19.34, 26.19, 26.80, 26.99, 28.96, 29.21, 29.39, 29.48 (8C, $OCH_2(CH_2)_6CH_2N_3$,$(CH_3)_3C$), 51.60 (1C, CH_2N_3), 65.46, 67.85, 70.65, 70.67, 70.80, 71.88 (6C, C2,3,4,5,6,OCH_2CH_2), 99.52 (1C, C1), 127.97, 130.18, 132.94, 133.00, 135.74 (10C,

Ph).

A mixture of benzoyl chloride (230 μL, 2.01 mmol), the triol (230 mg, 0.402 mmol) and DMAP (10 mg) in pyridine/CH_2Cl_2 (1:1, 4 mL) was stirred overnight. The reaction was quenched with water (2 mL), stirred for a further 10 min and then diluted with EtOAc (50 mL). The organic phase was washed successively with water (3 × 30 mL), aq. HCl (1 M, 10 mL), sat. aq. $NaHCO_3$ (30 mL) and brine (30 mL), then dried ($MgSO_4$) and concentrated under reduced pressure. Flash chromatography (10:90 EtOAc/ petroleum ether) of the residue afforded the silyl ether **13** (333 mg, 94%) as a pale yellow oil. $[\alpha]_D$ −84 (*c* 0.9 in $CHCl_3$); δ_H (500 MHz, $CDCl_3$) 1.05 (9H, s, *tert*-butyl), 1.37–1.68 (12H, m, $OCH_2(CH_2)_6CH_2N_3$), 3.26 (2H, t, J = 7.0 Hz, CH_2N_3), 3.54 (1H, ddd, J = 6.5, 9.5, 13.5 Hz, OCH_2CH_2), 3.79 (1H, ddd, J = 6.5, 9.5, 13.5 Hz, OCH_2CH_2), 3.83 (1H, dd, $J_{5,6}$ = 2.0, $J_{6,6}$ = 11.5 Hz, H6a), 3.90 (1H, dd, $J_{5,6}$ = 4.5 Hz, H6b), 4.15 (1H, m, H5), 5.14 (1H, d, $J_{1,2}$ = 1.5 Hz, H1), 5.67 (1H, dd, $J_{2,3}$ = 3.5 Hz, H2), 5.84 (1H, dd, $J_{3,4}$ = 10.0 Hz, H3), 6.09 (1H, dd, $J_{4,5}$ = 10.0 Hz, H4), 7.16–8.12 (25H, m, Ph); δ_C (125 MHz, $CDCl_3$) 19.20, 26.08, 26.63, 26.68, 28.84, 29.08, 29.30, 29.36 (10C, $OCH_2(CH_2)_6CH_2N_3$, $(CH_3)_3C$), 51.47 (1C, CH_2N_3), 62.71, 66.79, 68.26, 70.71, 70.90, 71.44 (6C, C2,3,4,5,6,OCH_2CH_2), 97.48 (1C, C1), 127.53–135.67 (25C, Ph), 165.29, 165.61, 165.64 (3C, *C*OPh); HRMS (ESI+) *m*/*z* 906.3754 ($C_{51}H_{57}N_3NaO_9Si$ $[M + Na]^+$ requires 906.3762); IR ν (cm^{-1}) 1730 sharp (C=O), 2095 sharp (N_3).

8-Azidooctyl 2,3,4-tri-*O*-benzoyl-α-D-mannopyranoside (14)

Hydrogen fluoride–pyridine complex (70%, 1.5 mL) was added to a stirred solution of the silyl ether **13** (931 mg, 1.06 mmol) in THF (12 mL) at 0 °C. The solution was allowed to warm to rt and stirred for 5 h. The mixture was diluted with EtOAc and washed with sat. aq. $NaHCO_3$ (3 × 30 mL). The organic phase was dried ($MgSO_4$) and the solvents removed under reduced pressure. Flash chromatography (20:80 EtOAc/petroleum ether) of

the residue yielded the alcohol **14** (654 mg, 96%) as a pale yellow oil. $[\alpha]_D$ –106 (c 1.2 in $CHCl_3$); (Found: C, 65.11; H, 6.10; N, 6.62. $C_{35}H_{39}N_3O_9$ requires C, 65.10; H, 6.09; N, 6.51%); δ_H (500 MHz, $CDCl_3$) 1.38–1.69 (12H, m, $OCH_2(CH_2)_6CH_2N_3$), 3.28 (2H, t, *J* = 7.0 Hz, CH_2N_3), 3.55 (1H, ddd, *J* = 6.5, 9.5, 13.0 Hz, OCH_2CH_2), 3.76–3.84 (3H, m, H6a,6b,OCH_2CH_2), 4.06 (1H, m, H5), 5.09 (1H, d, $J_{1,2}$ = 1.5 Hz, H1), 5.66 (1H, dd, $J_{2,3}$ = 3.5 Hz, H2), 5.83 (1H, dd, $J_{3,4}$ = 10.0 Hz, $J_{4,5}$ = 10.0 Hz, H4), 5.98 (1H, dd, H3), 7.24–8.11 (15H, m, Ph); δ_C (125 MHz, $CDCl_3$) 26.03, 26.67, 28.83, 29.05, 29.25, 29.32 (6C, $OCH_2(CH_2)_6CH_2N_3$), 51.46 (1C, $CH_2CH_2N_3$), 61.42, 67.40, 68.60, 69.71, 70.77, 70.89 (6C, C2,3,4,5,6,OCH_2CH_2), 97.69 (1C, C1), 128.29–133.65 (15C, Ph), 165.51, 165.58, 166.53 (3C, *C*OPh); HRMS (ESI+) *m*/*z* 668.2578 ($C_{35}H_{39}N_3NaO_9$ $[M + Na]^+$ requires 668.2579).

3,4,6-Tri-*O*-benzoyl-1,2-*O*-(*S*)-benzylidene-β-D-mannopyranose (18)

A mixture of the crude bromide **11** (4.99 g, 7.57 mmol), potassium iodide (1.91 g, 11.5 mmol) and $NaBH_4$ (0.44 g, 11.6 mmol) in acetonitrile (30 mL) was heated under reflux overnight. The mixture was extracted with chloroform (3 × 50 mL) and the combined extracts washed with water (3 × 100 mL) and brine (2 × 100 mL). The organic phase was dried ($MgSO_4$) and the solvent evaporated under reduced pressure to afford the acetal **18** (4.23 g, 96%) as colorless needles. mp 172–174 °C; $[\alpha]_D$ −100 (*c* 1.0 in $CHCl_3$); δ_H (500 MHz, $CDCl_3$) 4.14 (1H, ddd, $J_{4,5}$ = 10.0, $J_{5,6}$ = 3.0, $J_{5,6}$ = 4.0 Hz, H5), 4.46 (1H, dd, $J_{6,6}$ = 12.0 Hz, H6a), 4.70–4.74 (2H, m, H2,6b), 5.63 (1H, d, $J_{1,2}$ = 2.0 Hz, H1), 5.73 (1H, dd, $J_{2,3}$ = 4.0, $J_{3,4}$ = 10.0 Hz, H3), 6.00 (1H, s, PhC*H*), 6.16 (1H, dd, H4), 7.18–8.12 (20H, m, Ph); δ_C (125 MHz, $CDCl_3$) 62.69, 66.37, 71.62, 71.73, 78.32 (5C, C2,3,4,5,6), 96.51 (1C, C1), 107.19 (1C, Ph*C*H), 127.79–136.29 (20C, Ph), 165.11, 166.10, 166.21 (3C, *C*OPh); HRMS (ESI+) *m*/*z* 603.1626 ($C_{34}H_{28}NaO_9$ $[M + Na]^+$ requires 603.1626).

1,2-Di-*O*-acetyl-3,4,6-tri-*O*-benzoyl-α-D-mannopyranose (19)

A solution of H_2SO_4 in Ac_2O (2% v/v, 12 mL) and the benzylidene acetal **18** (5.0 g, 8.61 mmol) was stirred at rt for 30 min. The mixture was diluted with EtOAc (50 mL) and the organic extract washed with sat. aq. $NaHCO_3$ (2 × 30 mL), dried ($MgSO_4$) and the solvents evaporated under reduced pressure. The residue was purified by flash chromatography (10:90 EtOAc/toluene) to give the diacetate **19** (2.97 g, 60%) as a yellow oil. $[\alpha]_D$ +19 (*c* 1.1 in $CHCl_3$; lit. [3] +36); δ_H (500 MHz, $CDCl_3$) 2.16, 2.25 (6H, 2 × s, 2 × CH_3), 4.40–4.47 (2H, m, H5,6a), 4.62 (1H, dd, $J_{5,6}$ = 2.0, $J_{6,6}$ = 11.5 Hz, H6b), 5.50 (1H, dd, $J_{1,2}$ = 2.0, $J_{2,3}$ = 3.5 Hz, H2), 5.79 (1H, dd, $J_{3,4}$ = 10.0 Hz, H3), 5.99 (1H, dd, $J_{4,5}$ = 10.0 Hz, H4), 6.23 (1H, d, H1), 7.34–8.04 (15H, m, Ph); δ_C (125 MHz, $CDCl_3$) 20.62, 20.92 (2C, CH_3), 62.86, 66.45, 68.72, 69.51, 70.87 (5C, C2,3,4,5,6), 90.62 (1C, C1), 128.33–133.52 (15C, Ph), 165.33, 165.55, 166.04 (3C, *C*OPh), 168.14, 169.50 (2C, *C*OCH_3).

4-Methylphenyl 2-*O*-acetyl-3,4,6-tri-*O*-benzoyl-1-thio-α-D-mannopyranoside (17)

A solution of $BF_3.Et_2O$ (196 μL, 1.54 mmol), the 1,2-diacetate **19** (0.89 g, 1.54 mmol) and thiocresol (0.25 g, 2.00 mmol) in dry toluene (20 mL) was stirred at rt for 2 d. The mixture was diluted with EtOAc (50 mL) and stirred for 10 min. The organic extract was washed with successively with water (2 × 50 mL) and sat. aq. $NaHCO_3$ (3 × 50 mL), then dried ($MgSO_4$) and concentrated under reduced pressure to give a residue, which was purified by flash chromatography (30:70 EtOAc/petroleum ether) to afford the thioglycoside **17** (0.86 g, 87%) as a colorless foam. $[\alpha]_D$ +85 (c 0.90 in CH_3Cl_3); (Found: C, 67.38; H, 5.01. $C_{36}H_{32}O_9$ requires C, 67.49; H, 5.03%); δ_H (500 MHz, $CDCl_3$) 2.14 (3H, s, CH_3), 2.27 (3H, s, ArCH_3), 4.54 (1H, dd, $J_{5,6}$ = 6.5, $J_{6,6}$ = 12.0 Hz, H6a), 4.58 (1H, dd, $J_{5,6}$ = 3.0, $J_{6,6}$ = 12.0 Hz, H6b), 4.96 (1H, m, H5), 5.55 (1H, d, $J_{1,2}$ = 1.5 Hz, H1), 5.73–5.76 (2H, m, H2,3), 5.92 (1H, dd, $J_{3,4}$ = 9.5, $J_{4,5}$ = 9.5 Hz, H4), 6.98–8.02 (19H, m, Ar, Ph); δ_C (125 MHz, $CDCl_3$) 20.77 (1C, CH_3), 21.12 (1C, ArCH_3), 63.38, 67.32, 69.74, 70.20, 71.29 (5C, C2,3,4,5,6), 86.12 (1C, C1),

128.29–138.33 (20C, Ar, Ph), 165.28, 165.56, 166.07 (3C, *C*OPh), 169.74 (1C, *C*OCH_3); HRMS (ESI+) *m/z* 663.1661 ($C_{36}H_{32}NaO_9S$ [M + Na]$^+$ requires 663.1659)

8-Azidooctyl 3,4-di-*O*-(2,3-dimethoxybutane-2,3-diyl)-α-D-mannopyranoside (15)

A mixture of the tetraol **12** (654 mg, 1.96 mmol), *p*-toluene sulfonic acid monohydrate (37 mg, 0.20 mmol), trimethyl orthoformate (860 μL, 7.84 mmol), and 2,3-butanedione (190 μL, 2.16 mmol) in dry MeOH (6 mL) was stirred at reflux overnight and then quenched with triethylamine (200 μL). The mixture was concentrated under reduced pressure and purified by flash chromatography (70:30 EtOAc/petroleum ether) to afford the diacetal **15** (702 mg, 80%) as a pale viscous oil. $[\alpha]_D$ +160 (*c* 1.3 in $CHCl_3$); (Found: C, 53.72; H, 8.41; N, 9.41. $C_{20}H_{37}N_3O_8$ requires C, 53.68; H, 8.33; N, 9.39%); δ_H (500 MHz, $CDCl_3$) 1.29, 1.32 (6H, s, CH_3), 1.31–1.63 (12H, m, $OCH_2(CH_2)_6CH_2N_3$), 3.25, 3.28 (6H, s, OCH_3), 3.26 (2H, t, J = 7.0 Hz, CH_2N_3), 3.40 (1H, ddd, J = 6.5, 9.5, 13.5 Hz, OCH_2CH_2), 3.66 (1H, ddd, J = 7.0, 9.5, 13.5 Hz, OCH_2CH_2), 3.75–3.84 (3H, m, H5,6a,6b), 3.90 (1H, dd, $J_{1,2}$ = 1.5, $J_{2,3}$ = 3.0 Hz, H2), 4.00 (1H, dd, $J_{3,4}$ = 10.5 Hz, H3), 4.08 (1H, dd, $J_{4,5}$ = 10.0 Hz, H4), 4.82 (1H, d, H1); δ_C (125 MHz, $CDCl_3$) 17.69, 17.77 (2C, CH_3), 25.99, 26.63, 28.79, 29.01, 29.22, 29.33 (6C, $OCH_2(CH_2)_6CH_2N_3$), 47.85, 48.09 (2C, OCH_3), 51.43 (1C, CH_2N_3), 61.41, 63.17, 67.91, 69.18, 69.84, 70.44 (6C, C2,3,4,5,6,OCH_2CH_2), 99.83, 100.00, 100.32 (C1,2’,3’); HRMS (ESI+) *m/z* 470.2472 ($C_{20}H_{37}N_3O_8$ [M + Na]$^+$ requires 470.2473).

8-Azidooctyl (2,3,4,6-tetra-*O*-benzoyl-α-D-mannopyranosyl)-(1→6)-2,3,4-tri-*O*-benzoyl-α-D-mannopyranoside (20)

TfOH (10 μL) was added to a mixture of the thioglycoside **21** [4] (424 mg, 0.604 mmol), the alcohol **14** (325 mg, 0.503 mmol), NIS (158 mg, 0.704 mmol) and freshly activated 4Å molecular sieves in dry CH_2Cl_2 (20 mL) at 0 °C under a N_2 atmosphere. The mixture was stirred for 10 min at which time the solution had turned dark purple. The reaction was quenched with sat. aq. $NaHCO_3$ and aq. $Na_2S_2O_3$ (0.5 M), and stirred for an additional 10

min. The mixture was filtered through Celite® and the organic phase separated and the aqueous phase extracted with CH_2Cl_2. The combined organic extracts were dried ($MgSO_4$), the solvent evaporated under reduced pressure and the residue purified by flash chromatography (30:70 EtOAc/petroleum ether) to afford the dimannoside **20** (517 mg, 84%) as a colorless oil. $[\alpha]_D$ −51 (*c* 1.1 in $CHCl_3$); (Found: C, 68.13; H, 5.51; N, 3.34. $C_{69}H_{65}N_3O_{18}$ requires C, 67.69; H, 5.35 N, 3.43%); δ_H (500 MHz, $CDCl_3$) 1.35–1.81 (12H, m, $OCH_2(CH_2)_6CH_2N_3$), 3.21 (2H, t, J = 7.0 Hz, CH_2N_3), 3.64 (1H, ddd, J = 6.5, 9.5, 13.0 Hz, OCH_2CH_2), 3.79 (1H, dd, $J_{5,6}$ = 2.0, $J_{6,6}$ = 11.0 Hz, $H6a^A$), 3.94 (1H, ddd, J = 6.5, 9.5, 13.5 Hz, OCH_2CH_2), 4.14 (1H, dd, $J_{5,6}$ = 5.5 Hz, $H6b^A$), 4.32 (1H, dd, $J_{5,6}$ = 4.0, $J_{6,6}$ = 12.0 Hz, $H6a^B$), 4.38–4.46 (2H, m, $H5^A,5^B$), 4.52 (1H, dd, $J_{5,6}$ = 2.5 Hz, $H6b^B$), 5.11 (1H, d, $J_{1,2}$ = 1.5 Hz), 5.15 (1H, d, $J_{1,2}$ = 1.5 Hz, $H1^A,1^B$), 5.75 (1H, dd, $J_{2,3}$ = 3.5 Hz), 5.78 (1H, d, $J_{2,3}$ = 3.0 Hz, $H2^A,2^B$), 5.95 (1H, dd, $J_{3,4}$ = 10.0 Hz), 5.99 (1H, dd, $J_{3,4}$ = 10.0 Hz, $H3^A,3^B$), 6.04 (1H, dd, $J_{4,5}$ = 10.0 Hz), 6.12 (1H, dd, $J_{4,5}$ = 10.0 Hz, $H4^A,4^B$), 7.28–8.17 (35H, m, Ph); δ_C (125 MHz, $CDCl_3$) 26.29, 26.82, 28.98, 29.26, 29.49, 29.56 (6C, $OCH_2(CH_2)_6CH_2N_3$), 51.58 (1C, CH_2N_3), 62.74, 66.83, 66.92, 67.27, 68.84, 69.06, 69.70, 70.23, 20.46, 70.88 (10C, $C(2,3,4,5,6)^A$, $(2,3,4,5,6)^B$), 97.81, 97.91 (2C, $C1^A,1^B$), 128.45–133.64 (25C, Ph), 165.31, 165.39, 165.60, 165.71, 165.74, 166.19 (6C, *C*OPh); HRMS (ESI+) *m*/*z* 1246.4152 ($C_{69}H_{65}N_3NaO_{18}$ $[M + Na]^+$ requires 1246.4155).

8-Azidooctyl (α-D-mannopyranosyl)-(1→6)-α-D-mannopyranoside (1)

A solution of 0.5 M NaOMe/MeOH (0.6 mL) and the dimannoside **20** (192 mg, 0.157 mmol) in CH_2Cl_2/MeOH (2:1, 4.7 mL) was stirred for 2 h at rt and then neutralized with Amberlite 120R resin (H^+ form). The mixture was filtered, the filtrate concentrated under reduced pressure, diluted with water (3 mL) and added to EtOAc (10 mL). The organic layer was extracted with water (3 × 5 mL) and the aqueous phase concentrated under reduced pressure to give a residue, which was purified by C_{18} reversed phase chromatography (100% H_2O to

40:60 MeOH/H_2O) to yield the disaccharide **1** (76.5 mg, 98%) as a colorless glass. δ_H (500 MHz, D_2O) 1.41–1.68 (12H, m, $OCH_2(CH_2)_6CH_2N_3$), 3.35 (2H, t, J = 7.0 Hz, CH_2N_3), 3.54 (1H, ddd, J = 6.5, 9.5, 12.5 Hz, OCH_2CH_2), 3.72–4.07 (13H, m, $H(2,3,4,5,6a,6b)^A$, $(2,3,4,5,6a,6b)^B$, OCH_2CH_2), 4.86 (1H, d, $J_{1,2}$ = 1.5 Hz), 4.93 (1H, d, $J_{1,2}$ = 1.5 Hz, $H1^A,1^B$); δ_C (125 MHz, D_2O; acetone) 26.35, 26.99, 29.07, 29.34, 29.52, 29.59 (6C, $(CH_2)_6CH_2N_3$), 51.88 (1C, CH_2N_3), 61.56, 66.15, 67.08, 67.32, 68.47, 70.71, 70.96, 71.35, 71.65, 71.81, 73.30 (11C, $C(2,3,4,5,6)^A$, $(2,3,4,5,6)^B$, OCH_2CH_2), 100.26, 100.71 (2C, $C1^A,1^B$); HRMS (ESI+) *m/z* 518.2319 ($C_{20}H_{37}N_3NaO_{11}$ $[M + Na]^+$ requires 518.2320).

8-Azidooctyl (2,3,4-tri-*O*-benzoyl-6-*O*-(*tert*-butyldiphenylsilyl)-α-D-mannopyranosyl)-(1→6)-2,3,4-tri-*O*-benzoyl-α-D-mannopyranoside (23)

TfOH (15 μL) was added to a mixture of the thioglycoside **22** (406 mg, 0.485 mmol), the alcohol **14** (197 mg, 0.305 mmol), NIS (100 mg, 0.427 mmol) and freshly activated powdered 4Å molecular sieves in dry CH_2Cl_2 (10 mL) at 0 °C under a N_2 atmosphere. The solution was stirred for 10 min during which time the solution turned dark purple. The reaction was quenched with sat. aq. $NaHCO_3$ and aq. $Na_2S_2O_3$ (0.5 M), and stirred for 10 min. The mixture was filtered through a layer of Celite® and the organic phase separated. The aqueous phase was extracted with CH_2Cl_2 (3 × 30 mL) and the combined organic phases dried ($MgSO_4$). The solvent was evaporated under reduced pressure and the residue purified by flash chromatography (10:90 EtOAc/petroleum ether) to afford the dimannoside **23** (337 mg, 99%) as a pale yellow oil. δ_H (500 MHz, $CDCl_3$) 1.33–1.79 (12H, m, $OCH_2(CH_2)_6CH_2N_3$), 1.00 (9H, s, *tert*-butyl), 3.18 (2H, t, J = 7.0 Hz, CH_2N_3), 3.59–4.06 (4H, m, $H(6a,6b)^A,6a^B$, $O(CH_2)CH_2$), 3.94 (1H, ddd, J = 6.5, 9.5, 13.5 Hz, OCH_2CH_2), 4.06 (1H, dd, $J_{5,6}$ = 6.0, $J_{6,6}$ = 11.0 Hz, $H6b^B$), 4.14, 4.38 (2H, 2 × br d, $H5^A,5^B$), 5.10 (1H, d, $J_{1,2}$ = 1.5 Hz), 5.13 (1H, d, $J_{1,2}$ = 1.5 Hz, $H1^A,1^B$), 5.71 (1H, dd, $J_{2,3}$ = 3.0 Hz), 5.73 (1H, dd, $J_{2,3}$ = 3.0 Hz, $H2^A,2^B$), 5.88 (1H, dd, $J_{3,4}$ = 10.0 Hz), 5.92 (1H, dd, $J_{3,4}$ = 10.0 Hz, $H3^A,3^B$), 5.97 (1H, dd, $J_{4,5}$ = 10.0 Hz), 6.18

(1H, dd, $J_{4,5}$ = 10.0 Hz, H4^A,4^B), 7.09–8.14 (40H, m, Ph); δ_C (125 MHz, $CDCl_3$) 19.24, 26.26, 26.71, 26.77, 28.93, 29.23, 29.46, 29.51 (10C, $OCH_2(CH_2)_6CH_2N_3$,$(CH_3)_3C$), 51.52 (1C, $CH_2CH_2N_3$), 62.34, 66.49, 66.65, 67.37, 68.71, 69.71, 70.50, 70.69, 70.81, 70.94, 71.47 (11C, C(2,3,4,5,6)^A, C(2,3,4,5,6)^B, OCH_2CH_2), 97.68, 97.71 (2C, C1^A,C1^B), 127.46–135.78 (48C, Ph), 165.31, 165.38, 165.55, 165.65, 165.66, 165.85 (6C, C=O); HRMS (ESI+) *m*/*z* 1380.5083 ($C_{78}H_{79}N_3NaO_{17}$ [M + Na]$^+$ requires 1380.5071).

8-Azidooctyl (2,3,4-tri-*O*-benzoyl-α-D-mannopyranosyl)-(1→6)-2,3,4-tri-*O*-benzoyl-α-D-mannopyranoside (24)

A solution of hydrogen fluoride-pyridine complex (70%, 0.89 mL) and the dimannoside **23** (1.00 g, 0.736 mmol) in THF (8 mL) was stirred at rt for 5 h. The solution was diluted with EtOAc, and the organic phase washed with sat. aq. $NaHCO_3$ (3 × 30 mL), dried ($MgSO_4$) and the solvent evaporated under reduced pressure. The residue was purified by flash chromatography (20:80 EtOAc/petroleum ether) to afford the dimannoside alcohol **24** (741 mg, 90%) as a pale yellow oil. $[\alpha]_D$ −55 (c 1.0 in $CHCl_3$); δ_H (500 MHz, $CDCl_3$) 1.37–1.80 (12H, m, $OCH_2(CH_2)_6CH_2N_3$), 2.55 (1H, t, $J_{OH,6}$ = 7.0 Hz, OH), 3.23 (2H, t, J = 7.0 Hz, CH_2N_3), 3.52–3.65 (3H, m, H(6a,6b)^A,OCH_2CH_2), 3.77 (1H, $J_{5,6}$ = 2.0, $J_{6,6}$ = 10.8 Hz, H6a^B), 3.93 (1H, ddd, J = 6.5, 9.5, 13.5 Hz, OCH_2CH_2), 4.02–4.08 (2H, m, H5^A,6b^B), 4.37 (1H, m, H5^B), 5.10 (1H, d, $J_{1,2}$ = 1.5 Hz), 5.15 (1H, d, $J_{1,2}$ = 1.5 Hz, H1^A,1^B), 5.72–5.74 (2H, m, H2^A,2^B), 5.81 (1H, dd, $J_{3,4}$ = 10.0, $J_{4,5}$ = 10.0 Hz), 5.93 (1H, dd, $J_{2,3}$ = 3.5 Hz), 6.01 (1H, dd, $J_{3,4}$ = 10.0, $J_{4,5}$ = 10.0 Hz, H4^A,4^B), 6.04 (1H, dd, $J_{2,3}$ = 3.5 Hz, H3^A,3^B), 7.28–8.18 (30H, m, Ph); δ_C (125 MHz, $CDCl_3$) 26.29, 26.85, 29.00, 29.27, 29.50, 29.56 (6C, $OCH_2(CH_2)_6CH_2N_3$), 61.21, 66.92, 67.30, 67.32, 68.85, 69.59, 69.74, 70.47, 70.57, 70.88, 71.15 (11C, C(2,3,4,5,6)^A, (2,3,4,5,6)^B, OCH_2CH_2), 97.86, 97.90 (2C, C1^A,1^B), 128.45–133.82 (30C, Ph), 165.31, 165.51, 165.72, 165.86, 166.74 (6C, *C*OPh); HRMS (ESI+) *m*/*z* 1142.3895 ($C_{62}H_{61}N_3NaO_{17}$ [M + Na]$^+$ requires 1142.3893).

8-Azidooctyl (2,3,4,6-tetra-*O*-benzoyl-α-D-mannopyranosyl)-(1→6)-(2,3,4-tri-*O*-benzoyl-α-D-mannopyranosyl)-(1→6)-2,3,4-tri-*O*-benzoyl-α-D-mannopyranoside (25)

TfOH (20 μL) was added to a mixture of the thioglycoside **21** [4] (242 mg, 0.344 mmol), the alcohol **24** (341 mg, 0.304 mmol), NIS (102 mg, 0.453 mmol) and freshly activated 4 Å molecular sieves in dry CH_2Cl_2 (20 mL) at 0 °C under a N_2 atmosphere. The mixture was stirred for 10 min at which time the solution had turned dark purple. The reaction was quenched with sat. aq. $NaHCO_3$ and aq. $Na_2S_2O_3$ (0.5 M) and stirred for 10 min. The mixture was filtered through Celite, the organic phase separated and the aqueous phase extracted with CH_2Cl_2. The combined organic phases were dried ($MgSO_4$), concentrated under reduced pressure and the residue was purified by flash chromatography (25:75 and then 30:70 EtOAc/petroleum ether) to afford the trimannoside **25** (425 mg, 82%) as a colorless oil. $[\alpha]_D$ −101 (*c* 1.0 in $CHCl_3$); δ_H (500 MHz, $CDCl_3$) 1.38–1.84 (12H, m, $OCH_2(CH_2)_6CH_2N_3$), 3.27 (2H, t, J = 7.0 Hz, CH_2N_3), 3.52–4.53 (8H, m, $H(6a,6b)^A$, $(6a,6b)^B$, $(6a,6b)^C$, OCH_2CH_2), 4.38–4.60 (3H, m, $H5^A,5^B,5^C$), 4.93 (1H, d, $J_{1,2}$ = 1.5 Hz), 5.22 (1H, d, $J_{1,2}$ = 1.5 Hz), 5.28 (1H, d, $J_{1,2}$ = 1.5 Hz, $H1^A,1^B,1^C$), 5.67 (1H, dd, $J_{2,3}$ = 3.0 Hz), 5.85 (1H, dd, $J_{2,3}$ = 3.0 Hz), 5.98 (1H, m, $H2^A,2^B,2^C$), 6.02–6.32 (6H, m, $H(3,4)^A$, $(3,4)^B$, $(3,4)^C$), 7.29–8.29 (50H, m, Ph); δ_C (125 MHz, $CDCl_3$) 26.09, 26.65, 28.80, 29.06, 29.32, 29.41 (6C, $OCH_2(CH_2)_6CH_2N_3$), 51.42 (1C, CH_2N_3), 62.42, 65.96, 66.40, 66.52, 66.75, 66.79, 68.60, 68.80, 69.39, 69.43, 70.04, 70.15, 70.29, 70.33, 70.58, 70.70 (16C, $C(2,3,4,5,6)^A$, $(2,3,4,5,6)^B$, $(2,3,4,5,6)^C$, OCH_2CH_2), 97.45, 97.80, 97.95 (3C, $C1^A,1^B,1^C$), 128.29–133.49 (50C, Ph), 165.05, 165.17, 165.21, 165.39, 165.47, 165.50, 165.54, 165.61, 165.79, 166.00 (10C, *C*OPh); HRMS (ESI+) *m*/*z* 1720.5481 ($C_{96}H_{87}N_3NaO_{26}$ $[M + Na]^+$ requires 1720.5470).

8-Azidooctyl (α-D-mannopyranosyl)-(1→6)-(α-D-mannopyranosyl)-(1→6)-α-D-mannopyranoside (2)

A solution of 1.5 M NaOMe/MeOH (0.35 mL) and the trimannoside **25** (0.29 g, 0.17 mmol) in MeOH/CH_2Cl_2 (2:1, 6 mL) was stirred for 2 h at rt and then neutralized with Amberlite 120R resin (H^+ form). The mixture was filtered and the filtrate concentrated under reduced pressure, diluted with water (10 mL) and poured into EtOAc (50 mL). The organic layer was extracted with water (3 × 10 mL) and the aqueous phase concentrated under reduced pressure to give a residue, which was purified by C_{18} reversed phase chromatography (100% H_2O to 40:60 MeOH/H_2O) to give the trisaccharide **2** (105 mg, 94%) as a colorless glass. $[\alpha]_D$ +78 (*c* 0.49 in H_2O); δ_H (500 MHz, D_2O) 1.37–1.68 (12H, m, $OCH_2(CH_2)_6CH_2N_3$), 3.34 (2H, t, J = 6.9 Hz, CH_2N_3), 3.58 (1H, ddd, J = 6.0, 10.0, 12.0 Hz, OCH_2CH_2), 3.66–4.00 (19H, m, OCH_2CH_2, $H(2,3,4,5,6a,6b)^A$, $(2,3,4,5,6a,6b)^B$, $(2,3,4,5,6a,6b)^C$), 4.87 (1H, d, $J_{1,2}$ = 1.5 Hz), 4.90 (1H, d, $J_{1,2}$ = 1.5 Hz), 4.93 (1H, d, $J_{1,2}$ = 1.5 Hz, $H1^A,1^B,1^C$); δ_C (125 MHz, D_2O) 25.66, 26.27, 28.35, 28.60, 28.69, 28.85 (6C, $OCH_2(CH_2)_6CH_2N_3$), 51.44 (1C, CH_2N_3), 61.15, 65.77, 65.87, 66.81, 66.95, 68.13, 70.20, 70.43, 70.78, 70.89, 71.11, 71.25, 72.92 (16C, $C(2,3,4,5,6)^A$, $(2,3,4,5,6)^B$, $(2,3,4,5,6)^C$, OCH_2CH_2), 99.49, 99.74, 100.14 (3C, $^1J_{C,H}$ = 173.1, 171.6, 172.8 Hz, $C1^A,1^B,1^C$); HRMS (ESI+) *m/z* 680.2847 ($C_{26}H_{47}N_3NaO_{16}$ $[M + Na]^+$ requires 680.2849).

8-Azidooctyl (2-*O*-acetyl-3,4,6-tri-*O*-benzoyl-α-D-mannopyranosyl)-(1→6)-2,3,4-tri-*O*-benzoyl-α-D-mannopyranoside (26)

From trichloroacetimidate 16:

$BF_3{\cdot}Et_2O$ (29 μL, 0.23 mmol) was added to a stirred mixture of the trichloroacetimidate **16** (0.554 g, 0.816 mmol), alcohol **14** (0.439 g, 0.680 mmol) and freshly activated 4 Å molecular sieves in dry CH_2Cl_2 (20 mL) at −10 °C under a N_2 atmosphere. The mixture was stirred for 2 h, diluted with CH_2Cl_2, quenched with sat. aq. $NaHCO_3$ (2 mL) and then stirred for a further

10 min. The mixture was filtered through a layer of Celite and the organic phase separated. The organic layer was washed with sat. aq. $NaHCO_3$ (30 mL), dried ($MgSO_4$) and the solvent evaporated under reduced pressure to yield a residue, which was purified by flash chromatography (first column: 10:90 EtOAc/toluene; second column: 25:75 then 30:70 EtOAc/petroleum ether) to yield the disaccharide **26** (0.598 g, 76%) as a colorless foam.

From thioglycoside 17:

TfOH (10 μL) was added to a stirred mixture of the thioglycoside **17** (373 mg, 0.582 mmol), alcohol **14** (313 mg, 0.485 mmol), NIS (153 mg, 0.679 mmol) and freshly activated 4 Å molecular sieves in dry CH_2Cl_2 (20 mL) at 0 °C under a N_2 atmosphere. The mixture was stirred for 1 h and then quenched with aq. $Na_2S_2O_3$ and sat. aq. $NaHCO_3$, and stirred for a further 10 min. The mixture was filtered through Celite®, the organic phase separated and the aqueous phase extracted with CH_2Cl_2 (2 × 30 mL). The combined organic extracts were dried ($MgSO_4$) and the solvent evaporated under reduced pressure to yield a residue, which was purified by flash chromatography (30:70 EtOAc/petroleum ether) to give the disaccharide **26** (404 mg, 72%) as a colorless foam. $[\alpha]_D$ −22 (*c* 1.0 in $CHCl_3$); (Found: C, 66.07; H, 5.46; N, 3.57. $C_{64}H_{63}N_3O_{18}$ requires C, 66.14; H, 5.46; N, 3.62%); δ_H (500 MHz, $CDCl_3$; $CHCl_3$) 1.37–1.80 (12H, m, $OCH_2(CH_2)_6CH_2N_3$), 2.11 (3H, s, OCH_3), 3.23 (2H, t, J = 7.0 Hz, CH_2N_3), 3.62 (1H, ddd, J = 6.5, 9.5, 13.0 Hz, OCH_2CH_2), 3.75 (1H, $J_{5,6}$ = 2.0, $J_{6,6}$ = 11.0 Hz, $H6a^A$), 3.93 (1H, ddd, J = 6.5, 9.5, 13.0 Hz, OCH_2CH_2), 4.11 (1H, dd, $J_{5,6}$ = 5.5 Hz, $H6b^A$), 4.31–4.41 (3H, m, $H5^A$,$(5,6a)^B$), 4.45 (1H, dd, $J_{5,6}$ = 2.5, $J_{6,6}$ = 12.0, $H6b^B$), 5.01 (1H, d, $J_{1,2}$ = 1.5 Hz, $H1^B$), 5.09 (1H, d, $J_{1,2}$ = 1.5 Hz, $H1^A$), 5.56 (1H, br d, $H2^B$), 5.74 (1H, dd, $J_{2,3}$ = 3.0 Hz, $H2^A$), 5.87–5.96 (3H, m), 6.04 (1H, dd, $J_{3,4}$ = 10.0, $J_{4,5}$ = 10.0 Hz, $H(3,4)^A$,$(3,4)^B$), 7.27–8.16 (30H, m, Ph); δ_C (125 MHz, $CDCl_3$; $CHCl_3$) 20.69 (1C, CH_3), 26.11, 26.66, 28.82, 29.09, 29.31, 29.38 (6C, $OCH_2(CH_2)_6CH_2N_3$), 51.42 (1C, CH_2N_3), 63.00, 66.62, 66.82, 67.07, 68.64, 68.85, 69.53, 69.75, 69.85, 70.27, 70.70 (11C, $C(2,3,4,5,6)^A$, $(2,3,4,5,6)^B$, OCH_2CH_2), 97.51,

97.72 (2C, C1^{A},1^{B}), 128.26–133.43 (30C, Ph), 165.09, 165.50, 165.53, 165.55, 165.69, 165.96 (6C, *C*OPh), 169.65 (1C, *C*OCH$_3$); HRMS (ESI+) *m/z* 1184.3995 ($C_{64}H_{63}N_3NaO_{18}$ [M + Na]$^+$ requires 1184.3999).

8-Azidooctyl (3,4,6-tri-*O*-benzoyl-α-D-mannopyranosyl)-(1→6)-2,3,4-tri-*O*-benzoyl-α-D-mannopyranoside (27)

Acetyl chloride (900 μL) was added to a stirred solution of the dimannoside **26** (620 mg, 0.533 mmol) in MeOH/CH$_2$Cl$_2$ (30 mL, 2:1) at 0 °C. The mixture was warmed to rt and stirred for 48 h under a N$_2$ atmosphere and then carefully neutralized with Et$_3$N. The solvents were evaporated under reduced pressure to give a residue, which was dissolved in EtOAc (50 mL), washed with successively with water (2 × 20 mL), 1 M HCl (10 mL), sat. aq. NaHCO$_3$ (20 mL) and brine (20 mL), dried (MgSO$_4$) and then concentrated under reduced pressure. The residue was purified by flash chromatography (10:90 EtOAc/toluene) to afford the dimannoside alcohol **27** (433 mg, 73%) as a colorless oil. $[\alpha]_D$ −22 (*c* 1.0 in CHCl$_3$); (Found: C, 66.43; H, 5.58; N, 3.66. $C_{62}H_{61}N_3O_{17}$ requires C, 66.48; H, 5.49; N, 3.75%); δ_H (500 MHz, CDCl$_3$) 1.37–1.79 (12H, m, OCH$_2$(C*H*$_2$)$_6$CH$_2$N$_3$), 2.21 (1H, br d, *J* = 4.5 Hz, OH), 3.23 (2H, t, *J* = 7.0 Hz, CH$_2$N$_3$), 3.61 (1H, ddd, *J* = 6.5, 9.5, 13.0 Hz, OC*H*$_2$CH$_2$), 3.79 (1H, dd, $J_{5,6}$ = 2.0, $J_{6,6}$ = 11.0 Hz, H6a^{A}), 3.90 (1H, ddd, *J* = 6.5, 9.5, 13.0 Hz, OC*H*$_2$CH$_2$), 4.12 (1H, dd, $J_{5,6}$ = 5.0 Hz, H6b^{A}), 4.30–4.40 (5H, m, H5^{A},(2,5,6a,6b)^{B}), 5.07 (1H, d, $J_{1,2}$ = 1.5 Hz), 5.10 (1H, d, $J_{1,2}$ = 1.5 Hz, H1^{A},1^{B}), 5.73 (1H, dd, $J_{2,3}$ = 3.5 Hz, H2^{A}), 5.77 (1H, dd, $J_{2,3}$ = 3.0, $J_{3,4}$ = 10.0 Hz, H3^{B}), 5.93 (1H, dd, $J_{4,5}$ 10.0 Hz, H4^{B}), 5.94 (1H, dd, $J_{3,4}$ = 10.0 Hz, H3^{A}), 6.09 (1H, dd, $J_{4,5}$ = 10.0 Hz, H4^{A}), 7.26–8.15 (30 H, m, Ph); δ_C (125 MHz, CDCl$_3$) 26.11, 26.66, 28.82, 29.09, 29.31, 29.38 (6C, OCH$_2$(*C*H$_2$)$_6$CH$_2$N$_3$), 51.44 (1C, CH$_2$N$_3$), 63.17, 66.35, 66.69, 67.04, 68.69, 68.76, 69.25, 69.66, 70.29, 70.76, 72.62 (11C, C(2,3,4,5,6)^{A}, (2,3,4,5,6)^{B}, O*C*H$_2$CH$_2$), 97.73, 99.63 (2C, C1^{A},1^{B}), 128.29–133.42 (30C, Ph), 165.29, 165.50, 165.56, 165.66, 166.05 (6C, *C*OPh); HRMS (ESI+) *m/z* 1142.3902 ($C_{62}H_{61}N_3NaO_{17}$ [M + Na] requires 1142.3293).

8-Azidooctyl (2,3,4-tri-*O*-benzoyl-α-D-mannopyranosyl)-(1→2)-(3,4,6-tri-*O*-benzoyl-α-D-mannopyranosyl)-(1→6)-2,3,4,6-tetra-*O*-benzoyl-α-D-mannopyranoside (28)

TfOH (5 μL) was added to a stirred mixture of the disaccharide alcohol **27** (410 mg, 0.353 mmol), thioglycoside **21** [4] (298 mg, 0.423 mmol), NIS (111 mg, 0.494 mmol) and freshly activated 4 Å molecular sieves in dry CH_2Cl_2 (30 mL) at 0 °C under a N_2 atmosphere. The mixture was stirred for 2 h at 0 °C and then warmed to rt and stirred for a further 1 h. The reaction was quenched with aq. $Na_2S_2O_3$ and sat. aq. $NaHCO_3$ and stirred for 10 min. The mixture was filtered through Celite® and the organic phase separated. The aqueous phase was extracted with CH_2Cl_2 (3 × 20 mL) and the combined organic extracts were dried ($MgSO_4$). The solvent was evaporated under reduced pressure to yield a residue, which was purified by flash chromatography (1:6:3 EtOAc/toluene/petroleum ether) to afford the trisaccharide **28** (364 mg, 61%) as a colorless glass. $[\alpha]_D$ −54 (*c* 0.96 in $CHCl_3$); (Found: C, 67.90; H, 5.17; N, 2.48. $C_{96}H_{87}N_3O_{26}$ requires C, 67.88; H, 5.16; N, 2.47%); δ_H (500 MHz, $CDCl_3$) 1.40–1.83 (12H, m, $OCH_2(CH_2)_6CH_2N_3$), 3.24 (2H, t, J = 7.0 Hz, CH_2N_3), 3.65 (1H, ddd, J = 6.5, 9.5, 13.0 Hz, OCH_2CH_2), 3.79 (1H, dd, $J_{5,6}$ = 2.5, $J_{6,6}$ = 11.0 Hz, $H6a^A$), 3.95 (1H, ddd, J = 6.5, 9.5, 13.0 Hz, OCH_2CH_2), 4.09 (1H, dd, $J_{5,6}$ = 4.5 Hz, $H6b^A$), 4.36–4.54 (6H, m, $H5^A$,$(2,5,6a,6b)^B$,$6a^C$), 4.60 (1H, m, $H5^C$), 4.67 (1H, dd, $J_{5,6}$ = 2.5 Hz, $J_{6,6}$ = 12.0 Hz, $H6b^C$), 5.07 (1H, d, $J_{1,2}$ = 1.5 Hz), 5.16 (1H, d, $J_{1,2}$ = 1.5 Hz), 5.31 (1H, d, $J_{1,2}$ = 1.5 Hz, $H1^A$,1^B,1^C), 5.75 (1H, dd, $J_{2,3}$ = 3.5 Hz), 5.88 (1H, dd, $J_{2,3}$ = 3.0 Hz, $H2^A$,2^C), 5.96-6.18 (6H, m, $H(3,4)^A$,$(3,4)^B$,$(3,4)^C$), 7.26–8.13 (50H, m, Ph); δ_C (125 MHz, $CDCl_3$) 26.06, 26.60, 28.76, 29.03, 29.27, 29.35 (6C, $OCH_2(CH_2)_6CH_2N_3$), 51.35 (1C, CH_2N_3), 62.60, 63.44, 66.31, 66.62, 67.17, 67.22, 68.65, 68.82, 69.59, 69.61, 69.76, 70.06, 70.23, 70.61, 70.75, 77.58 (16C, $C(2,3,4,5,6)^A$, $(2,3,4,5,6)^B$, $(2,3,4,5,6)^C$, OCH_2CH_2), 97.64, 98.47, 99.78 (3C, $^1J_{C,H}$ = 172.1, 172.4, 172.0 Hz, $C1^A$,1^B,1^C), 128.15–133.33 (60C, Ph), 164.75, 164.93, 165.17, 165.38, 165.43, 165.45, 165.49, 165.55, 165.91, 166.15 (10C, *C*OPh); HRMS *m*/*z* (ESI+) 1720.5475

($C_{96}H_{87}N_3NaO_{26}$ [M + Na] requires 1720.5470).

8-Azidooctyl (α-D-mannopyranosyl)-(1→2)-(α-D-mannopyranosyl)-(1→6)-α-D-mannopyranoside (3)

A solution of the trimannoside **28** (58 mg, 34 μmol) and 1.5 M NaOMe/MeOH (70 μL) in CH_2Cl_2/MeOH (2:1, 1 mL) was stirred for 1 h at rt and then neutralized with Amberlite 120R resin (H^+ form). The mixture was filtered, the filtrate concentrated under reduced pressure, diluted with water (5 mL) and poured into EtOAc (10 mL). The organic layer was extracted with water (3 × 3 mL) and the aqueous phase concentrated under reduced pressure to give a residue, which was purified by C_{18} reversed phase chromatography (100% H_2O to 40:60 MeOH/H_2O) to give the trisaccharide **3** (22 mg, 98%) as a colorless glass. $[\alpha]_D$ +76 (*c* 0.98 in MeOH); δ_H (500 MHz, D_2O) 1.39–1.69 (12H, m, $OCH_2(CH_2)_6CH_2N_3$), 3.36 (2H, t, J = 7.0 Hz, CH_2N_3), 3.6 (1H, ddd, J = 6.0, 10.0, 12.0 Hz, OCH_2CH_2), 3.68 (1H, dd, $J_{3,4}$ = 9.5, $J_{4,5}$ = 9.5 Hz), 3.74–4.01 (16H, m), 4.03 (1H, dd, $J_{1,2}$ = 1.5 Hz, $J_{2,3}$ = 3.0 Hz), 4.11 (1H, dd, $J_{1,2}$ = 2.0, $J_{2,3}$ = 3.0 Hz, $H(2,3,4,5,6a,6b)^A$, $(2,3,4,5,6a,6b)^B$, $(2,3,4,5,6a,6b)^C$, OCH_2CH_2), 4.89 (1H, d, $J_{1,2}$ = 1.0 Hz), 5.07 (1H, d, $J_{1,2}$ = 1.5 Hz), 5.18 (1H, d, $J_{1,2}$ = 1.5 Hz, $H1^A,1^B,1^C$); δ_C (125 MHz, D_2O; acetone) 25.92, 26.53, 28.62, 28.85, 28.89, 29.09, (6C, $OCH_2(CH_2)_6CH_2N_3$), 51.88 (1C, CH_2N_3), 61.60, 61.77, 66.67, 67.27, 67.51, 67.62, 68.66, 70.63, 70.74, 70.92, 71.01, 71.53, 71.77, 73.36, 73.86, 79.42 (16C, $C(2,3,4,5,6)^A$, $C(2,3,4,5,6)^B$, $C(2,3,4,5,6)^C$, OCH_2CH_2), 98.75, 100.54, 102.98 (3C, $C1^A,1^B,1^C$); HRMS (ESI+) *m/z* 680.2844 ($C_{26}H_{47}N_3$-NaO_{16} [M + Na] requires 680.2849).

Azidooctyl 2,6-di-*O*-(2,3,4,6-tetra-*O*-benzoyl-α-D-mannopyranosyl)-3,4-di-*O*-(2,3-dimethoxybutane-2,3-diyl)-α-D-mannopyranoside (29)

TfOH (9 μl, 0.10 mmol) was added to a stirred mixture of thioglycoside **21** [4] (1.44 g, 2.04 mmol), the diol **15** (228 mg, 0.509 mmol), NIS (690 mg, 3.07 mmol) and freshly activated 4 Å molecular sieves in dry CH_2Cl_2 (50 mL) at 0 °C under a N_2 atmosphere. The

mixture was stirred for 1 h at 0 °C, then warmed to rt and stirred for an additional 1 h. The reaction was quenched with sat. aq. $NaHCO_3$ and aq. $Na_2S_2O_3$ (0.5 M), and stirred for 10 min. The mixture was filtered through a layer of Celite® and the organic phase separated. The aqueous phase was extracted with CH_2Cl_2 and the combined organic phases were dried ($MgSO_4$). The solvent was evaporated under reduced pressure. Flash chromatography (25:75 to 30:70 EtOAc/petroleum ether) of the residue afforded the trisaccharide **29** (551 mg, 68%) as a colorless foam. $[\alpha]_D$ −0.8 (*c* 1.0 in $CHCl_3$); (Found: C, 65.91; H, 5.60; N, 2.56. $C_{88}H_{89}N_3O_{26}$ requires C, 65.87; H, 5.59; N, 2.62%); δ_H (500 MHz, $CDCl_3$) 1.25, 1.32 (6H, s, CH_3), 1.31–1.66 (12H, m, $OCH_2(CH_2)_6CH_2N_3$), 3.19 (2H, t, J = 7.0 Hz, CH_2N_3), 3.25, 3.48 (6H, s, OCH_3), 3.46–3.51 (1H, m, OCH_2CH_2), 3.81 (1H, ddd, J = 7.0, 9.5, 14.0 Hz, OCH_2CH_2), 4.02–4.06 (2H, m, H2,5), 4.1–4.14 (2H, m, H3,6), 4.16 (1H, dd, $J_{5,6}$ = 5.5 Hz, $J_{6,6}$ = 12.0 Hz, H6), 4.42 (1H, dd, $J_{3,4}$ = 10.0, $J_{4,5}$ = 10.0 Hz, H4), 4.52 (1H, dd, $J_{5,6}$ = 4.0, $J_{6,6}$ = 12.0 Hz, H6), 4.58 (1H, dd, $J_{5,6}$ = 4.0, $J_{6,6}$ = 12.0 Hz, H6), 4.65 (1H, m, H5), 4.76 (1H, dd, $J_{5,6}$ = 2.5, $J_{6,6}$ = 12.0 Hz, H6), 4.81 (1H, m, H5), 4.85 (1H, dd, $J_{5,6}$ = 2.5, $J_{6,6}$ = 12.0 Hz, H6), 5.01 (1H, s, H1), 5.48 (1H, d, $J_{1,2}$ = 1.5 Hz, H1), 5.50 (1H, d, $J_{1,2}$ = 2.0 Hz, H1), 5.93 (1H, dd, $J_{1,2}$ = 2.0, $J_{2,3}$ = 3.0 Hz, H2), 6.09 (1H, dd, $J_{2,3}$ = 3.0, $J_{3,4}$ = 10.0 Hz, H3), 6.09 (1H, dd, $J_{2,3}$ = 3.0, $J_{3,4}$ = 10.0 Hz, H3), 6.12 (1H, dd, $J_{1,2}$ = 2.0, $J_{2,3}$ = 3.0 Hz, H2), 6.16 (1H, dd, $J_{3,4}$ = 10.0, $J_{4,5}$ = 10.0 Hz, H4), 6.20 (1H, dd, $J_{3,4}$ = 10.0, $J_{4,5}$ = 10.0 Hz, H4), 6.91-8.17 (40 H, m, Ph); δ_C (125 MHz, $CDCl_3$; $CHCl_3$) 17.40, 17.63(2C, CH_3), 26.09, 26.61, 28.75, 29.03, 29.31, 29.50 (6C, $OCH_2(CH_2)_2CH_2N_3$), 48.09 (2C, OCH_3), 51.36 (CH_2N_3), 62.91, 62.92, 63.16, 65.66, 66.81, 67.44, 68.02, 68.82, 69.00, 69.24, 69.62, 70.18, 70.36, 70.97, 71.06, 76.69 (16C, 3 × C(2,3,4,5,6), OCH_2CH_2), 97.90, 99.41, 99.45 (3C, $^1J_{C,H}$ = 172.1, 172.1, 174.2 Hz, 3 × C1), 99.76, 100.10 (2C, C2',C3'), 127.77–133.24 (40C, Ph), 164.63, 164.94, 165.11, 165.12, 165.51, 166.15, 166.21 (8C, *C*OPh); HRMS (ESI+) *m/z* 1626.5653 ($C_{88}H_{89}N_3O_{26}$ $[M + Na]^+$ requires 1626.5627).

Azidooctyl 2,6-di-*O*-α-D-mannopyranosyl-α-D-mannopyranoside (4)

A mixture of TFA/H_2O (9:1, 1.5 mL) and the trisaccharide **29** (78 mg, 0.048 mmol) was stirred for 10 min at rt. Toluene (10 mL) was added and the mixture concentrated under reduced pressure to give azidooctyl 2,6-di-*O*-(2,3,4,6-tetra-*O*-benzoyl-α-D-mannopyranosyl)-α-D-mannopyranoside (72 mg, 99%) as a yellow oil, which was used immediately in the next step. A small portion of the product was further purified by flash chromatography (40:60 EtOAc/petroleum ether) for characterization: $[\alpha]_D$ −27 (*c* 0.95 in $CHCl_3$); δ_H (500 MHz, $CDCl_3$) 1.28–1.56 (12H, m, $OCH_2(CH_2)_6CH_2N_3$), 3.20 (2H, t, J = 7.0 Hz, CH_2N_3), 3.34 (1H, ddd, J = 6.5, 9.5, 13.0 Hz, OCH_2CH_2), 3.38 (1H, d, J = 3.5 Hz, OH), 3.55 (1H, d, J = 8.0 Hz, OH), 3.68 (1H, ddd, J = 7.0, 9.5, 13.5 Hz, OCH_2CH_2), 3.84–3.91 (2H, m, H5,6), 4.07-4.34 (4H, m, H2,3,5,6), 4.56 (1H, dd, $J_{5,6}$ = 3.5, $J_{6,6}$ = 12.0 Hz, H6), 4.59 (1H, dd, $J_{5,6}$ = 5.0, $J_{6,6}$ = 13.5 Hz, H6), 4.66 (1H, m, H5), 4.75–4.80 (3H, m, H4,6,6), 5.15 (1H, br s, H1), 5.27 (1H, d, $J_{1,2}$ = 1.0 Hz, H1), 5.41 (1H, d, $J_{1,2}$ = 1.5 Hz, H1), 5.88 (1H, m, H2), 5.98 (1H, dd, $J_{2,3}$ = 4.0, $J_{3,4}$ = 10.5 Hz, H3), 5.99 (1H, dd, $J_{2,3}$ = 3.5, $J_{3,4}$ = 9.9 Hz, H3), 6.06 (1H, m, H2), 6.21 (1H, $J_{3,4}$ = 9.5, $J_{4,5}$ = 9.5 Hz, H4), 6.23 (1H, $J_{3,4}$ = 10.0, $J_{4,5}$ = 9.9 Hz, H4); δ_C (125 MHz, $CDCl_3$) 26.04, 26.64, 28.78, 29.05, 29.29, 29.48 (6C, $OCH_2(CH_2)_6CH_2N_3$), 51.41 (1C, CH_2N_3), 66.35, 66.68, 66.82, 67.72, 68.08, 68.83, 69.75, 70.34, 70.35, 70.76, 70.84, 71.14, 71.66, 81.22 (16C, 3 × C(2,3,4,5,6), OCH_2CH_2), 97.58, 98.93, 100.59 (3C, 3 × C1), 127.68–133.29 (40C, Ph), 165.00, 165.14, 165.30, 165.57, 165.60, 165.77, 166.20, 166.23 (8C, *C*OPh); HRMS (ESI+) *m*/*z* 1512.4965 ($C_{82}H_{79}N_3O_{24}$ $[M + Na]^+$ requires 1512.4946).

A solution of 1.5 M NaOMe (200 μL) and the crude diol (326 mg, 0.219 mmol) in CH_2Cl_2/MeOH (2:1, 6 mL) was stirred for 1 h at rt. The solution was neutralized with Amberlite 120R resin (H^+ form) and the mixture filtered. The filtrate was concentrated under reduced pressure, diluted with water (10 mL) and poured into EtOAc (50 mL). The organic

layer was extracted with water (3 × 10 mL) and the aqueous phase concentrated to afford the trisaccharide **4** (144 mg, 100%) as a colorless glass. A small portion of the trisaccharide was further purified by C_{18} reversed phase chromatography (100% H_2O to 40:60 MeOH/H_2O): $[\alpha]_D$ +73 (*c* 0.99 in MeOH); (Found: C, 47.50; H, 7.26; N, 6.40. $C_{26}H_{47}N_3O_{16}$ requires C, 47.48; H, 7.20; N, 6.39%); δ_H (500 MHz, d_4-methanol) 1.36–1.62 (12H, m, $OCH_2(CH_2)_6CH_2N_3$), 3.28 (2H, m, CH_2N_3), 3.44 (1H, ddd, J = 6.3, 9.7, 12.5 Hz, OCH_2CH_2), 3.56–4.00 (19H, m, 3 × H(2,3,4,5,6,6), OCH_2CH_2), 4.93 (1H, d, $J_{1,2}$ = 1.5 Hz), 4.94 (1H, d, $J_{1,2}$ = 1.5 Hz), 5.08 (1H, d, $J_{1,2}$ = 1.5 Hz, 3 × H1); δ_C (125 MHz, d_4-methanol) 27.25, 27.76, 29.88, 30.18, 30.36, 30.59 (6C, $OCH_2(CH_2)_6CH_2N_3$), 52.44 (1C, CH_2N_3), 62.97, 66.24, 68.41, 68.63, 68.76, 71.89, 71.97, 72.06, 72.43, 72.57, 73.72, 74.53, 74.97, 81.37 (16C, 3 × C(2,3,4,5,6), OCH_2CH_2), 99.89, 101.56, 104.42 (3C, 3 × C1); HRMS (ESI+) *m/z* 680.2852 ($C_{26}H_{47}N_3O_{16}$ $[M + Na]^+$ requires 680.2849).

4-[(7-Nitrobenzofurazan-4-yl)aminomethyl]-triazol-1-yloctyl (α-D-mannopyranosyl)-(1→6)-α-D-mannopyranoside (5)

Solutions of $CuSO_4$ (1 mL, 0.35 mg/mL, 2.18 μmol in 1:1 THF/H_2O) and sodium ascorbate (1 mL, 2.15 mg/mL, 10.9 μmol in 1:1 THF/H_2O) were added to a solution of the trisaccharide **1** (27 mg, 54.5 μmol), alkyne **30** (13 mg, 59.9 μmol) and TBTA (1.45 mg, 2.73 μmol) in THF/H_2O (1:1, 4 mL) at rt. The resultant solution was stirred for 1 h, then concentrated to dryness and purified by reversed phase chromatography (100% H_2O to 50% MeOH/H_2O) to give the NBD-disaccharide **5** (35 mg, 90%) as an orange syrup. $[\alpha]_D$ +45 (*c* 1.13 in MeOH); δ_H (500 MHz, d_4-methanol) 1.30–1.19 (12H, m, $OCH_2(CH_2)_6CH_2N$), 3.38 (1H, ddd, J = 6.5, 9.5, 12.5 Hz, OCH_2CH_2), 3.63–3.91 (13H, $H(2,3,4,5,6a,6b)^A$, $(2,3,4,5,6a,6b)^B$, OCH_2CH_2), 4.39 (2H, t, J = 7.5 Hz, CH_2CH_2N), 4.69 (1H, d, $J_{1,2}$ = 1.5 Hz), 4.82 (1H, d, $J_{1,2}$ = 1.5 Hz, $H1^A,1^B$), 6.43 (1H, d, J = 8.5 Hz, CH-CH=CNO_2), 8.05 (1H, s, triazole H), 8.48 (1H, d, J = 8.5 Hz, =CH-CH=CNO_2), CH_2NH obscured by HOD at 4.85 ppm; δ_C (125 MHz, d_4-

methanol) 27.15, 27.32, 29.91, 30.16, 30.44, 31.19 (6C, $OCH_2(CH_2)_6CH_2N$), 39.77 (1C, CH_2NH), 51.48 (1C, CH_2CH_2N), 62.86, 67.44, 68.45, 68.60, 68.63, 72.13, 72.1, 72.68, 72.86, 73.08, 74.32 (11C, C(2,3,4,5,6)^A, (2,3,4,5,6)^B, OCH_2CH_2), 100.64 (1C, =CH-CH=CNO_2), 101.34, 101.58 (2C, C1^A,1^B), 124.08, 124.63, 138.13, 144.49, 145.39, 145.93 (7C, Ar); HRMS (ESI+) *m/z* 736.2759 ($C_{29}H_{43}N_7NaO_{14}$ $[M + Na]^+$ requires 736.2760).

4-[(7-Nitrobenzofurazan-4-yl)aminomethyl]-triazol-1-yloctyl (α-D-mannopyranosyl)-(1→6)-(α-D-mannopyranosyl)-(1→6)-α-D-mannopyranoside (6)

Solutions of $CuSO_4$ (1 mL, 0.17 mM, 1.7 μmol in 1:1 THF/H_2O) and sodium ascorbate (1 mL, 8.5 mM, 8.5 μmol in 1:1 THF/H_2O) were added to a solution of the trisaccharide **2** (28 mg, 42.6 μmol), alkyne **30** (11 mg, 51.1 μmol) and TBTA (1.1 mg, 2.13 μmol) in THF/H_2O (1:1, 4 mL) at rt. The resultant solution was stirred for 1 h, then concentrated to dryness and purified by reversed phase chromatography (100% H_2O to 50% MeOH/H_2O) to give NBD-trisaccharide **6** (35.3 mg, 95%) as an orange syrup. $[\alpha]_D$ +54 (*c* 0.54 in H_2O); δ_H (500 MHz, d_4-;methanol) 1.26–1.90 (12H, m, $OCH_2(CH_2)_6CH_2N$), 3.38 (1H, ddd, J = 6.1, 9.7, 12.5 Hz, OCH_2CH_2), 3.58-3.87 (19H, m, H(2,3,4,5,6a,6b)^A, (2,3,4,5,6a,6b)^B, (2,3,4,5,6a,6b)^C, OCH_2CH_2), 4.39 (2H, t, J = 7.0 Hz, CH_2CH_2N), 4.71 (1H, d, $J_{1,2}$ = 1.0 Hz), 4.78 (1H, d, $J_{1,2}$ = 1.5 Hz), 4.86 (3H, d, $J_{1,2}$ = 1.5 Hz, H1^A,1^B,1^C), 4.85 (2H, br s, CH_2NH), 6.44 (1H, d, J = 9.0 Hz, =CH-CH=CNO_2), 8.04 (1H, s, triazole H), 8.48 (1H, d, J = 9.0 Hz, =CH-CH=CNO_2); δ_C (125 MHz, d_4-methanol) 27.15, 27.33, 29.92, 30.15, 30.44, 31.20 (6C, $OCH_2(CH_2)_6CH_2N$), 39.78 (1C, CH_2NH), 51.49 (1C, CH_2CH_2N), 62.91, 67.10, 67.38, 68.42, 68.66, 72.03, 72.23, 72.28, 72.51, 72.87, 72.90, 73.02, 74.43 (16C, C(2,3,4,5,6)^A, (2,3,4,5,6)^B, (2,3,4,5,6)^C, OCH_2CH_2), 100.83, 101.07, 101.43 (3C, C1^A,1^B,1^C), 100.60 (1C, =CH-CH=CNO_2), 124.08, 124.63, 138.14, 144.50, 145.40, 145.94 (7C, Ar); HRMS (ESI+) *m/z* 898.3295 ($C_{35}H_{53}N_7NaO_{19}$ $[M + Na]^+$ requires 898.3288).

4-[(7-Nitrobenzofurazan-4-yl)aminomethyl]-triazol-1-yloctyl (α-D-mannopyranosyl)-(1→2)-(α-D-mannopyranosyl)-(1→6)-α-D-mannopyranoside (7)

Solutions of $CuSO_4$ (1 mL, 0.252 mg/mL, 1.58 μmol in 1:1 THF/H_2O) and sodium ascorbate (1 mL, 1.57 mg/mL, 7.9 μmol in 1:1 THF/H_2O) were added to a solution of the trisaccharide **3** (26.0 mg, 38.0 μmol), alkyne **30** (9.5 mg, 43.5 μmol) and TBTA (1.0 mg, 1.98 μmol) in THF/H_2O (1:1, 4 mL) at rt. The resultant solution was stirred for 1 h, then concentrated to dryness and purified by reversed phase chromatography (100% H_2O to 40% MeOH/H_2O) to give NBD-trisaccharide **7** (32.4 mg, 94%) as an orange syrup. $[\alpha]_D$ +52 (*c* 1.14 in H_2O); δ_H (500 MHz, d_4-methanol) 1.26–1.90 (12H, m, $OCH_2(CH_2)_6CH_2N$), 3.39 (1H, ddd, *J* 6.5, 10.0, 12.5 Hz, OCH_2CH_2), 3.58–3.92 (19H, m), $H(2,3,4,5,6a,6b)^A$, $(3,4,5,6a,6b)^B$, $(2,3,4,5,6a,6b)^C$, OCH_2CH_2), 3.99 (1H, dd, $J_{1,2}$ = 1.5, $J_{2,3}$ = 3.0 Hz, $H2^B$), 4.39 (2H, t, *J* = 7.0 Hz, CH_2CH_2N), 4.71 (1H, d, $J_{1,2}$ = 1.5 Hz), 4.98 (1H, d, $J_{1,2}$ = 1.5 Hz), 5.13 (1H, d, $J_{1,2}$ = 1.5 Hz, $H1^A,1^B,1^C$), 6.45 (1H, d, *J* = 9.0 Hz, =C*H*-CH-CNO_2), 8.04 (1H, s, triazole H), 8.50 (1H, d, *J* = 9.0 Hz, =CH-C*H*=CNO_2), CH_2NH obscured by HOD at 4.85 ppm; δ_C (125 MHz, d_4-methanol) 27.15, 27.34, 29.93, 30.18,30.45, 31.20 (6C, $OCH_2(CH_2)_6CH_2N$), 39.77 (1C, CH_2NH), 51.48 (1C, CH_2CH_2N), 62.98, 63.08, 67.63, 68.46, 68.55, 68.75, 69.01, 71.90, 72.15, 72.21, 72.41, 72.82, 73.24, 74.35, 74.92, 80.58 (16C, $C(2,3,4,5,6)^A$, $(2,3,4,5,6)^B$, $(2,3,4,5,6)^C$, OCH_2CH_2), 99.20, 101.59. 104.16 (3C, $C1^A,1^B,1^C$), 100.68 (1C, =*C*H-CH=CNO_2), 124.07, 124.63, 138.16, 144.49, 145.41, 145.95 (7C, Ar); HRMS (ESI+) *m/z* 898.3291 ($C_{35}H_{53}N_7NaO_{19}$ $[M + Na]^+$ requires 898.3288).

8-(2-Ethoxycylobutene-3,4-dione-1-ylamino)octyl (α-D-mannopyranosyl)-(1→6)-(α-D-mannopyranosyl)-(1→6)-α-D-mannopyranoside (8)

A mixture of PPh_3 (27 mg, 0.11 mmol) and the trisaccharide **2** (29 mg, 0.044 mmol) in THF/H_2O (3:1, 2.4 mL) was stirred overnight at rt and then diluted with water (1 mL). The mixture was concentrated under reduced pressure to remove THF and the residue diluted with

EtOAc (10 mL). The organic phase was extracted with water (3 × 3 mL), the combined aqueous extracts washed with EtOAc (5 mL) and then concentrated to dryness to give the crude amine as a colorless glass (28 mg, quant). Diethyl squarate (4.7 μL, 0.030 mmol) was added to a stirred solution of the crude amine (14 mg, 0.022 mmol) in EtOH/H_2O/phosphate buffer pH 8 (2:1:1, 1.2 mL) at rt. The mixture was stirred for 2 h and then concentrated under reduced pressure. The residue was purified by reversed phase chromatography (100% H_2O to 40% MeOH/H_2O) to give the ethyl squaramyl adduct **8** (14 mg, 84% over 2 steps) as a colorless glass. δ_H (500 MHz, D_2O) 1.37–1.67 (12H, m, $OCH_2(CH_2)_6CH_2$), 1.48 (3H, m, OCH_2CH_3), 3.53 (1H, t, *J* = 6.5 Hz, CH_2NH), 3.59 (1H, ddd, *J* = 6.0, 10.0, 12.0 Hz), 3.63–4.01 (20H, m, H(2,3,4,5,6a,6b)^A, (2,3,4,5,6a,6b)^B, (2,3,4,5,6a,6b)^C, OCH_2CH_2,CH_2NH), 4.74–4.83 (2H, m, OCH_2CH_3), 4.88, 4.91, 4.95 (3H, s, H1^A,1^B,1^C); HRMS (ESI+) *m*/*z* 778.3105 ($C_{32}H_{53}NNaO_{19}$ [M + Na]$^+$ requires 778.3104). The ^{13}C NMR spectrum for this compound was poorly resolved. We attribute this to the presence of tautomeric isomers.

8-(2-Ethoxycylobutene-3,4-dione-1-ylamino)octyl (α-D-mannopyranosyl)-(1→2)-(α-D-mannopyranosyl)-(1→6)-α-D-mannopyranoside (9)

A solution of PPh_3 (33 mg, 0.128 mmol) and the trisaccharide **3** (21 mg, 0.0319 mmol) in THF/H_2O (3:1, 1.2 mL) was stirred overnight at rt and then diluted with water (1 mL). The mixture was concentrated under reduced pressure to remove THF and the residue diluted with EtOAc (10 mL). The organic phase was extracted with water (3 × 3 mL), the combined aqueous extracts washed with EtOAc (5 mL) and then concentrated to dryness to give the crude amine (20 mg). This residue was dissolved in EtOH/H_2O/phosphate buffer pH 8 (2:1:1, 1.2 mL) and then diethyl squarate (5.5 μL, 0.0351 mmol) was added. The mixture was stirred for 2 h, concentrated to dryness and then purified by reversed phase chromatography (100% H_2O to 40% MeOH/H_2O) to give the ethyl squaramyl adduct **9** (21 mg, 87% over 2 steps) as a colorless oil. δ_H (500 MHz, D_2O) 1.37–1.66 (12H, m, $OCH_2(CH_2)_6CH_2NH$), 1.48 (3H, m

OCH_2CH_3), 3.53 (1H, t, J = 7.0 Hz, CH_2NH), 3.58 (1H, ddd, J = 6.0, 10.0, 12.0 Hz, OCH_2CH_2), 3.63–4.02 (19H, m), 4.11 (1H, dd, $J_{1,2}$ = 2.0, $J_{2,3}$ = 3.0 Hz, H(2,3,4,5,6a,6b)^A, (2,3,4,5,6a,6b)^B, (2,3,4,5,6a,6b)^C, OCH_2CH_2,CH_2NH), 4.71–4.81 (2H, m, OCH_2CH_3), 4.88 (1H, d, $J_{1,2}$ = 1.0 Hz), 5.06 (1H, s), 5.18 (1H, s, H1^A,1^B,1^C); HRMS (ESI+) *m/z* 778.3106 ($C_{32}H_{53}NNaO_{19}$ $[M + Na]^+$ requires 778.3104). The ^{13}C NMR spectrum for this compound was poorly resolved. We attribute this to the presence of tautomeric isomers.

8-(2-Ethoxycylobutene-3,4-dione-1-ylamino)octyl 2,6-di-*O*-α-D-mannopyranosyl-α-D-mannopyranoside (10)

A solution of Ph_3P (20 mg, 0.076 mmol) and the trisaccharide **4** (12.5 mg, 0.019 mmol) in THF/H_2O (3:1, 0.8 mL) was stirred overnight at rt and then diluted with water (1 mL). The mixture was concentrated under reduced pressure to remove THF and the residue diluted with EtOAc (10 mL). The organic phase was extracted with water (3 × 3 mL), the combined aqueous extracts washed with EtOAc (5 mL) and then concentrated to give the crude amine (12.5 mg) as a colorless glass. The residue was dissolved in EtOH/H_2O/buffer pH 8 (2:1:1, 1.2 mL) and then diethyl squarate (3.7 μL, 0.0237 mmol) was added. The mixture was stirred for 2 h, concentrated to dryness and then purified by reversed phase chromatography (100% H_2O to 40% MeOH/H_2O) to give the ethyl squaramyl adduct **10** (14 mg, 94% over 2 steps) as a colorless glass. δ_H (500 MHz, D_2O) 1.37–1.65 (15H, m, $OCH_2(CH_2)_6CH_2N$),OCH_2CH_3), 3.52–4.12 (22H, m, 3 × H(2,3,4,5,6a,6b), OCH_2CH_2,CH_2CH_2N), 4.95, 5.06, 5.11 (3H, s, 3 × H1), OCH_2CH_3 is obscured by residual HOD at 4.79 ppm; HRMS (ESI+) *m/z* 778.3104 ($C_{32}H_{53}NNaO_{19}$ $[M + Na]^+$ requires 778.3104). The ^{13}C NMR spectrum for this compound was poorly resolved. We attribute this to the presence of tautomeric isomers.

Crystallography: Intensity data were collected with an Oxford Diffraction Sapphire CCD diffractometer using Cu-Kα radiation (graphite crystal monochromator λ = 1.54184 nm), the temperature during data collection was maintained at 130.0(2) K using an Oxford

Cryosystems cooling device. The structure was solved by direct methods and difference Fourier synthesis. Thermal ellipsoid plot was generated using the program ORTEP-3 [5] integrated within the WINGX [6] suite of programs.

Crystal data for 20: $C_{34}H_{28}O_9$, M = 580.56, T = 130.0(2) K, λ = 1.5418, Orthorhombic, space group $P2_12_12_1$ a = 9.5130(2), b = 15.8165(2), c =19.1797(3) Å, V= 2885.82(8) Å^3, Z = 4, D_c = 1.336 mg M^{-3} μ(Cu-Kα) 0.805 mm^{-1}, F(000) = 1216, crystal size 0.4 × 0.3 × 0.25 mm. 9744 reflections measured, 4808 independent reflections (R_{int} = 0.0255) the final R was 0.0345 [I > 2σ(I)] and wR(F^2) was 0.0982 (all data).

References

1. Pangborn, A. B.; Giardello, M. A.; Grubbs, R. H.; Rosen, R. K.; Timmers, F. J. *Organometallics* **1996,** *15,* 1518–1520. doi:10.1021/om9503712
2. Still, W. C.; Kahn, M.; Mitra, A. M. *J. Org. Chem.* **1978,** *43,* 2923–2925. doi:10.1021/jo00408a041
3. Heng, L.; Ning, J.; Kong, F. *J. Carbohydr. Chem.* **2001,** *20,* 285–296. doi:10.1081/CAR-100104864
4. Tam, P.-H.; Lowary, T. L. *Carbohydr. Res.* **2007,** *342,* 1741–1772. doi:10.1016/j.carres.2007.05.001
5. Farrugia, L. J. *J. Appl. Crystallogr.* **1997,** *30,* 565. doi:10.1107/S0021889897003117
6. Farrugia, L. J. *J. Appl. Crystallogr.* **1999,** *32,* 837–838. doi:10.1107/S0021889899006020
