# Supplementary material for: Synthesis of glycoconjugate fragments of mycobacterial phosphatidylinositol mannosides and lipomannan
Source: Beilstein J Org Chem. 2011 Mar 28;7:369–77. doi: 10.3762/bjoc.7.47 (PMC3079179; doi:10.3762/bjoc.7.47)
Supplement: File 2 — 1H and 13C NMR spectra for new compounds and fluorescence spectra for 5–7. The crystallographic data file for the structure reported in this paper has been deposited with the Cambridge Crystallographic Data Centre as file CCDC 804936 and is available on request from http://www.ccdc.cam.ac.uk/. [file Beilstein_J_Org_Chem-07-369-s002.pdf]

Supporting Information File 2

for

Synthesis of glycoconjugate fragments of mycobacterial phosphatidylinositol mannosides and lipomannan

Benjamin Cao, Jonathan M. White and Spencer J. Williams*

Address: School of Chemistry and Bio21 Molecular Science and Biotechnology Institute, University of Melbourne, Parkville, Victoria, Australia, Fax: +61 3 9347 8124; Tel: +61 3 8344 2422

E-mail: Spencer J. Williams* - sjwill@unimelb.edu.au

* Corresponding author

^{1}H and ^{13}C NMR spectra for new compounds and fluorescence spectra for 5–7

Table of contents

Azidooctyl α-D-mannopyranoside (12) **........4**

8-Azidooctyl 6-O-(tert-butyldiphenylsilyl)-α-D-mannopyranoside **........5**

8-Azidooctyl 2,3,4-tri-O-benzoyl-6-O-(tert-butyldiphenylsilyl)-α-D-mannopyranoside (13) **........6**

8-Azidooctyl 2,3,4-tri-O-benzoyl-α-D-mannopyranoside (14) **........7**

3,4,6-Tri-O-benzoyl-1,2-O-(S)-benzylidene-β-D-mannopyranose (18) **........8**

4-Methylphenyl 2-O-acetyl-3,4,6-tri-O-benzoyl-1-thio-α-D-mannopyranoside (17) **........9**

8-Azidooctyl 3,4-di-O-(2,3-dimethoxybutane-2,3-diyl)-α-D-mannopyranoside (15)..................**10**

8-Azidooctyl (2,3,4,6-tetra-O-benzoyl-α-D-mannopyranosyl)-(1→6)-2,3,4-tri-O-benzoyl-α-D-mannopyranoside (20)..................**11**

8-Azidooctyl (α-D-mannopyranosyl)-(1→6)-α-D-mannopyranoside (1)..................**12**

8-Azidooctyl (2,3,4-tri-O-benzoyl-6-O-(tert-butyldiphenylsilyl)-α-D-mannopyranosyl)-(1→6)-2,3,4-tri-O-benzoyl-α-D-mannopyranoside (23)..................**13**

8-Azidooctyl (2,3,4-tri-O-benzoyl-α-D-mannopyranosyl)-(1→6)-2,3,4-tri-O-benzoyl-α-D-mannopyranoside (24)..................**14**

8-Azidooctyl (2,3,4,6-tetra-O-benzoyl-α-D-mannopyranosyl)-(1→6)-(2,3,4-tri-O-benzoyl-α-D-mannopyranosyl)-(1→6)-2,3,4-tri-O-benzoyl-α-D-mannopyranoside (25)..................**15**

8-Azidooctyl (α-D-mannopyranosyl)-(1→6)-(α-D-mannopyranosyl)-(1→6)-α-D-mannopyranoside (2)..................**16**

8-Azidooctyl (2-O-acetyl-3,4,6-tri-O-benzoyl-α-D-mannopyranosyl)-(1→6)-2,3,4-tri-O-benzoyl-α-D-mannopyranoside (26)..................**17**

8-Azidooctyl (3,4,6-tri-O-benzoyl-α-D-mannopyranosyl)-(1→6)-2,3,4-tri-O-benzoyl-α-D-mannopyranoside (27)..................**18**

8-Azidooctyl (2,3,4-tri-O-benzoyl-α-D-mannopyranosyl)-(1→2)-(3,4,6-tri-O-benzoyl-α-D-mannopyranosyl)-(1→6)-2,3,4,6-tetra-O-benzoyl-α-D-mannopyranoside (28)..................**19**

8-Azidooctyl (α-D-mannopyranosyl)-(1→2)-(α-D-mannopyranosyl)-(1→6)-α-D-mannopyranoside (3)..................**20**

Azidooctyl 2,6-di-O-(2,3,4,6-tetra-O-benzoyl-α-D-mannopyranosyl)-3,4-di-O-(2,3-dimethoxybutane-2,3-diyl)-α-D-mannopyranoside (29)..................**21**

Azidooctyl 2,6-di-O-α-D-mannopyranosyl-α-D-mannopyranoside (4)..................**22**

4-[(7-Nitrobenzofurazan-4-yl)aminomethyl]-triazol-1-yloctyl (α-D-mannopyranosyl)-(1→6)-α-D-mannopyranoside (5)..................**23**

4-[(7-Nitrobenzofurazan-4-yl)aminomethyl]-triazol-1-yloctyl (α-D-mannopyranosyl)-(1→6)-(α-D-mannopyranosyl)-(1→6)-α-D-mannopyranoside (6)..................**24**

4-[(7-Nitrobenzofurazan-4-yl)aminomethyl]-triazol-1-yloctyl (α-D-mannopyranosyl)-(1→2)-(α-D-mannopyranosyl)-(1→6)-α-D-mannopyranoside (7)..................**25**

8-(2-Ethoxycylobutene-3,4-dione-1-ylamino)octyl (α-D-mannopyranosyl)-(1→6)-(α-D-mannopyranosyl)-(1→6)-α-D-mannopyranoside (8)..................**26**

8-(2-Ethoxycylobutene-3,4-dione-1-ylamino)octyl (α-D-mannopyranosyl)-(1→2)-(α-D-mannopyranosyl)-(1→6)-α-D-mannopyranoside (9)..................**27**

***8-(2-Ethoxycylobutene-3,4-dione-1-ylamino)octyl 2,6-di-O-α-D-mannopyranosyl-α-D-mannopyranoside (10)*..28**

***Figure 1: Fluorescence emission spectra of the NBD-labelled neoglycoconjugates 5–7.*........29**

***Figure 2: 125 MHz ^{1}H coupled ^{13}C spectra of trisaccharides 25, 28 and 29. $^{1}J_{C,H}$ values of anomeric carbons are indicated.*...30**

Azidooctyl α-D-mannopyranoside (12)

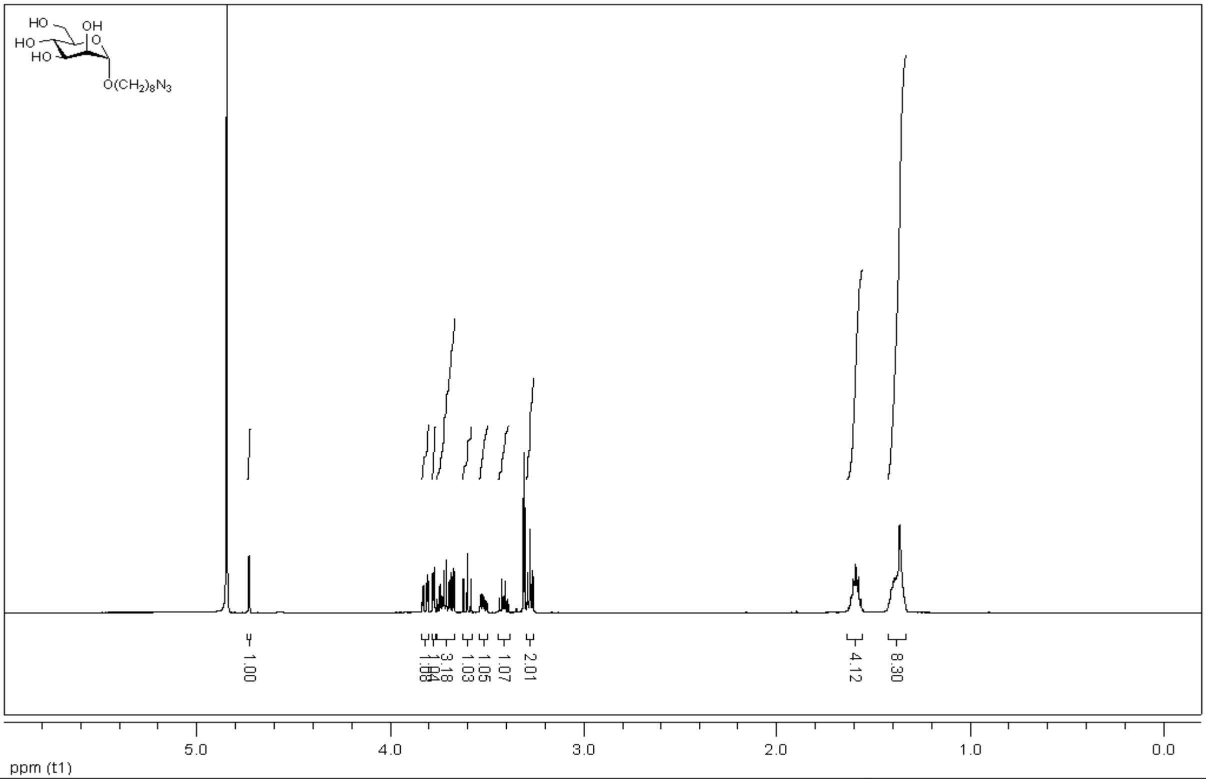

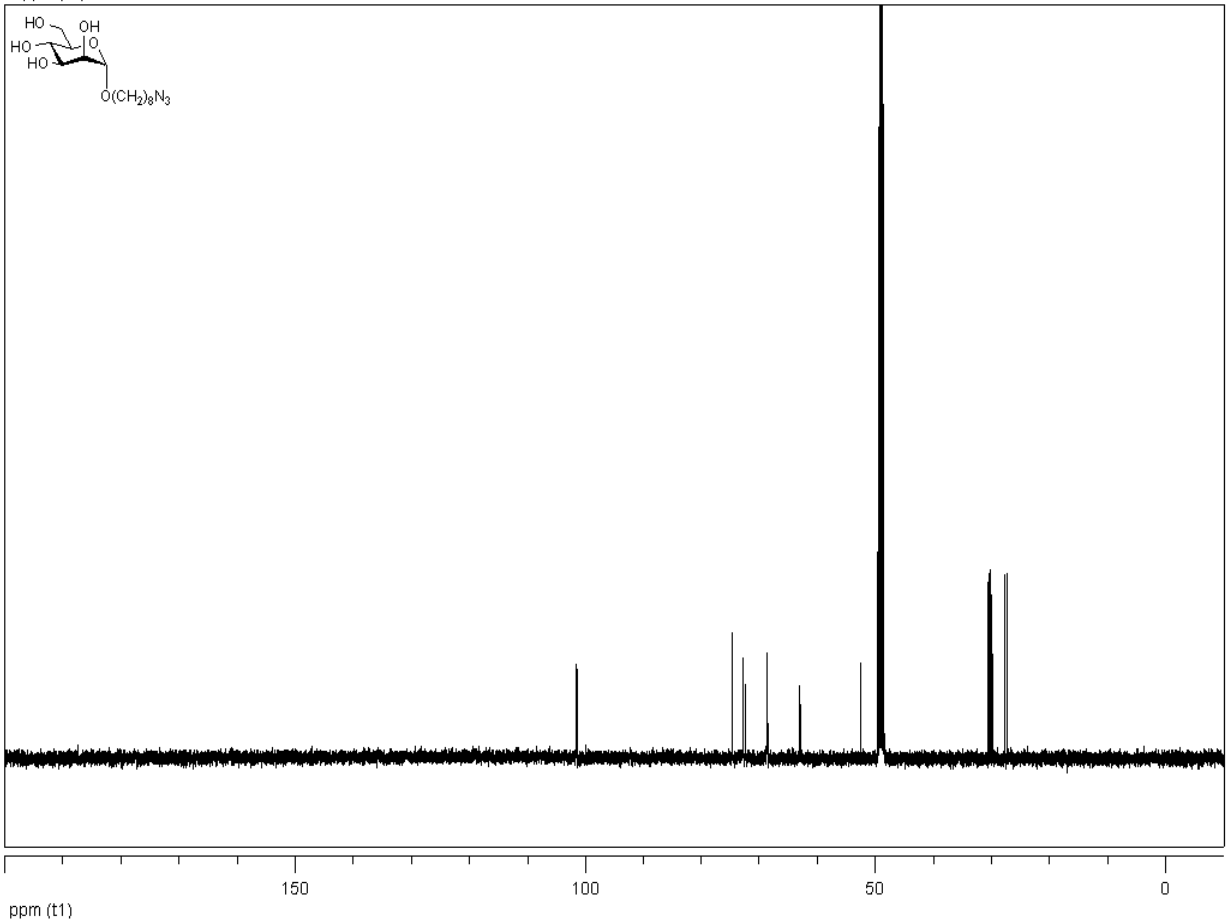

8-Azidooctyl 6-*O*-(*tert*-butyldiphenylsilyl)-α-D-mannopyranoside

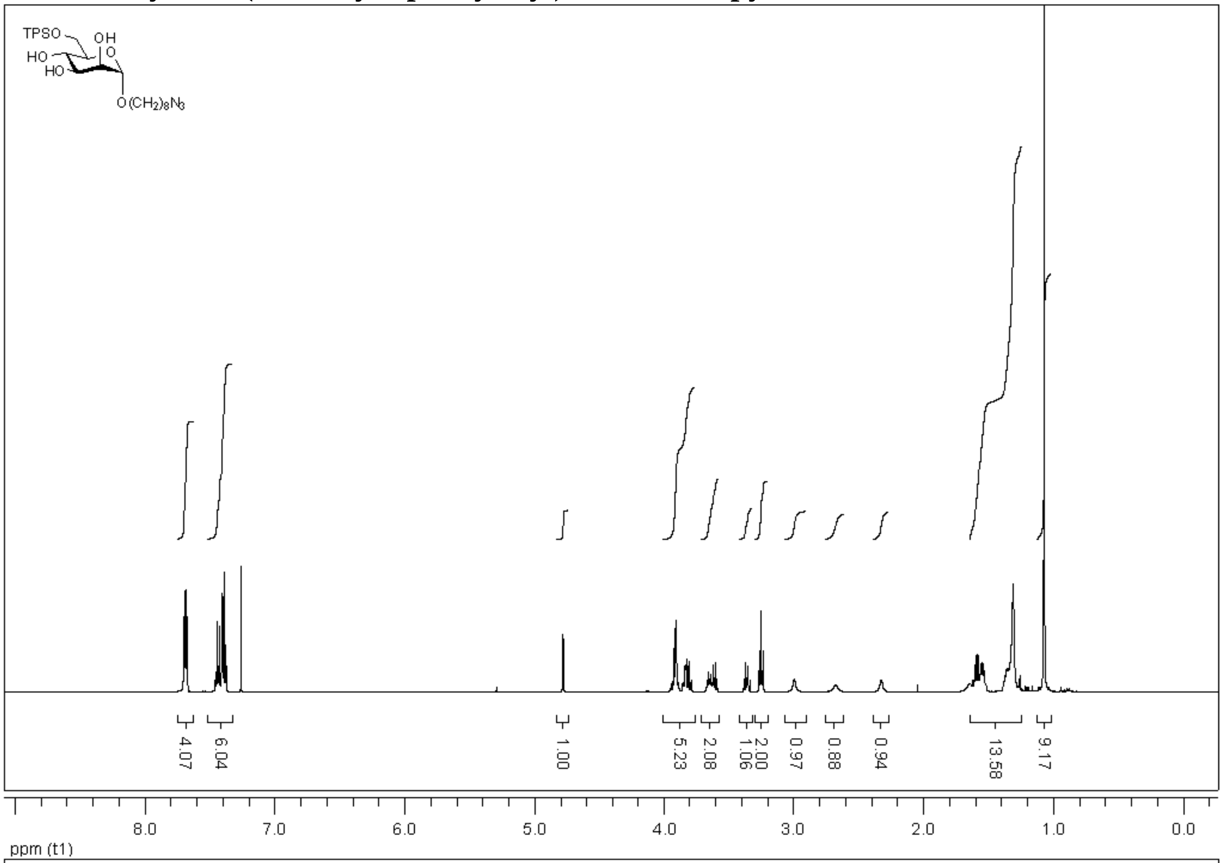

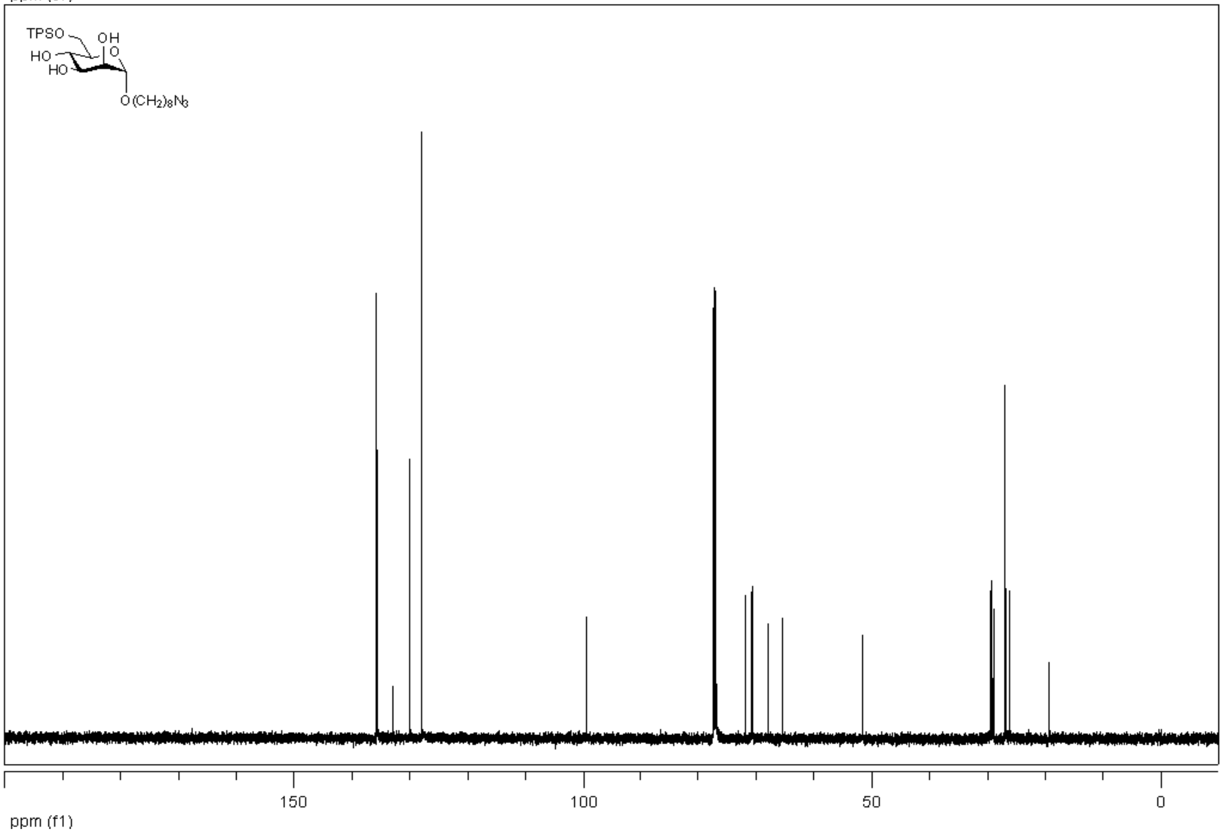

8-Azidooctyl 2,3,4-tri-*O*-benzoyl-6-*O*-(*tert*-butyldiphenylsilyl)-α-D-mannopyranoside (13)

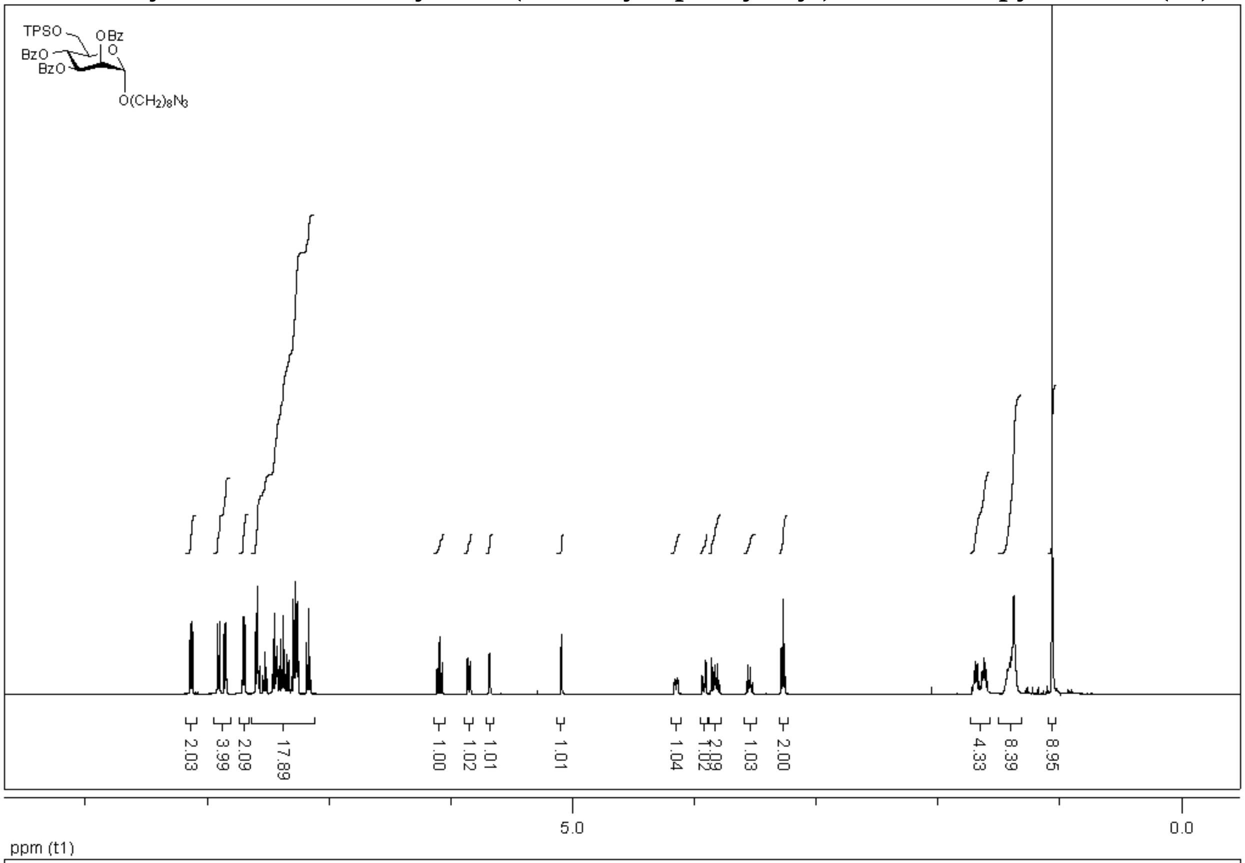

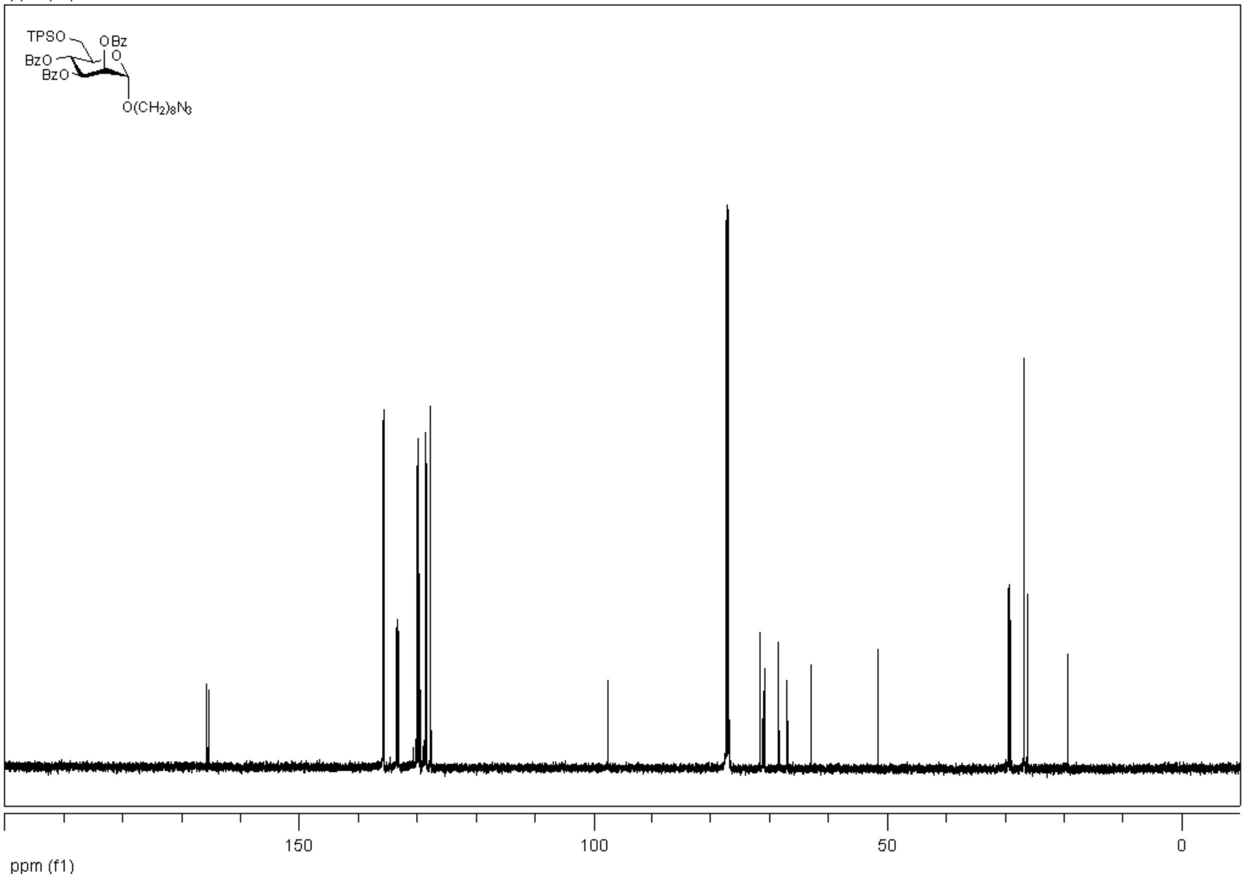

8-Azidooctyl 2,3,4-tri-*O*-benzoyl-α-D-mannopyranoside (14)

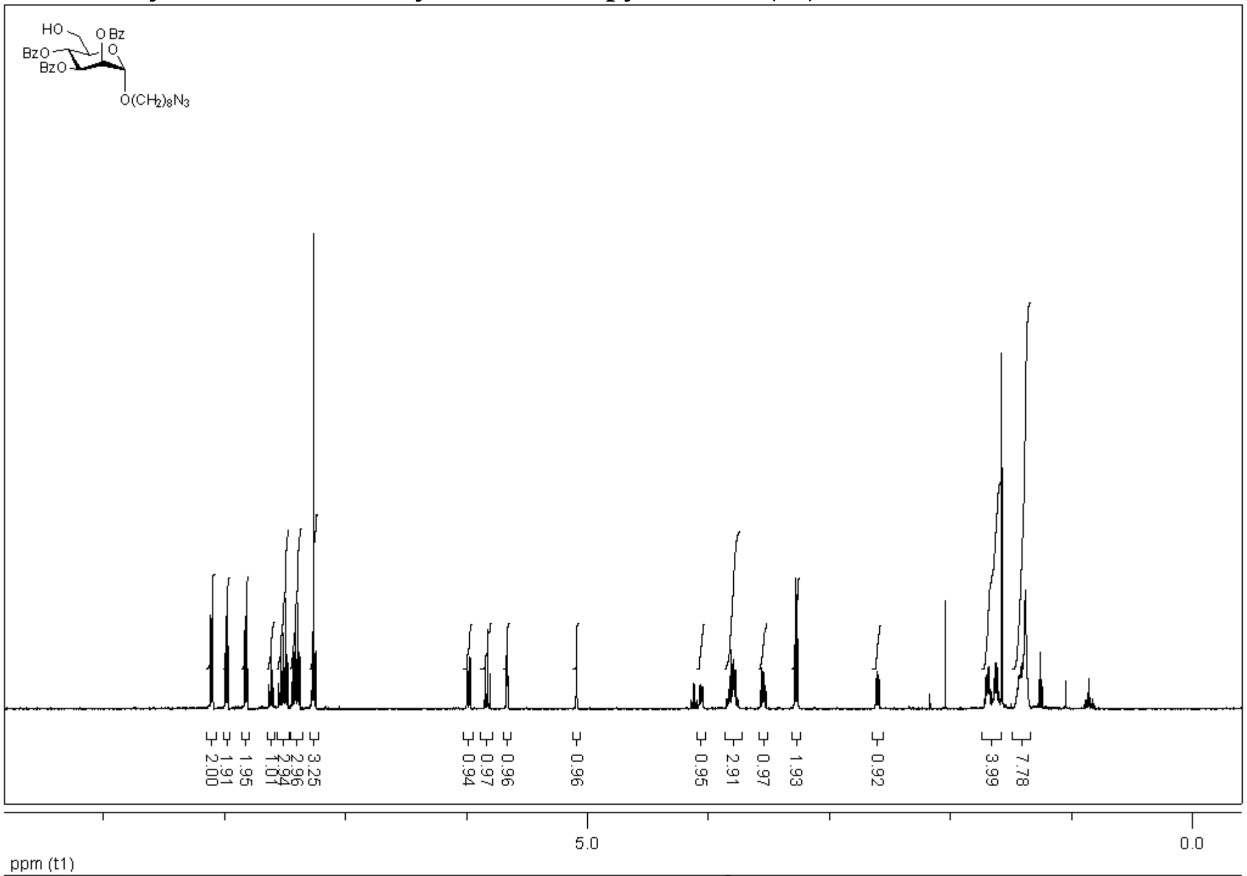

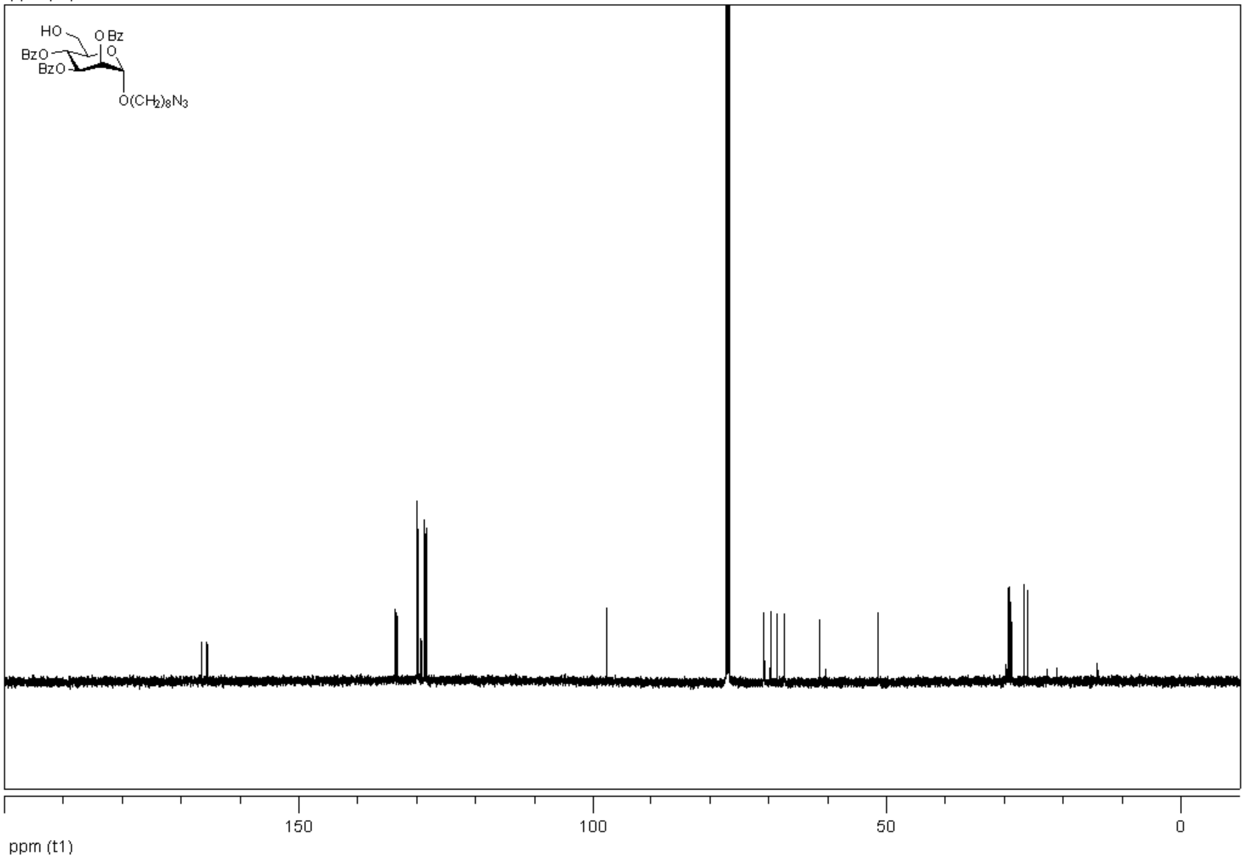

3,4,6-Tri-*O*-benzoyl-1,2-*O*-(*S*)-benzylidene-β-D-mannopyranose (18)

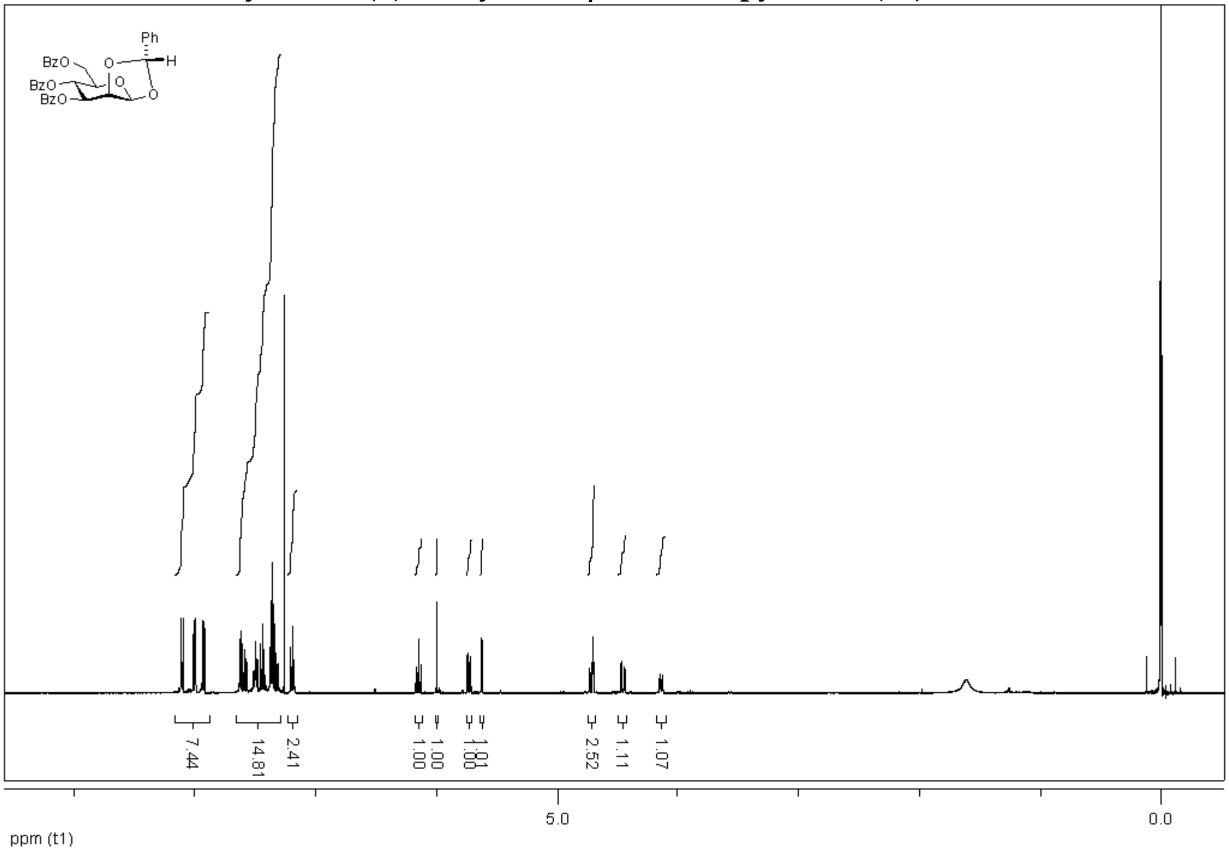

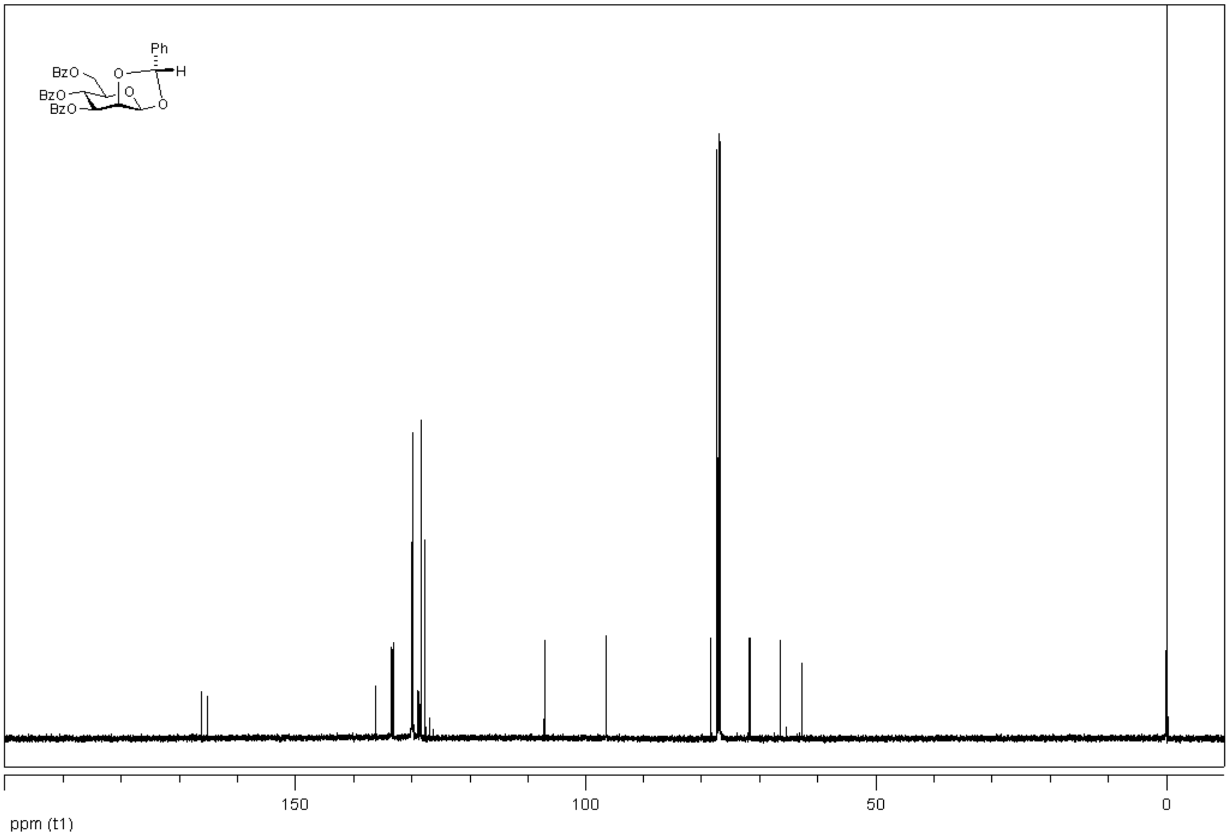

4-Methylphenyl 2-*O*-acetyl-3,4,6-tri-*O*-benzoyl-1-thio-α-D-mannopyranoside (17)

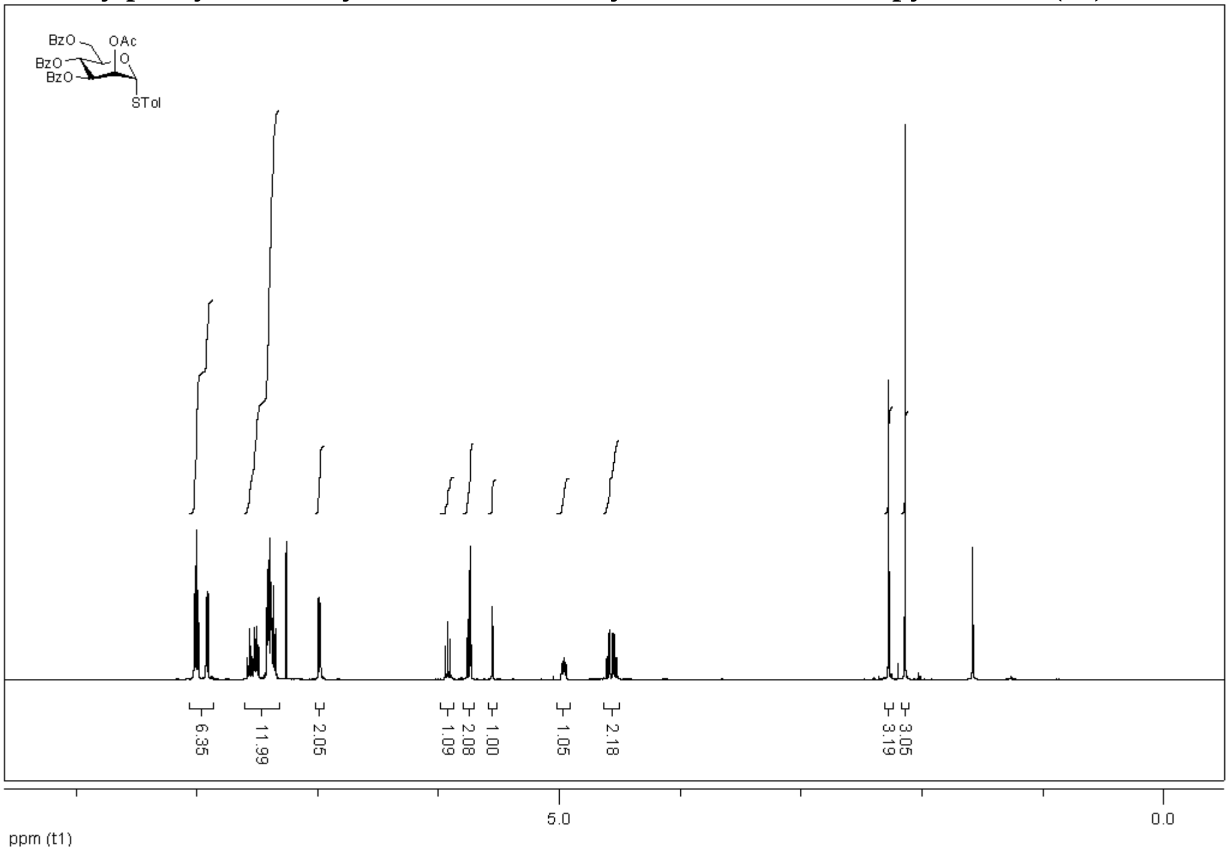

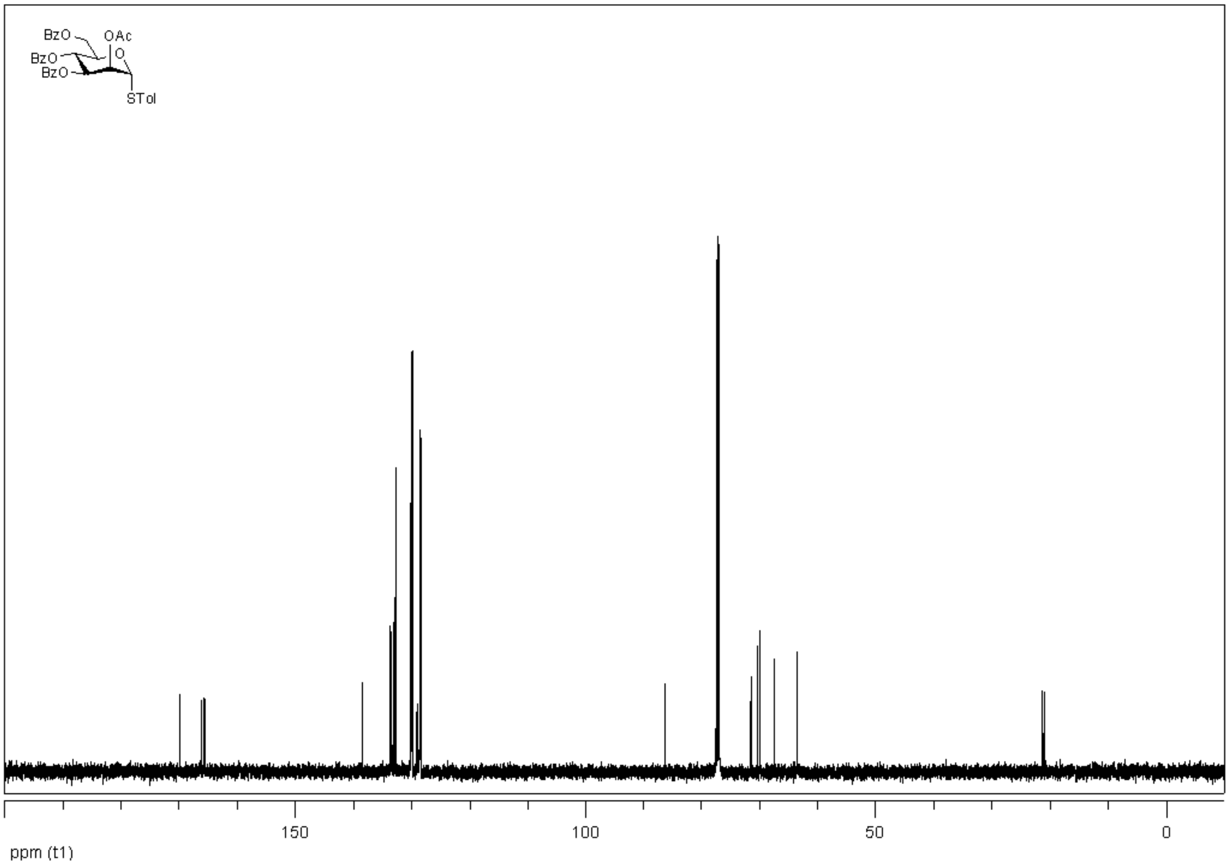

8-Azidooctyl 3,4-di-*O*-(2,3-dimethoxybutane-2,3-diyl)-α-D-mannopyranoside (15)

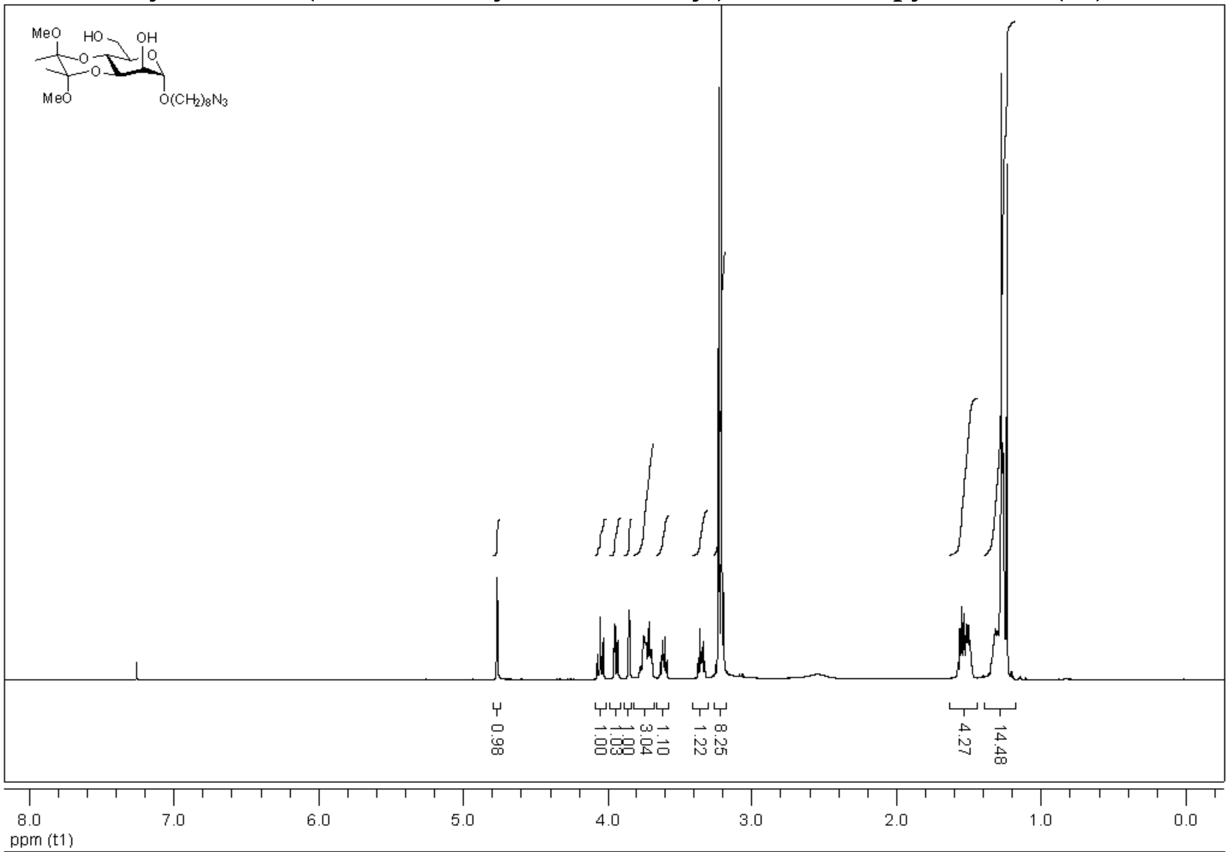

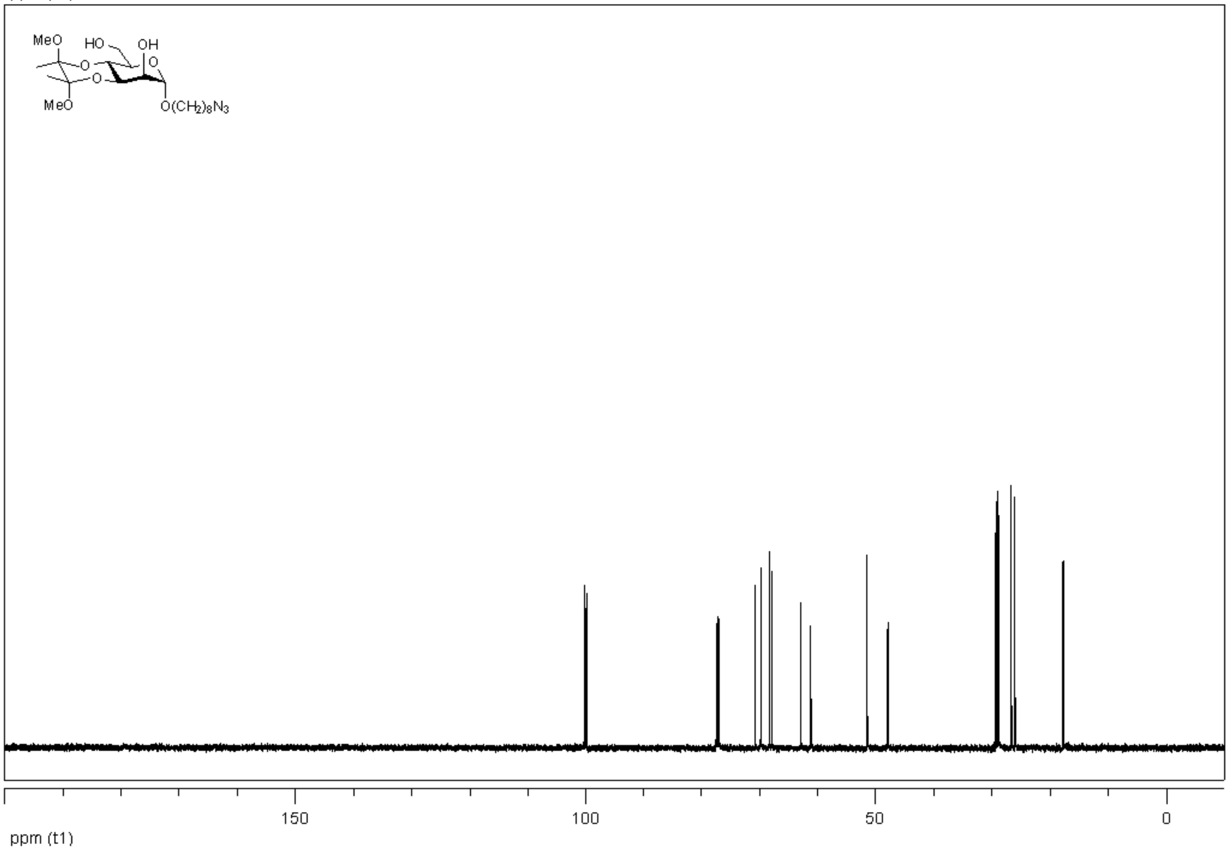

8-Azidooctyl (2,3,4,6-tetra-*O*-benzoyl-α-D-mannopyranosyl)-(1→6)-2,3,4-tri-*O*-benzoyl-α-D-mannopyranoside (20)

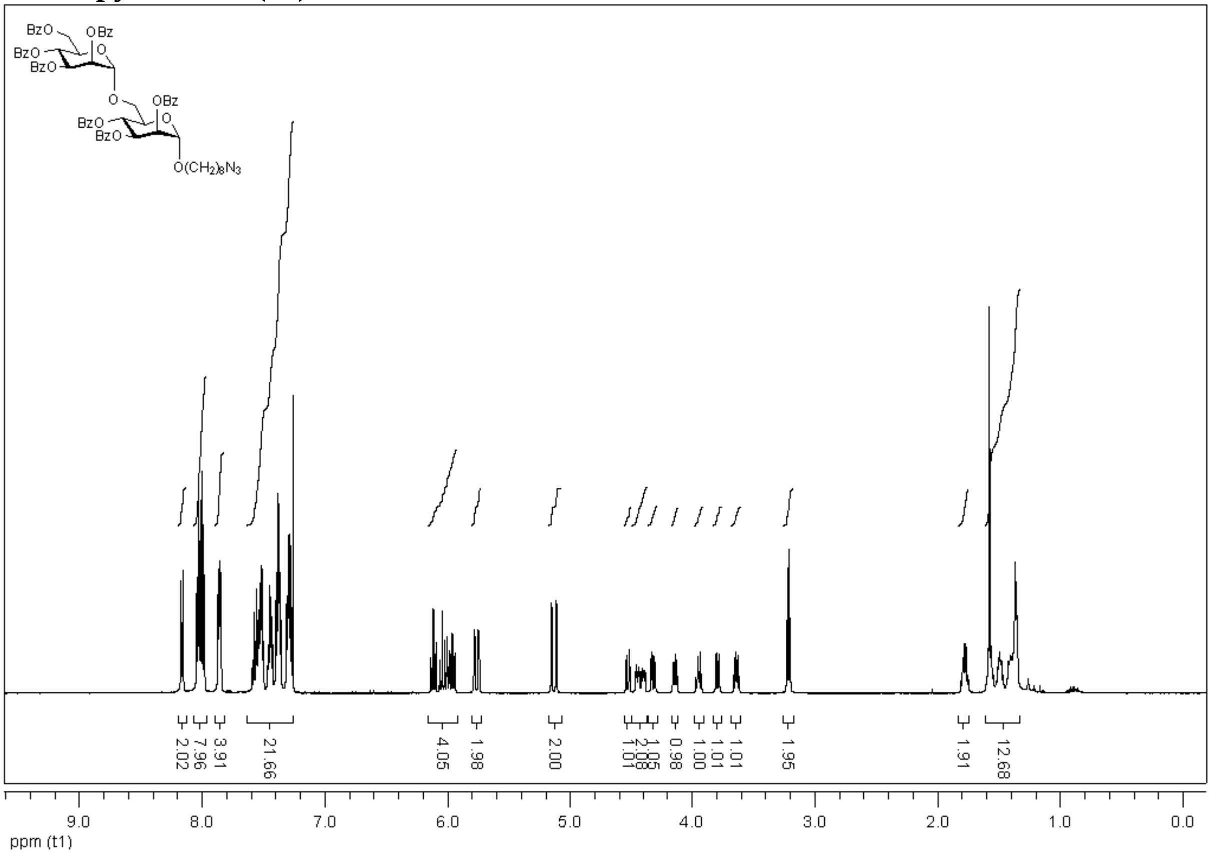

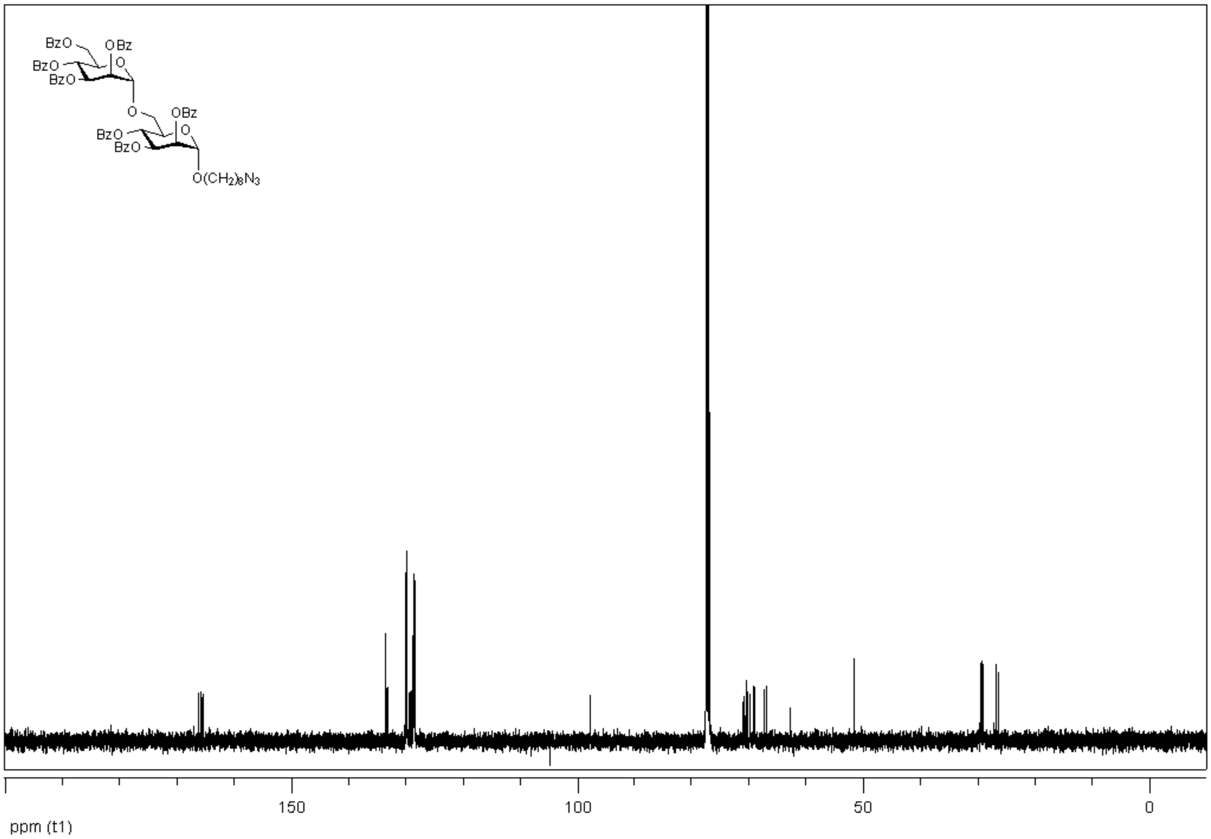

8-Azidooctyl (α-D-mannopyranosyl)-(1→6)-α-D-mannopyranoside (1)

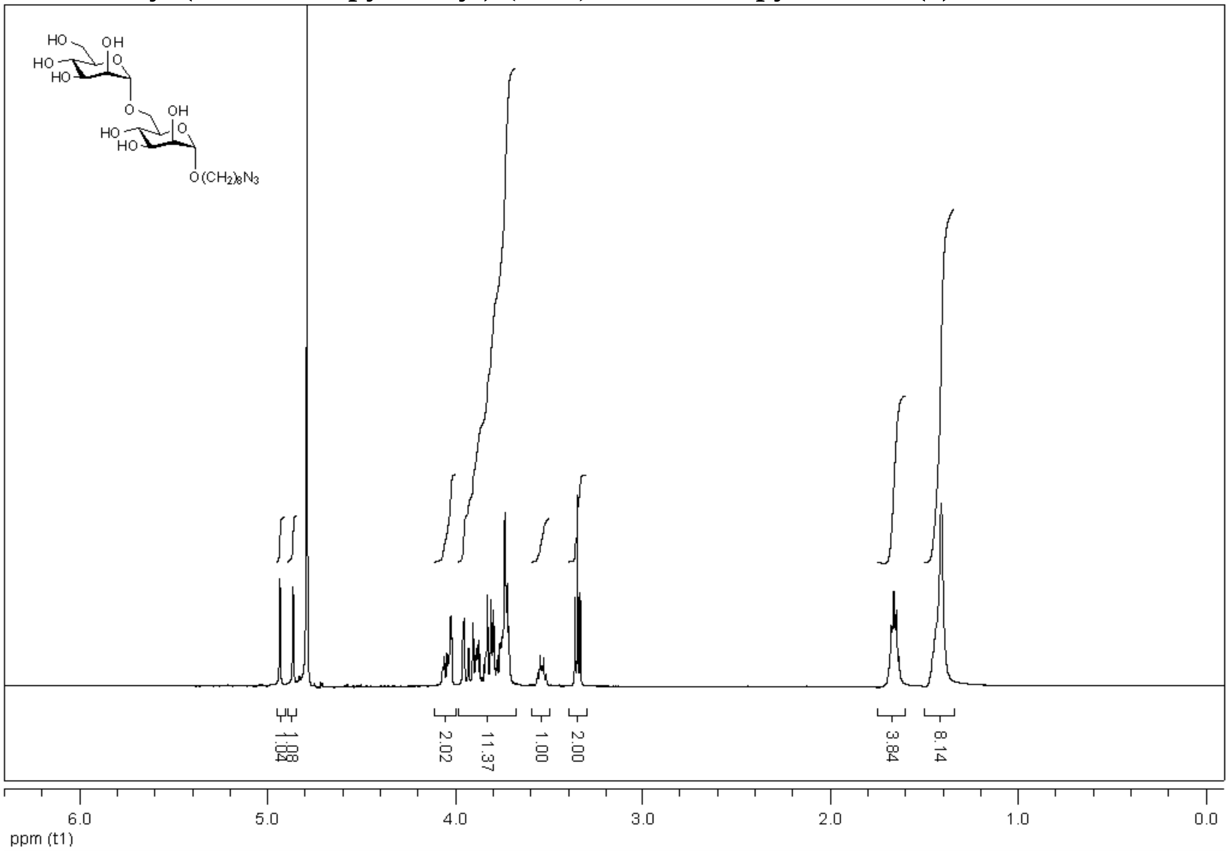

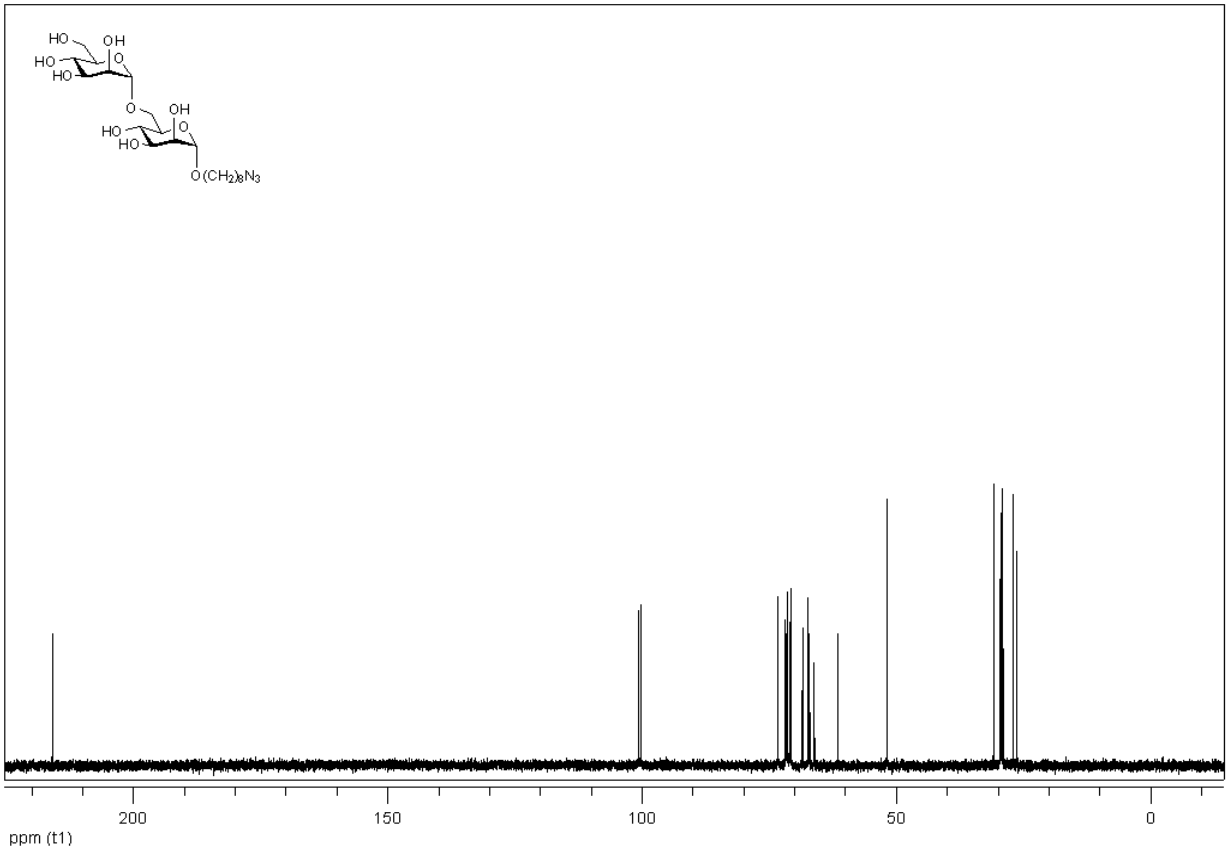

8-Azidooctyl (2,3,4-tri-*O*-benzoyl-6-*O*-(*tert*-butyldiphenylsilyl)-α-D-mannopyranosyl)-(1→6)-2,3,4-tri-*O*-benzoyl-α-D-mannopyranoside (23)

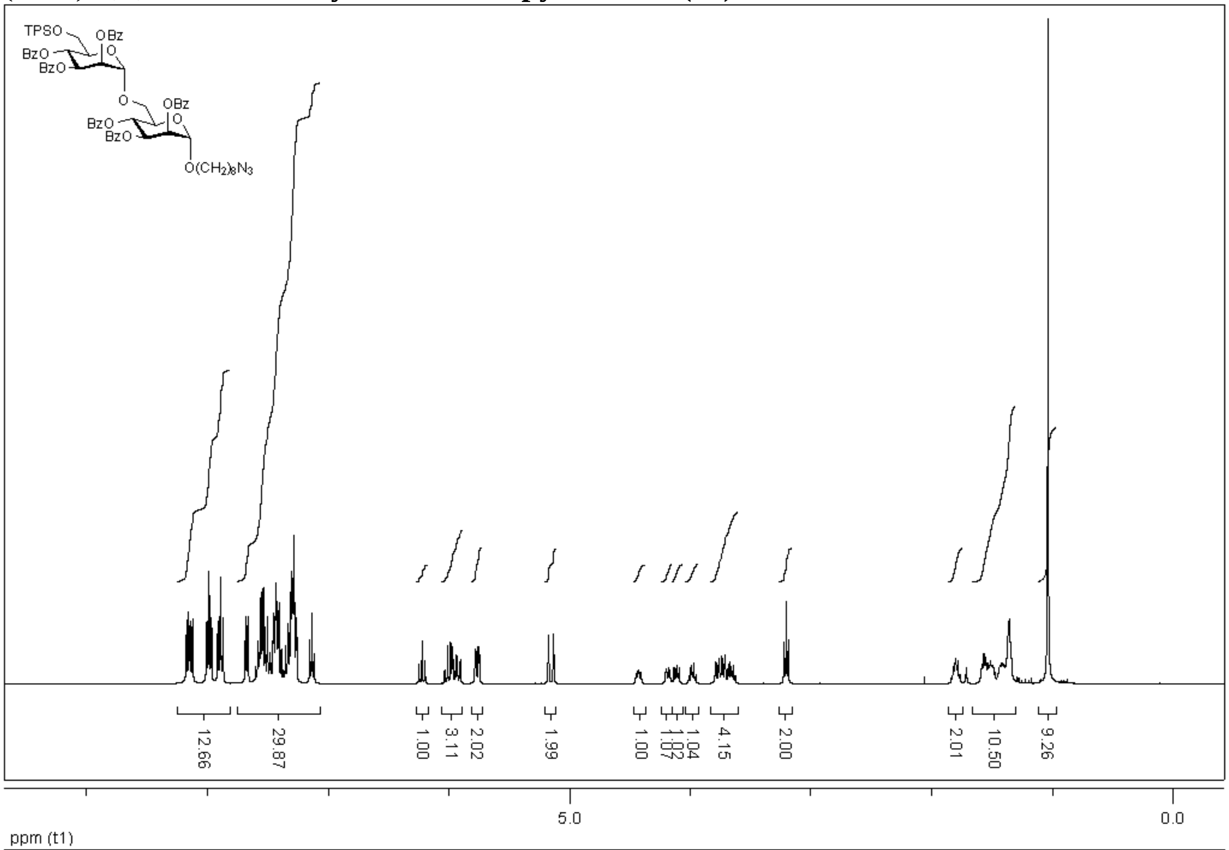

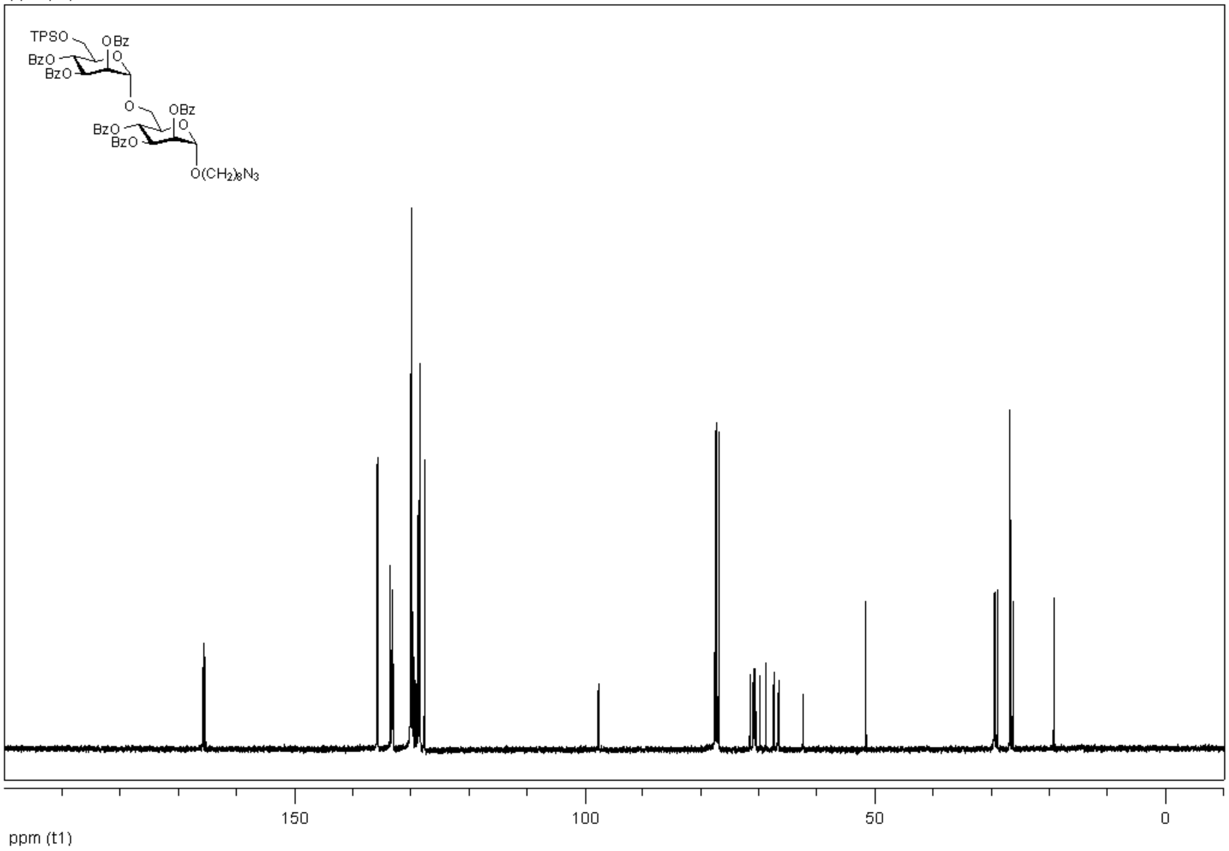

8-Azidooctyl (2,3,4-tri-*O*-benzoyl-α-D-mannopyranosyl)-(1→6)-2,3,4-tri-*O*-benzoyl-α-D-mannopyranoside (24)

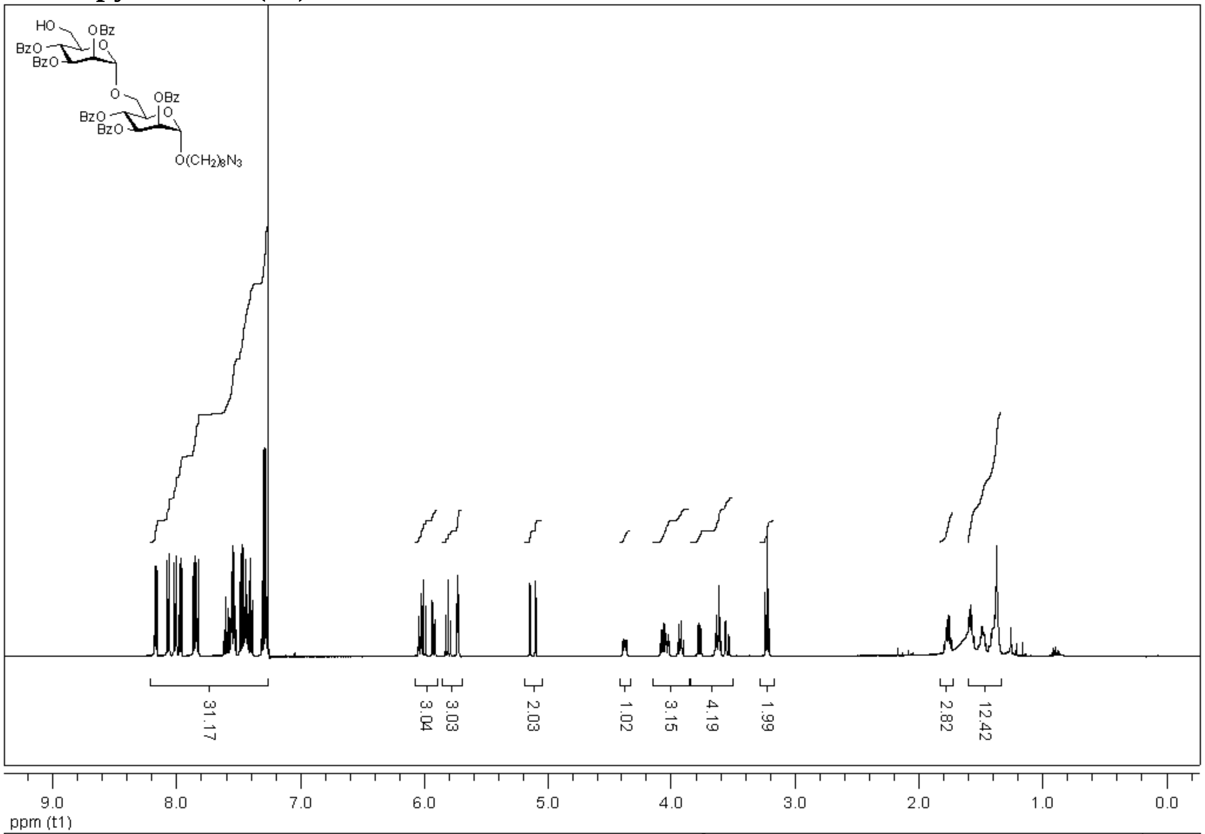

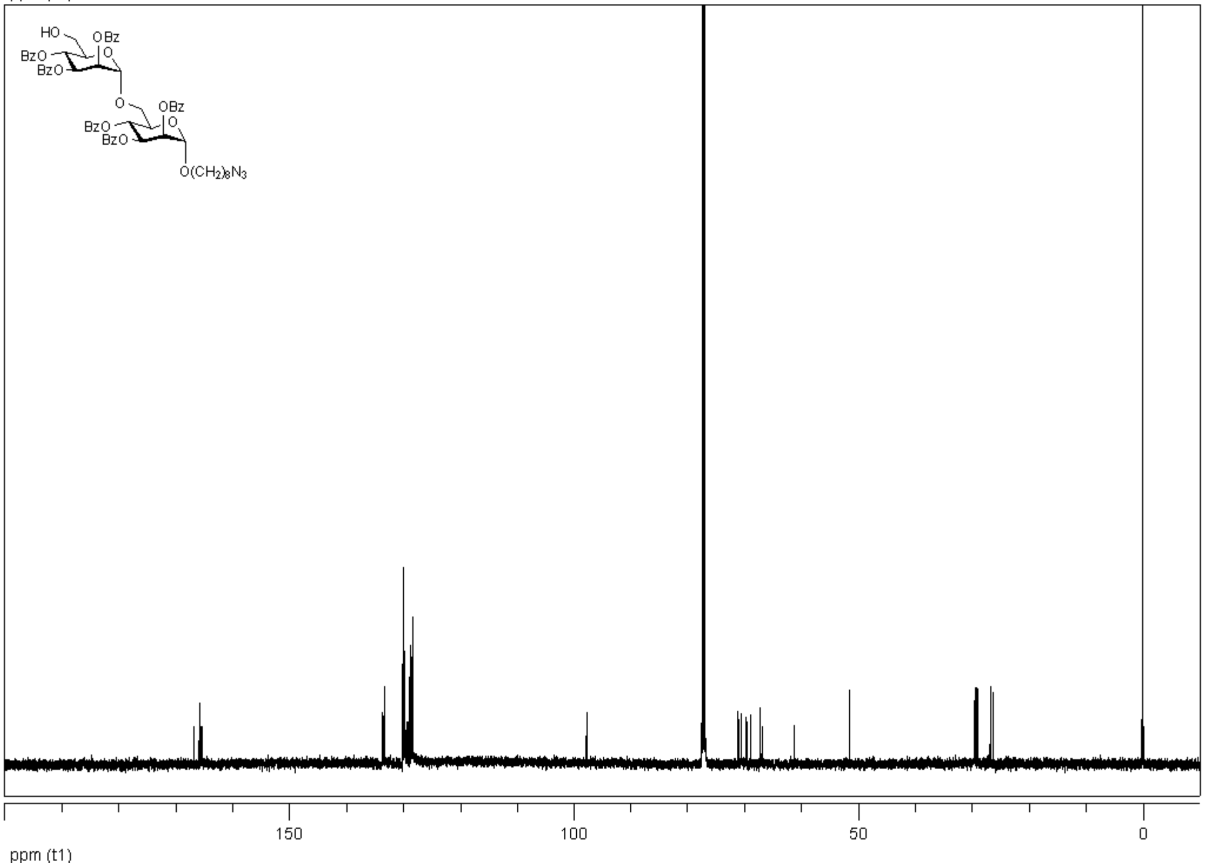

8-Azidooctyl (2,3,4,6-tetra-*O*-benzoyl-α-D-mannopyranosyl)-(1→6)-(2,3,4-tri-*O*-benzoyl-α-D-mannopyranosyl)-(1→6)-2,3,4-tri-*O*-benzoyl-α-D-mannopyranoside (25)

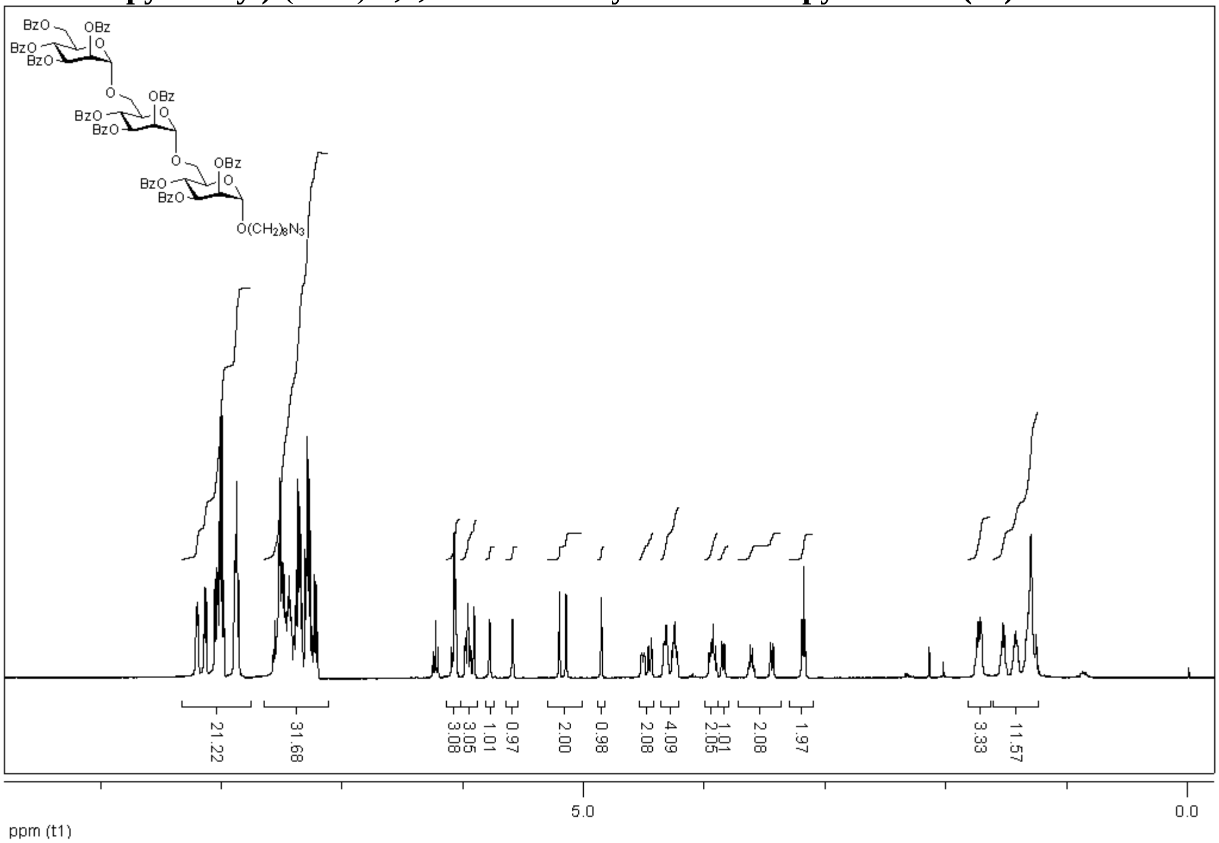

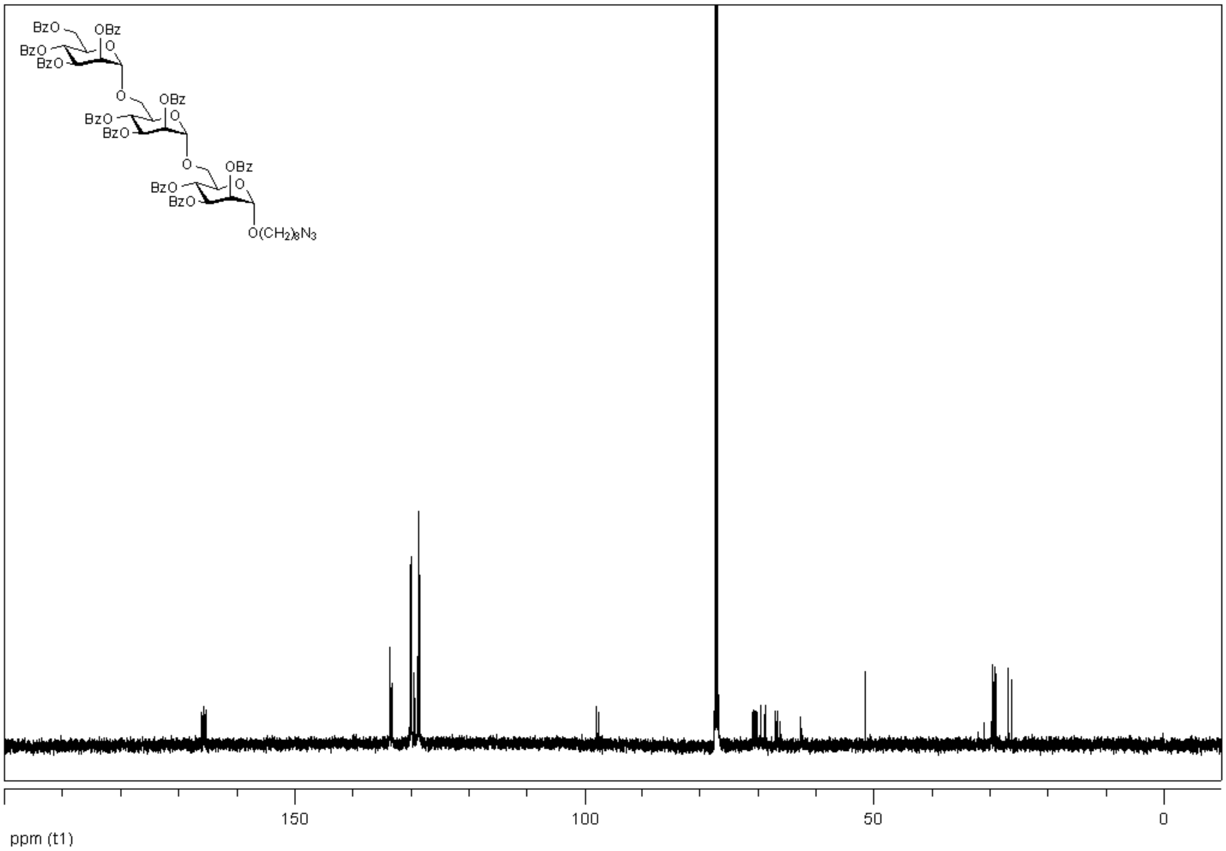

8-Azidooctyl (α-D-mannopyranosyl)-(1→6)-(α-D-mannopyranosyl)-(1→6)-α-D-mannopyranoside (2)

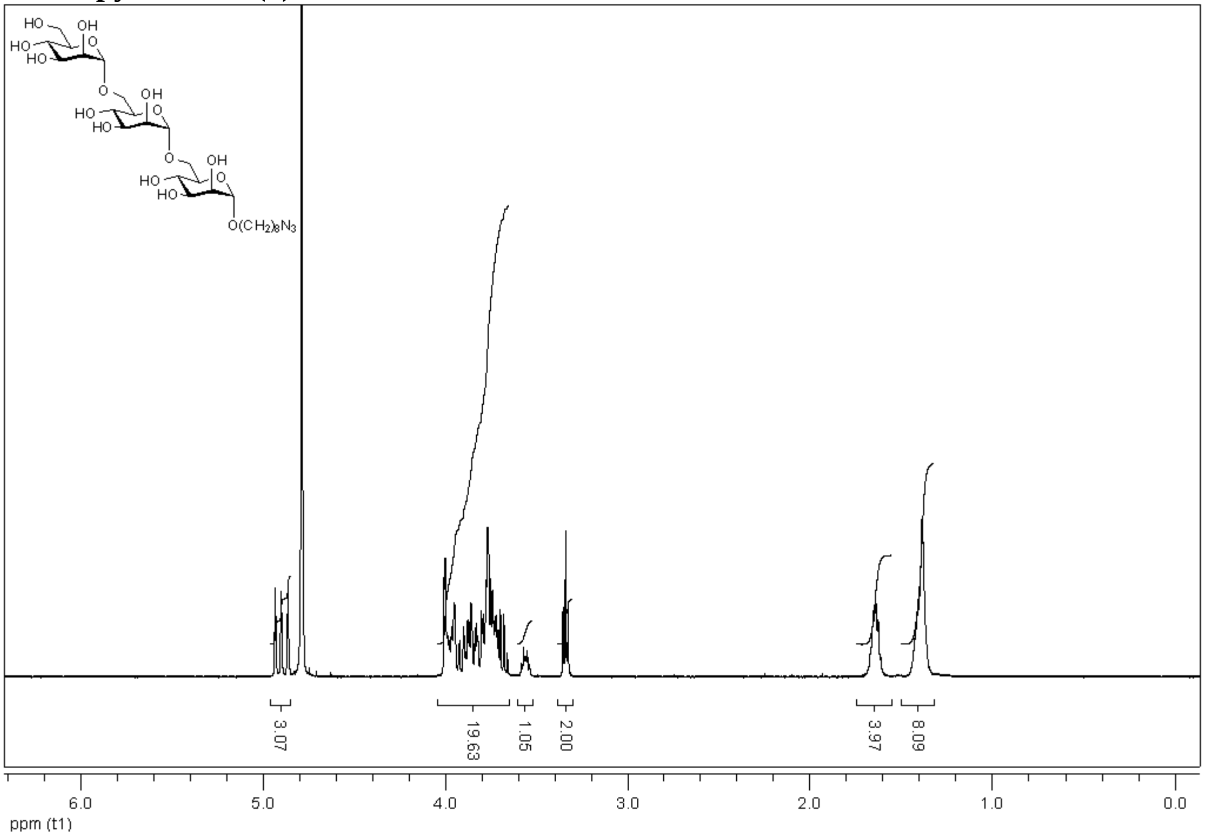

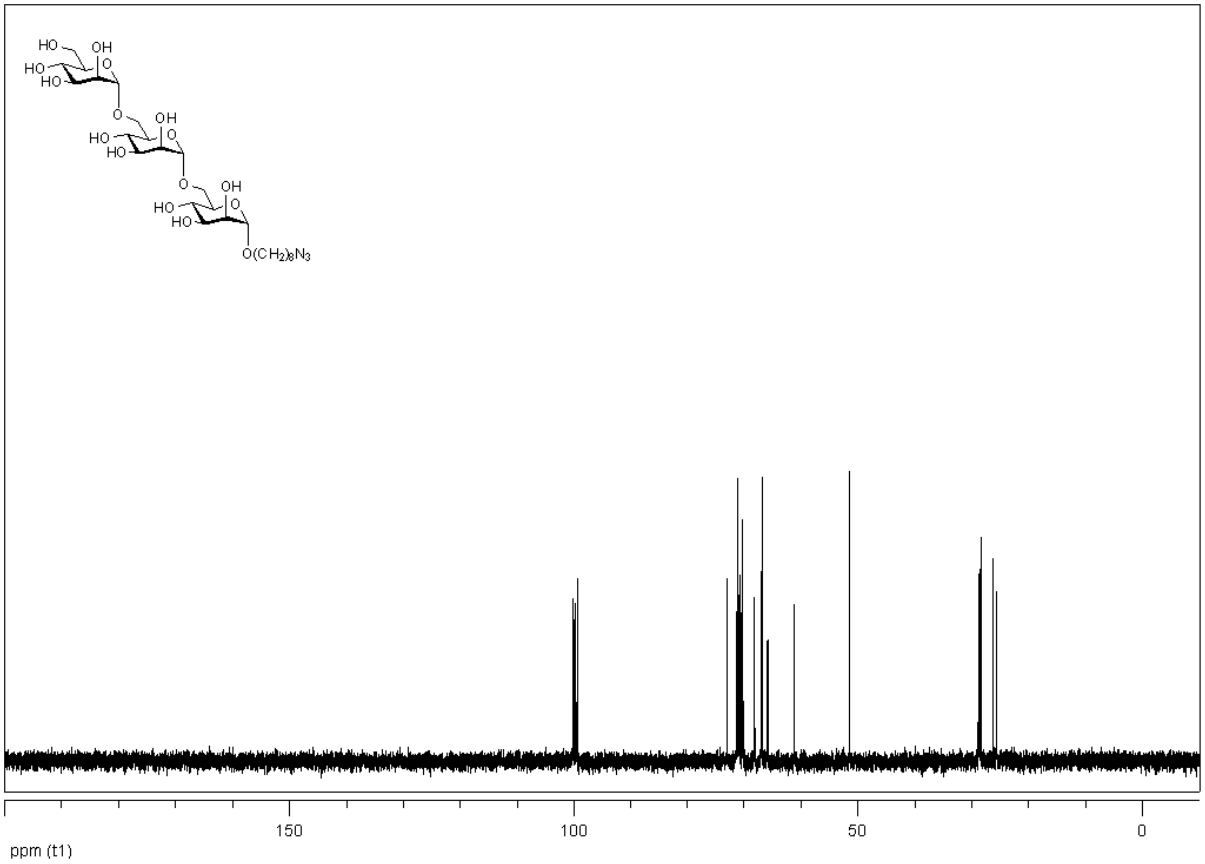

8-Azidooctyl (2-*O*-acetyl-3,4,6-tri-*O*-benzoyl-α-D-mannopyranosyl)-(1→6)-2,3,4-tri-*O*-benzoyl-α-D-mannopyranoside (26)

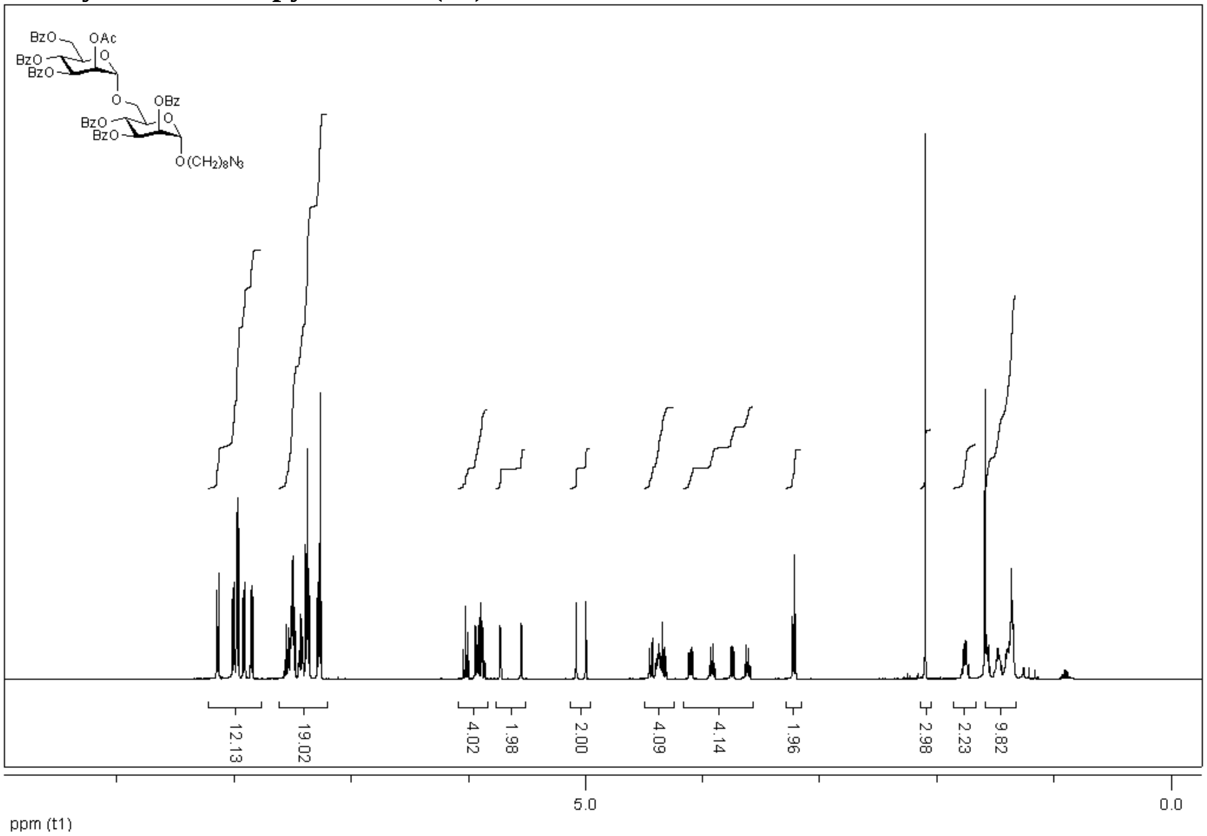

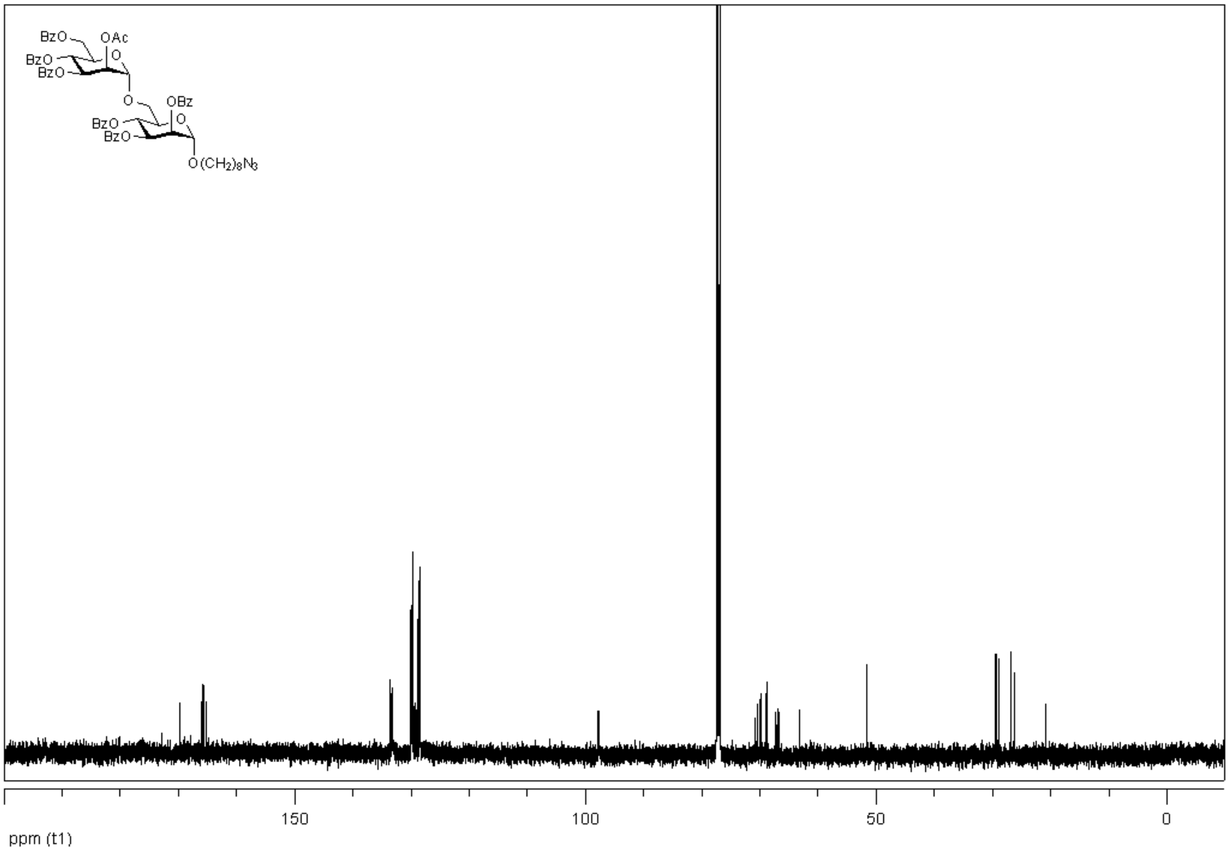

8-Azidooctyl (3,4,6-tri-*O*-benzoyl-α-D-mannopyranosyl)-(1→6)-2,3,4-tri-*O*-benzoyl-α-D-mannopyranoside (27)

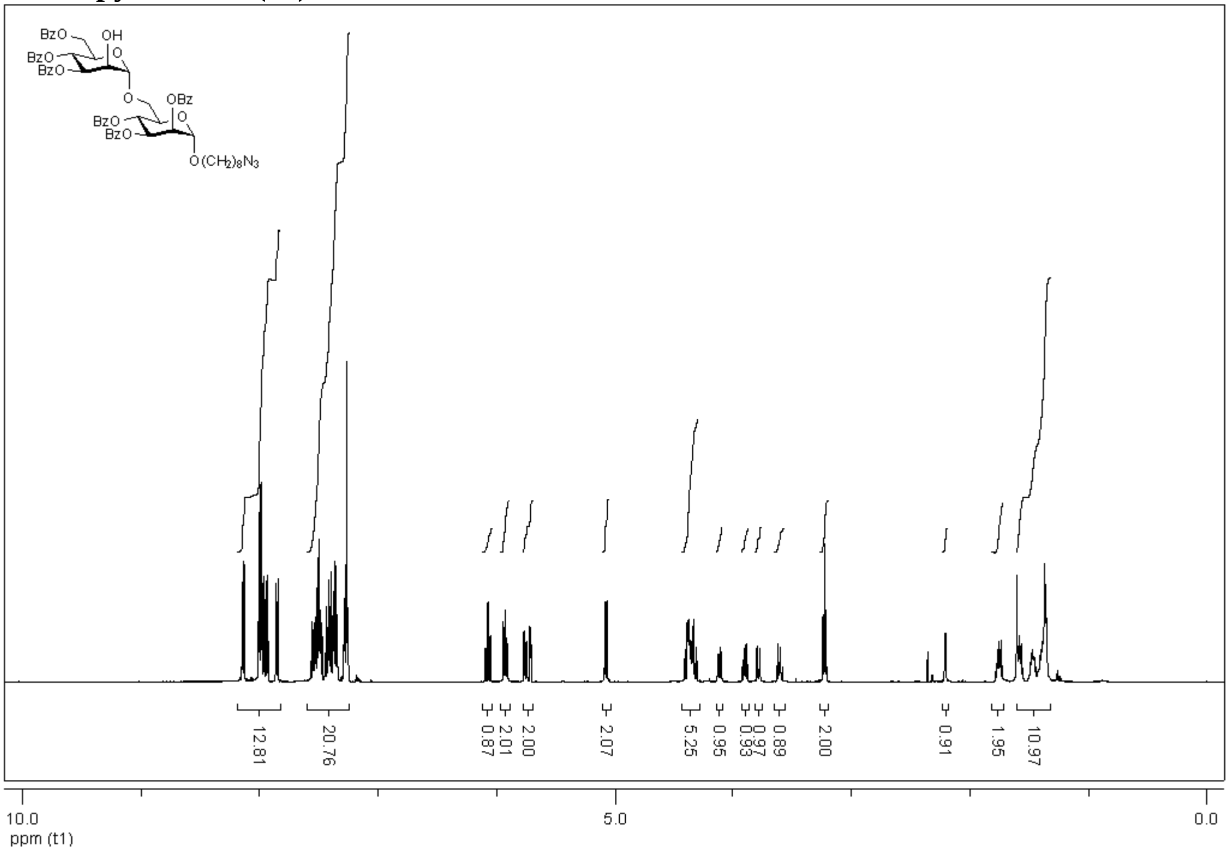

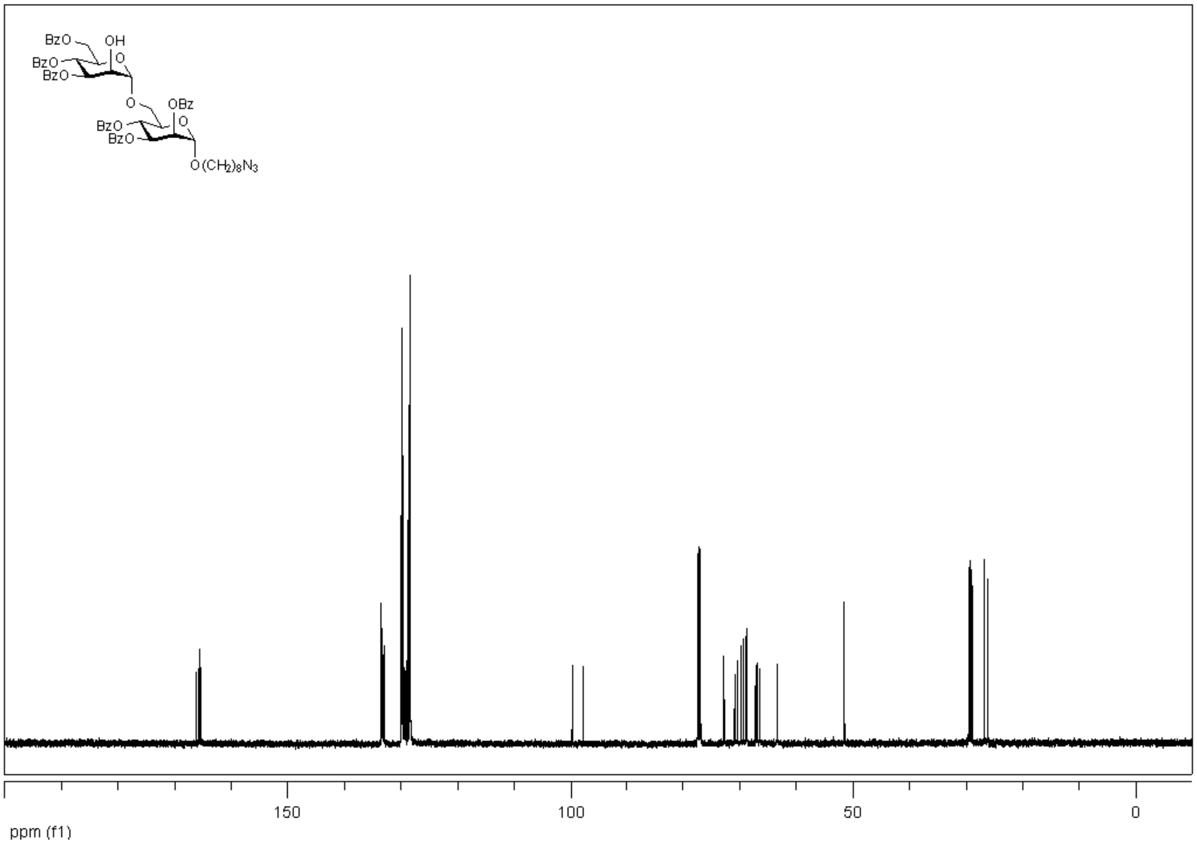

8-Azidooctyl (2,3,4-tri-*O*-benzoyl-α-D-mannopyranosyl)-(1→2)-(3,4,6-tri-*O*-benzoyl-α-D-mannopyranosyl)-(1→6)-2,3,4,6-tetra-*O*-benzoyl-α-D-mannopyranoside (28)

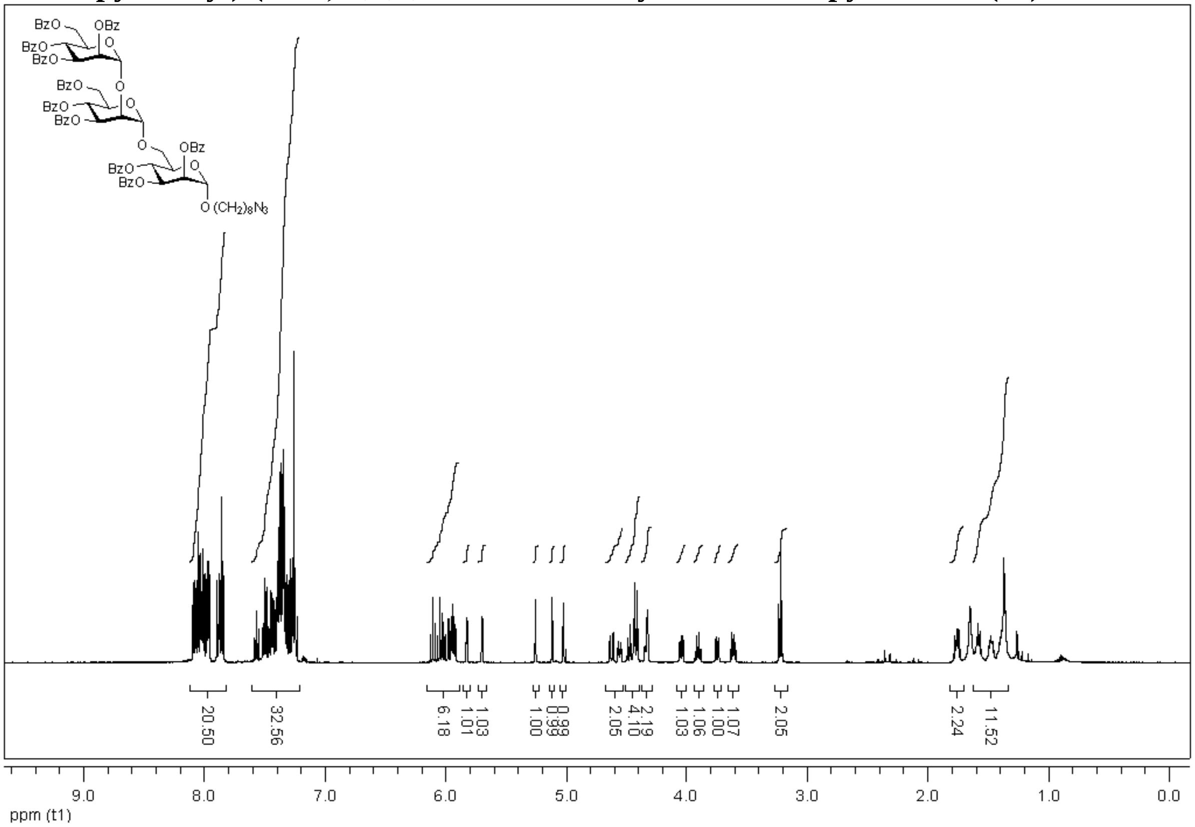

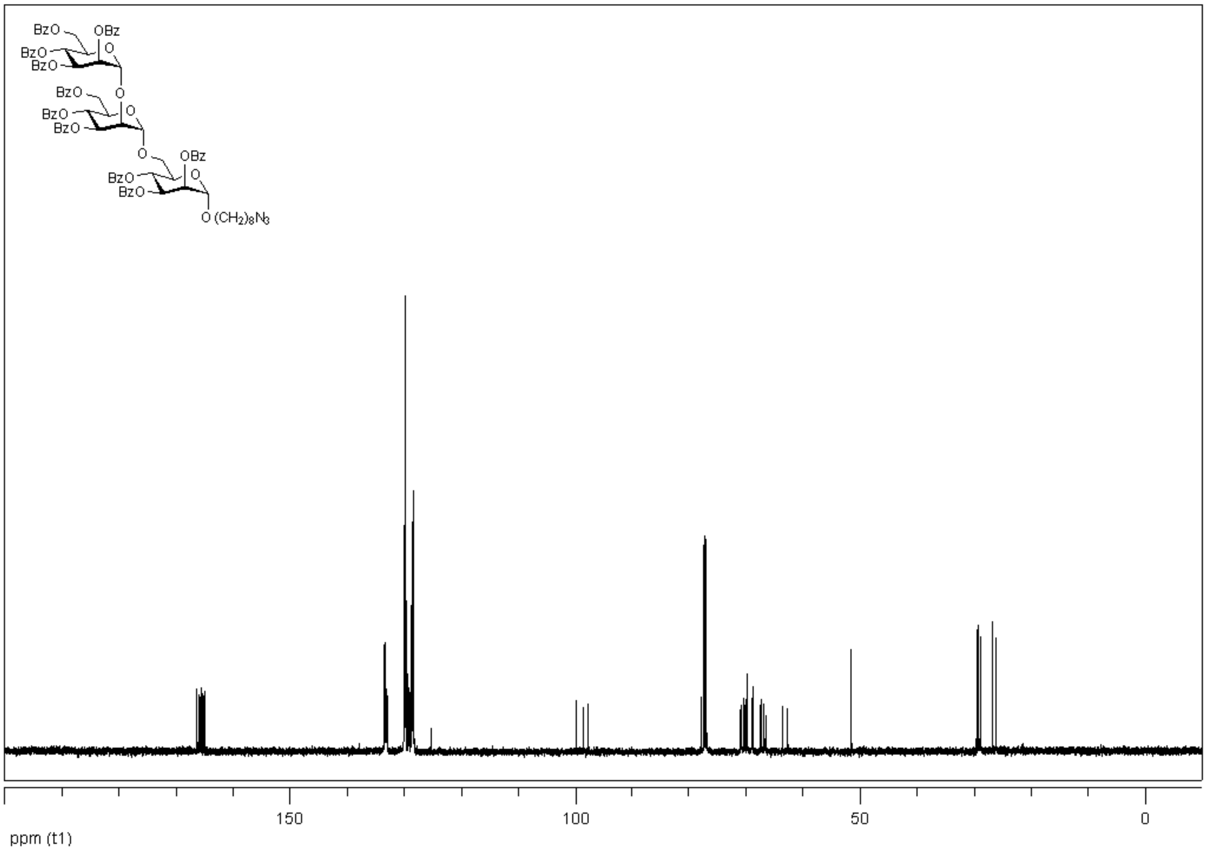

8-Azidooctyl (α-D-mannopyranosyl)-(1→2)-(α-D-mannopyranosyl)-(1→6)-α-D-mannopyranoside (3)

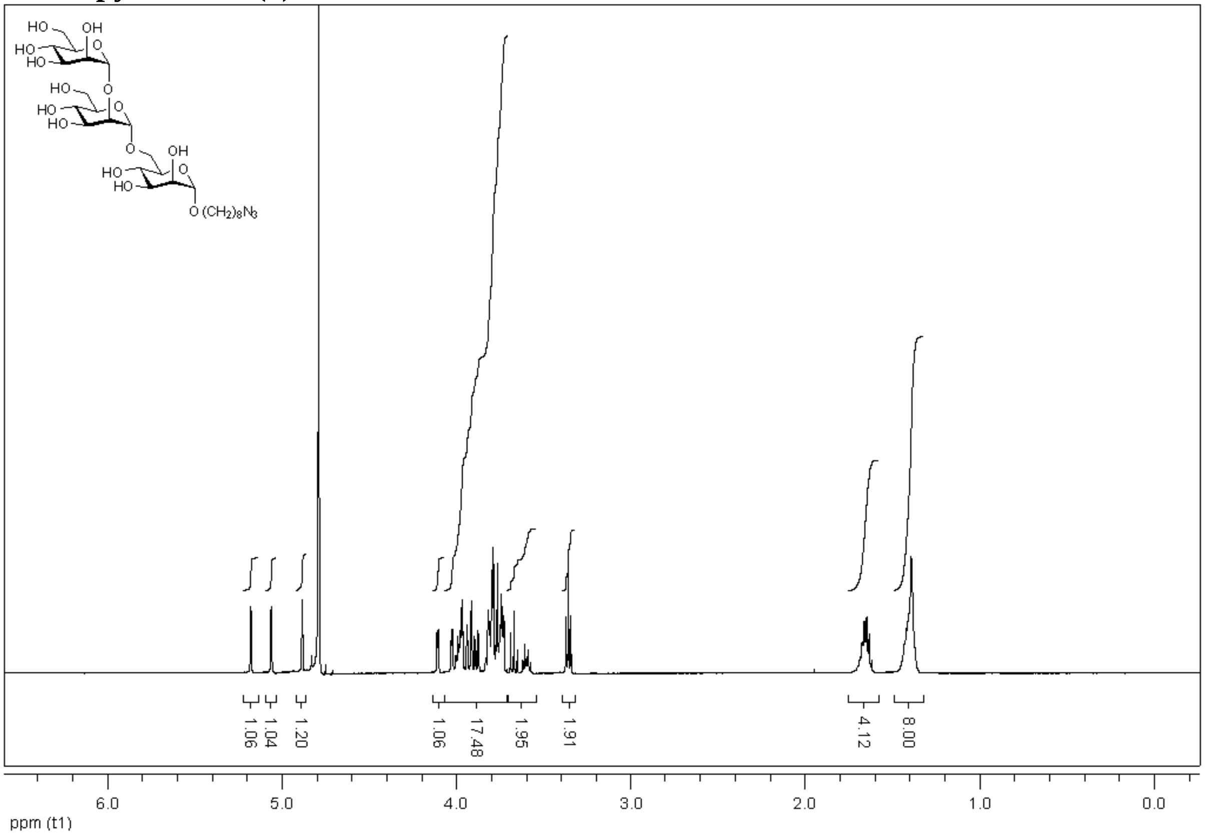

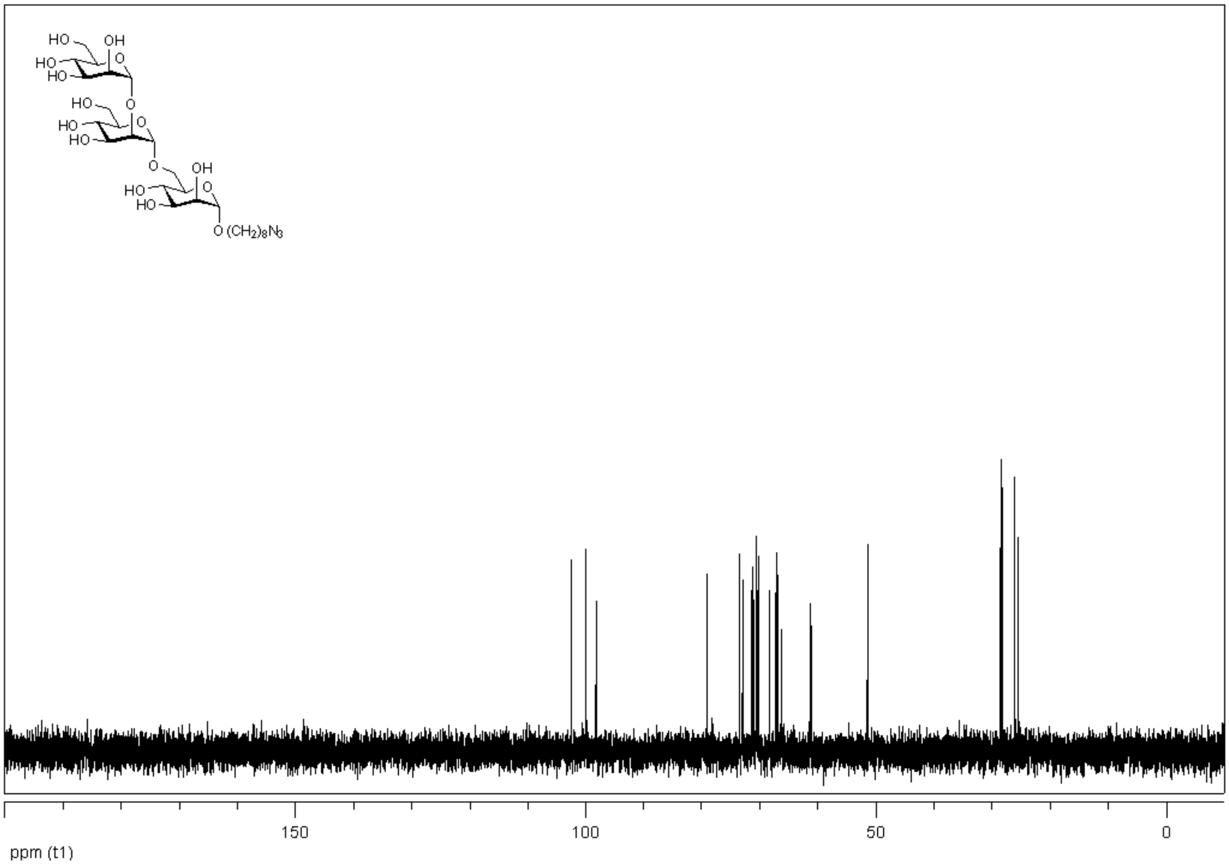

Azidooctyl 2,6-di-*O*-(2,3,4,6-tetra-*O*-benzoyl-α-D-mannopyranosyl)-3,4-di-*O*-(2,3-dimethoxybutane-2,3-diyl)-α-D-mannopyranoside (29)

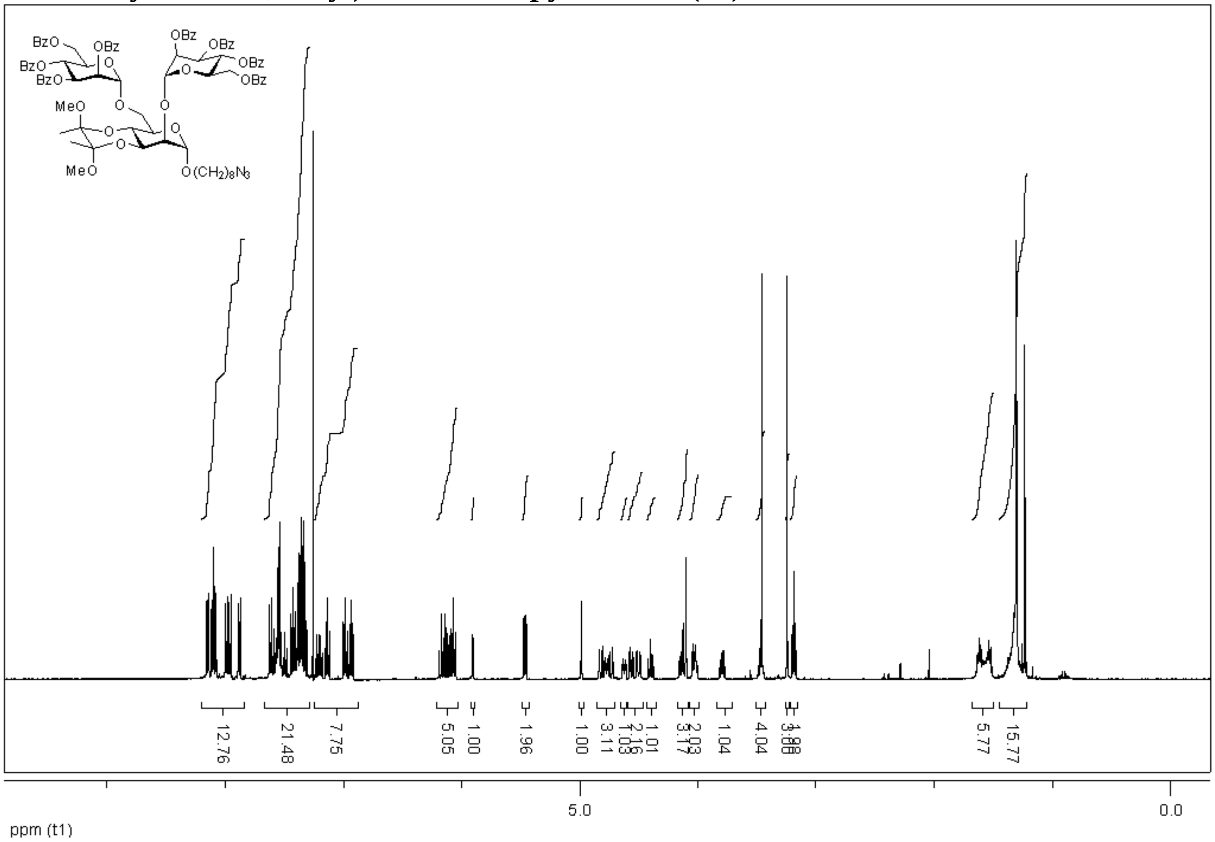

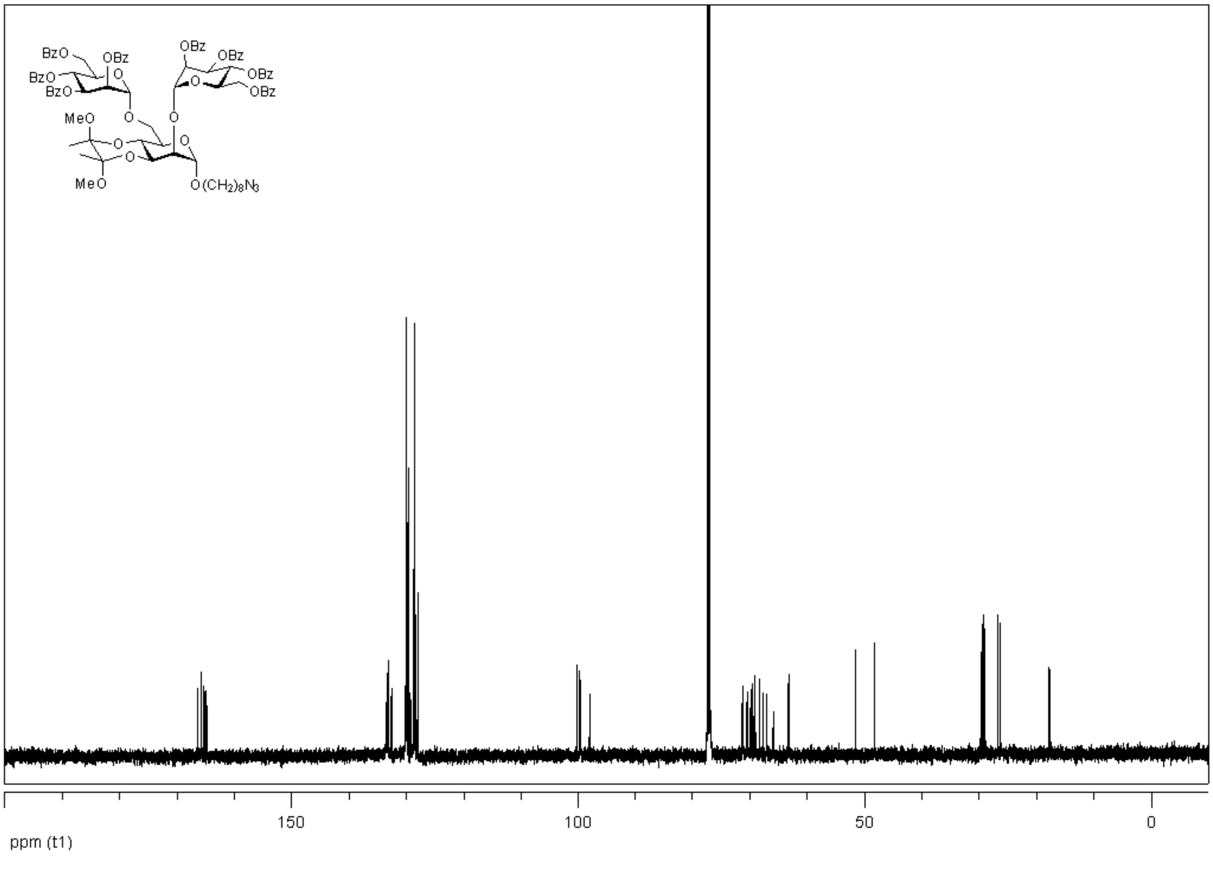

Azidooctyl 2,6-di-*O*-α-D-mannopyranosyl-α-D-mannopyranoside (4)

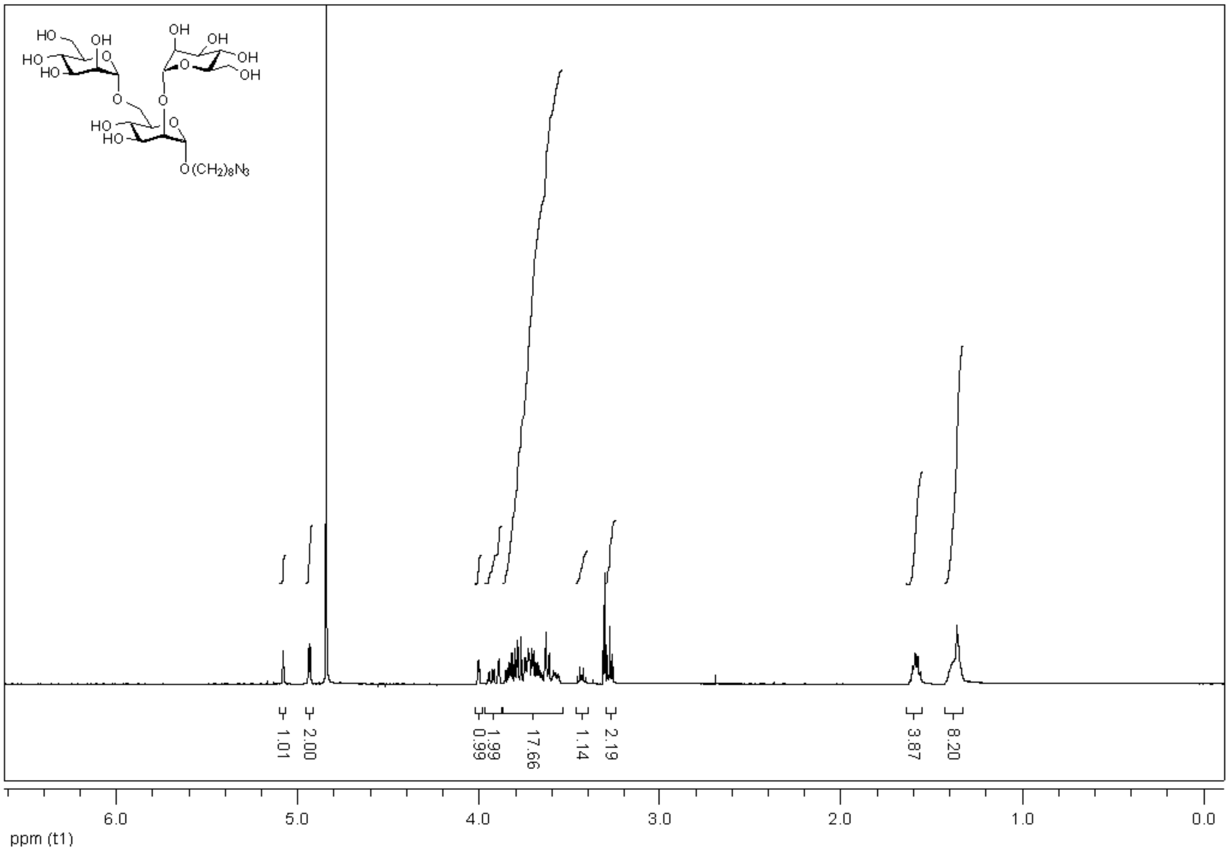

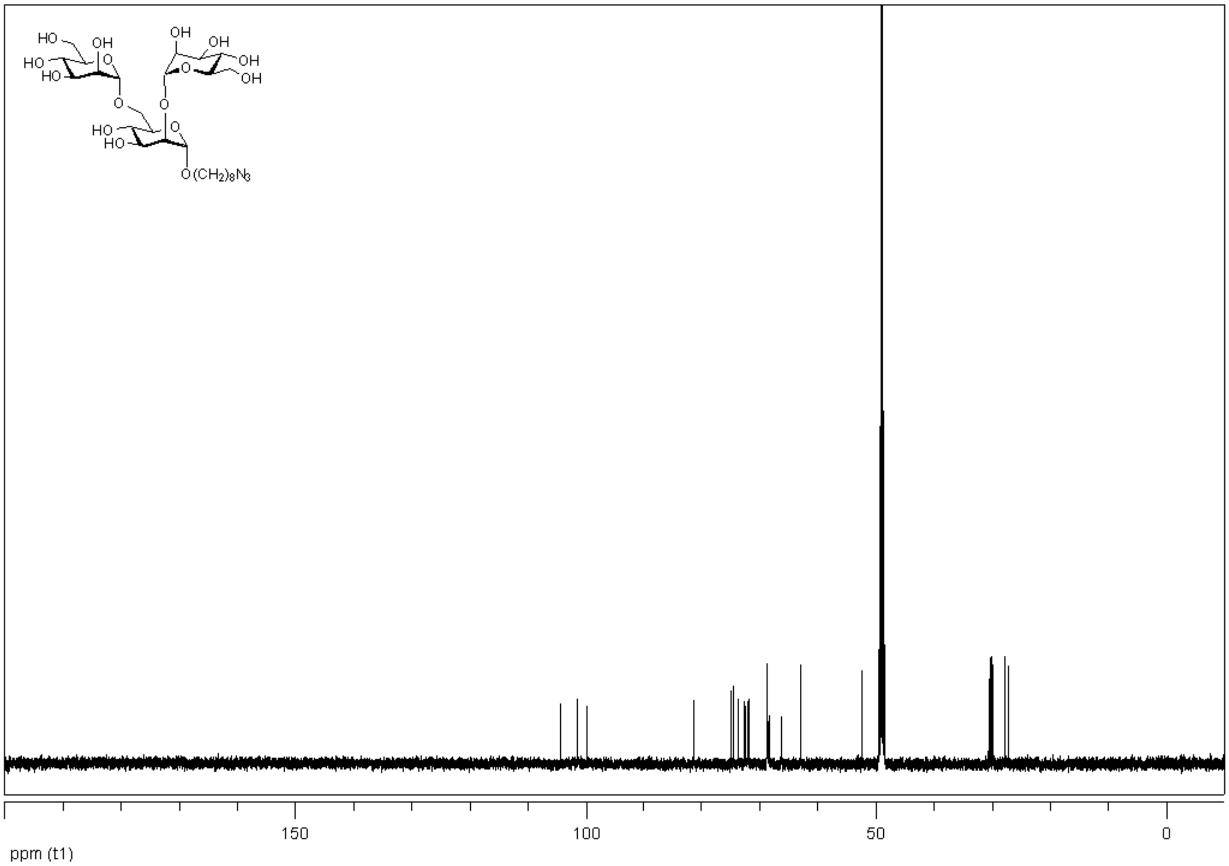

4-[(7-Nitrobenzofurazan-4-yl)aminomethyl]-triazol-1-yloctyl (α-D-mannopyranosyl)-(1→6)-α-D-mannopyranoside (5)

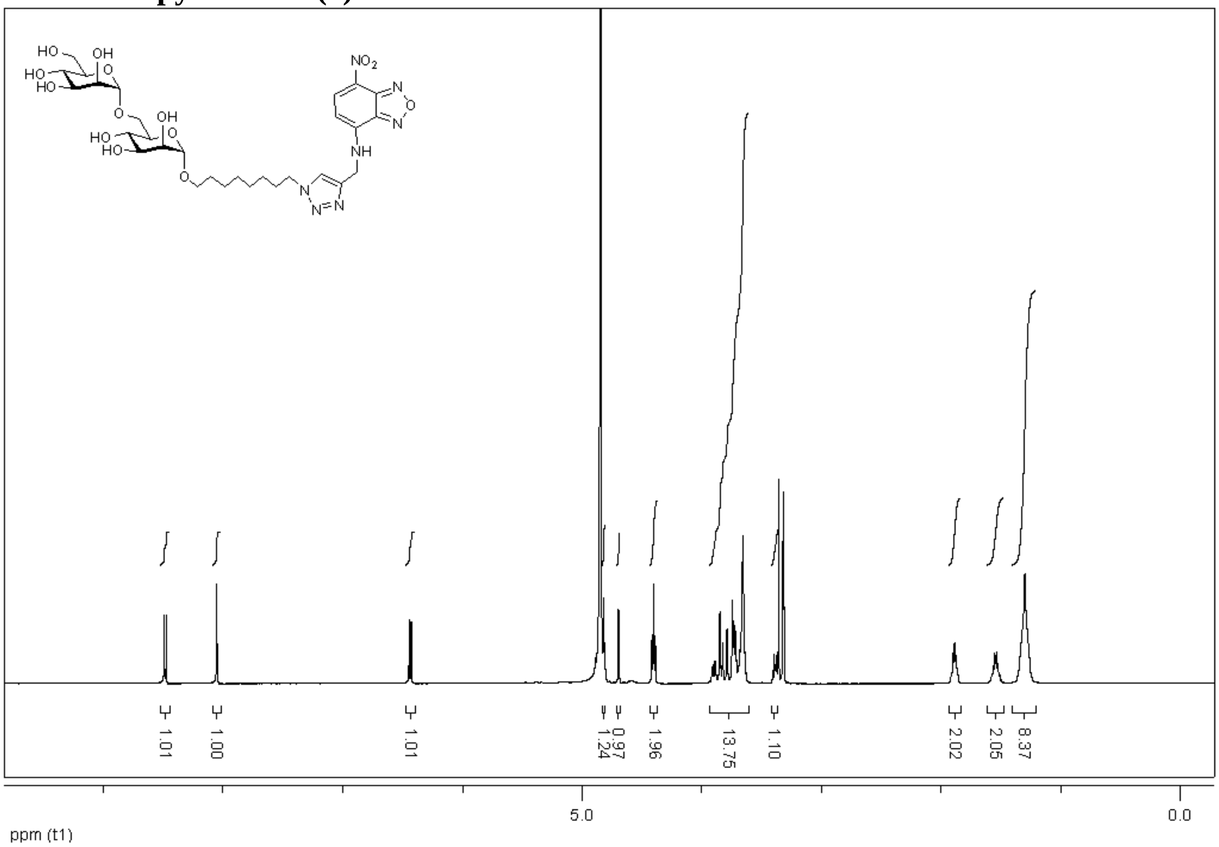

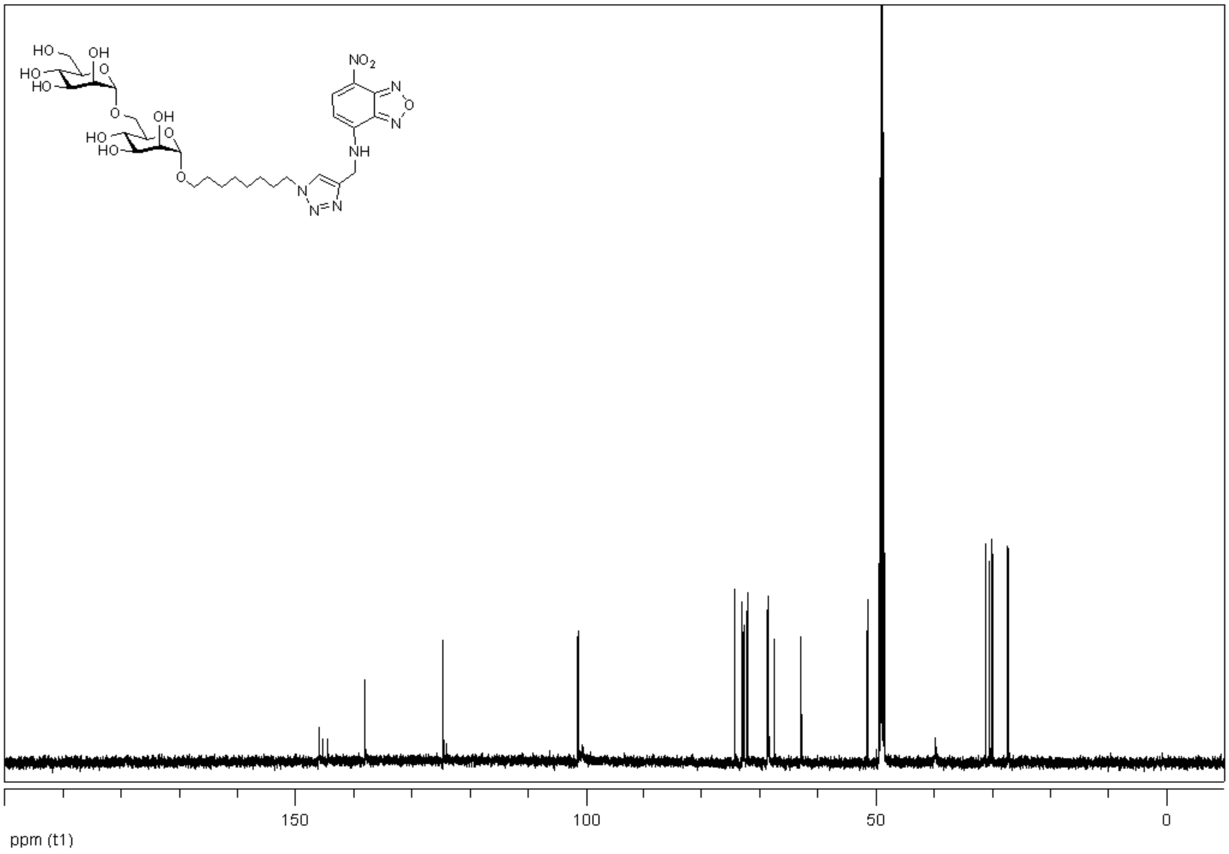

4-[(7-Nitrobenzofurazan-4-yl)aminomethyl]-triazol-1-yloctyl (α-D-mannopyranosyl)-(1→6)-(α-D-mannopyranosyl)-(1→6)-α-D-mannopyranoside (6)

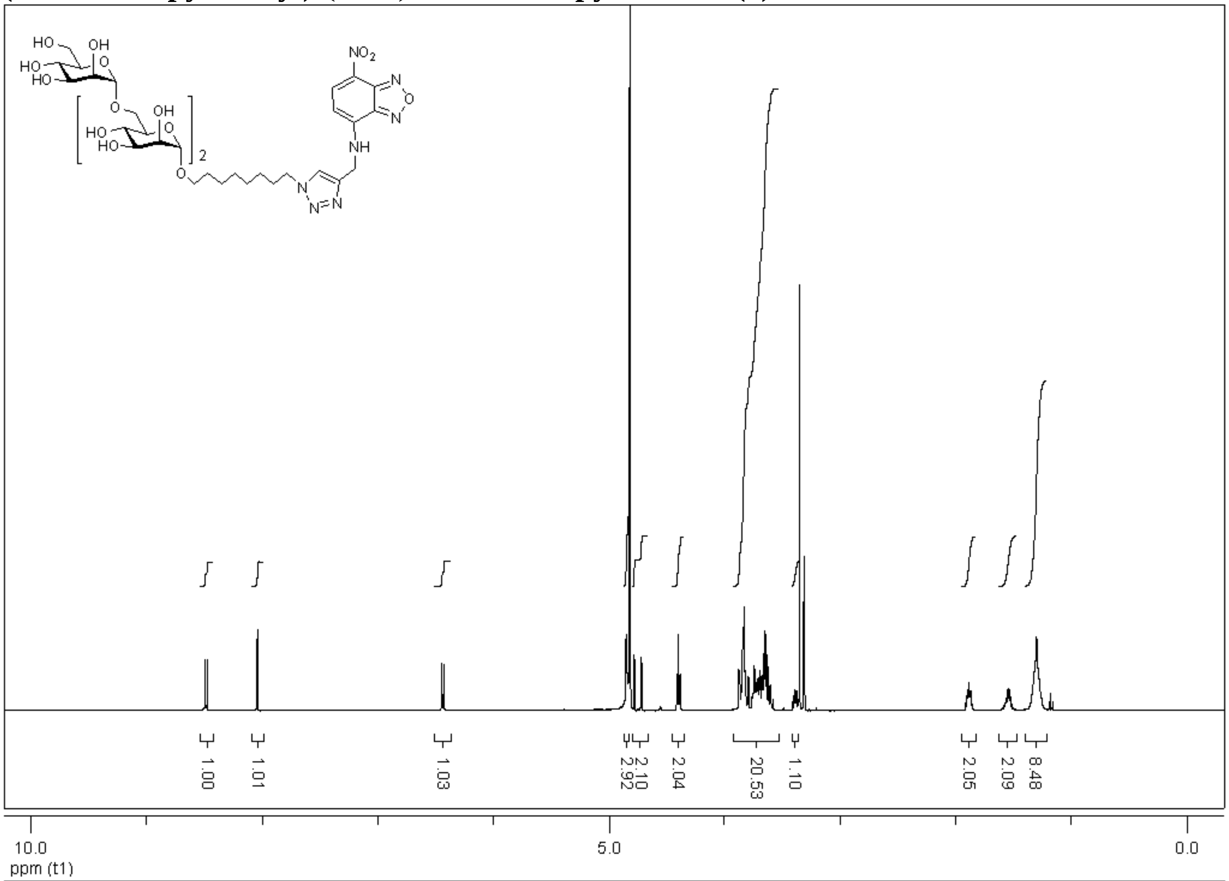

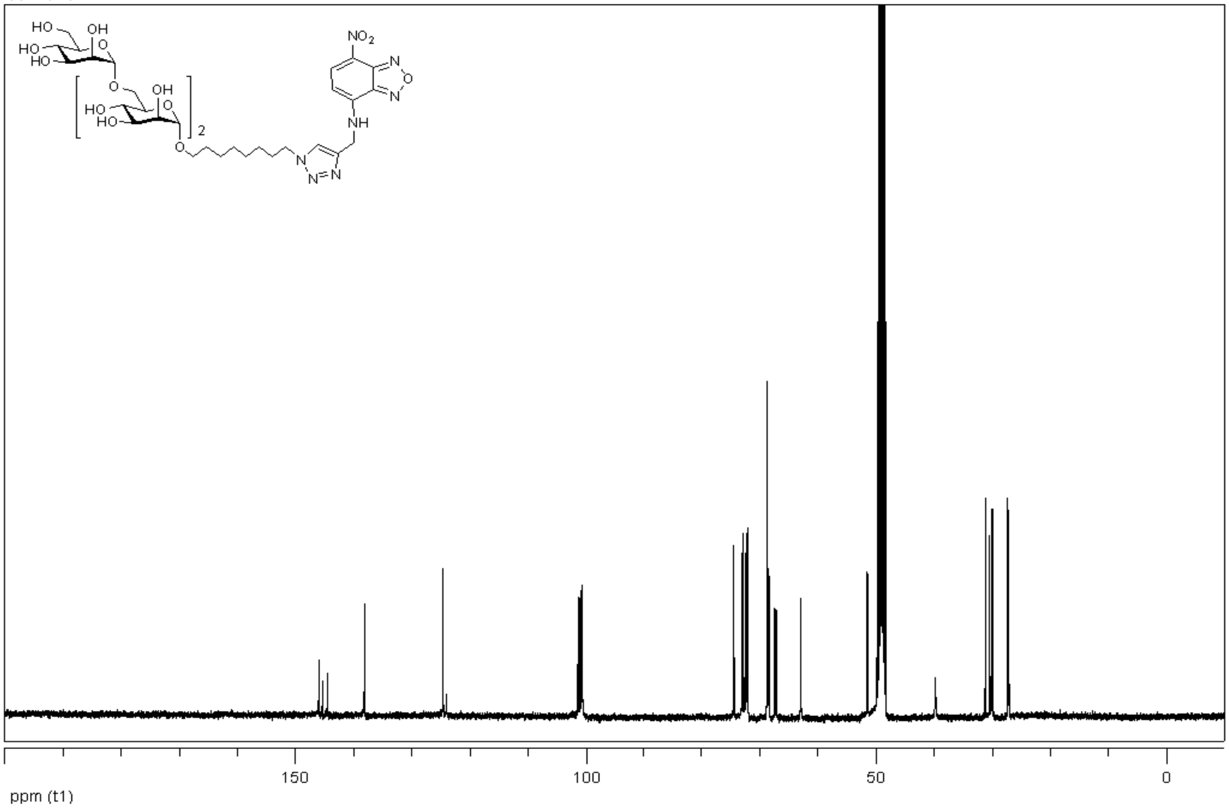

4-[(7-Nitrobenzofurazan-4-yl)aminomethyl]-triazol-1-yloctyl (α-D-mannopyranosyl)-(1→2)-(α-D-mannopyranosyl)-(1→6)-α-D-mannopyranoside (7)

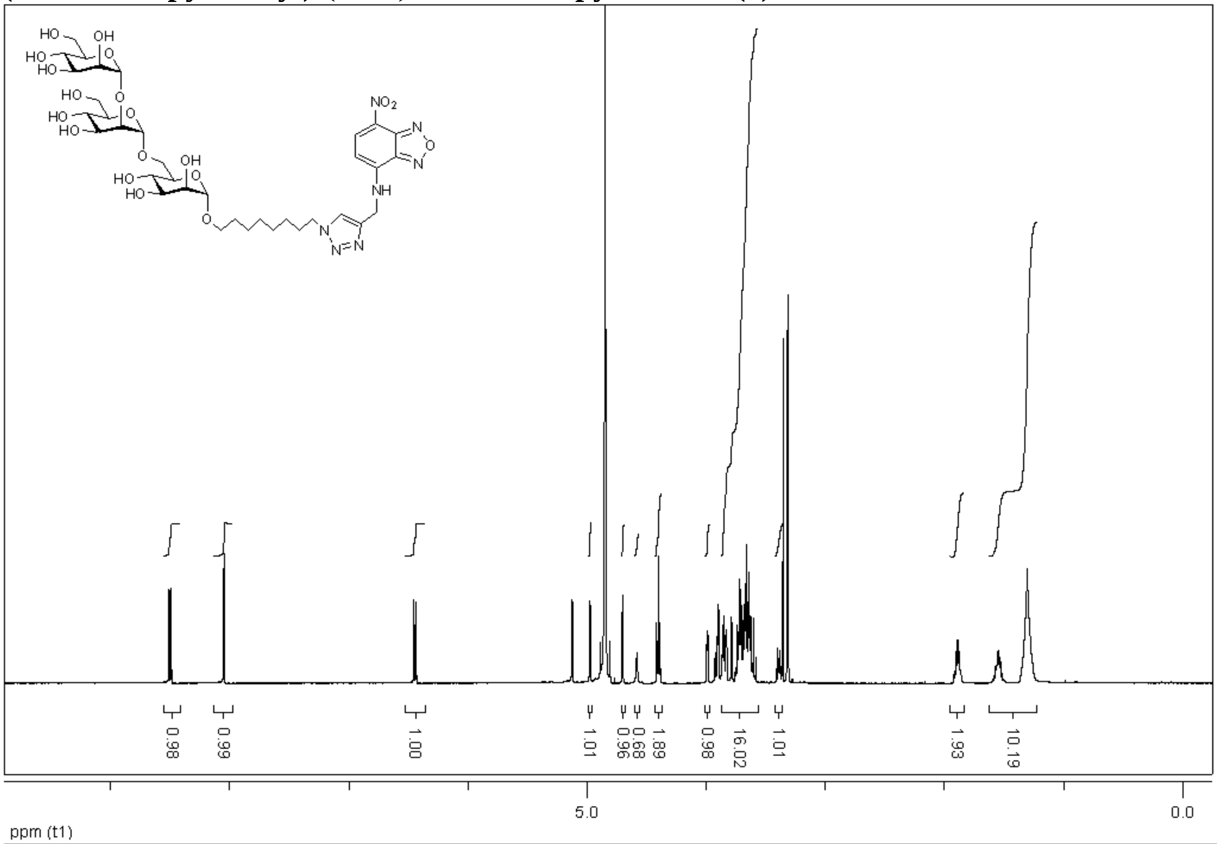

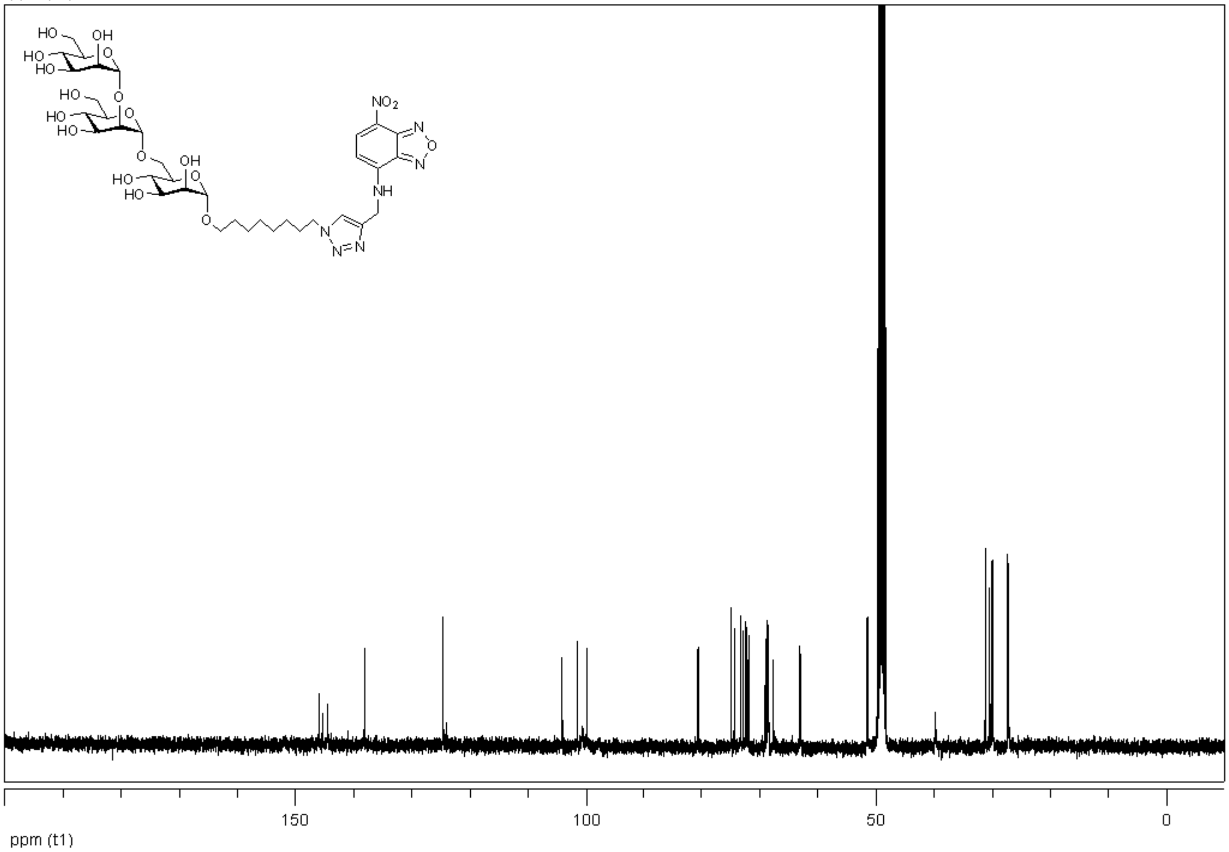

8-(2-Ethoxycylobutene-3,4-dione-1-ylamino)octyl (α-D-mannopyranosyl)-(1→6)-(α-D-mannopyranosyl)-(1→6)-α-D-mannopyranoside (8)

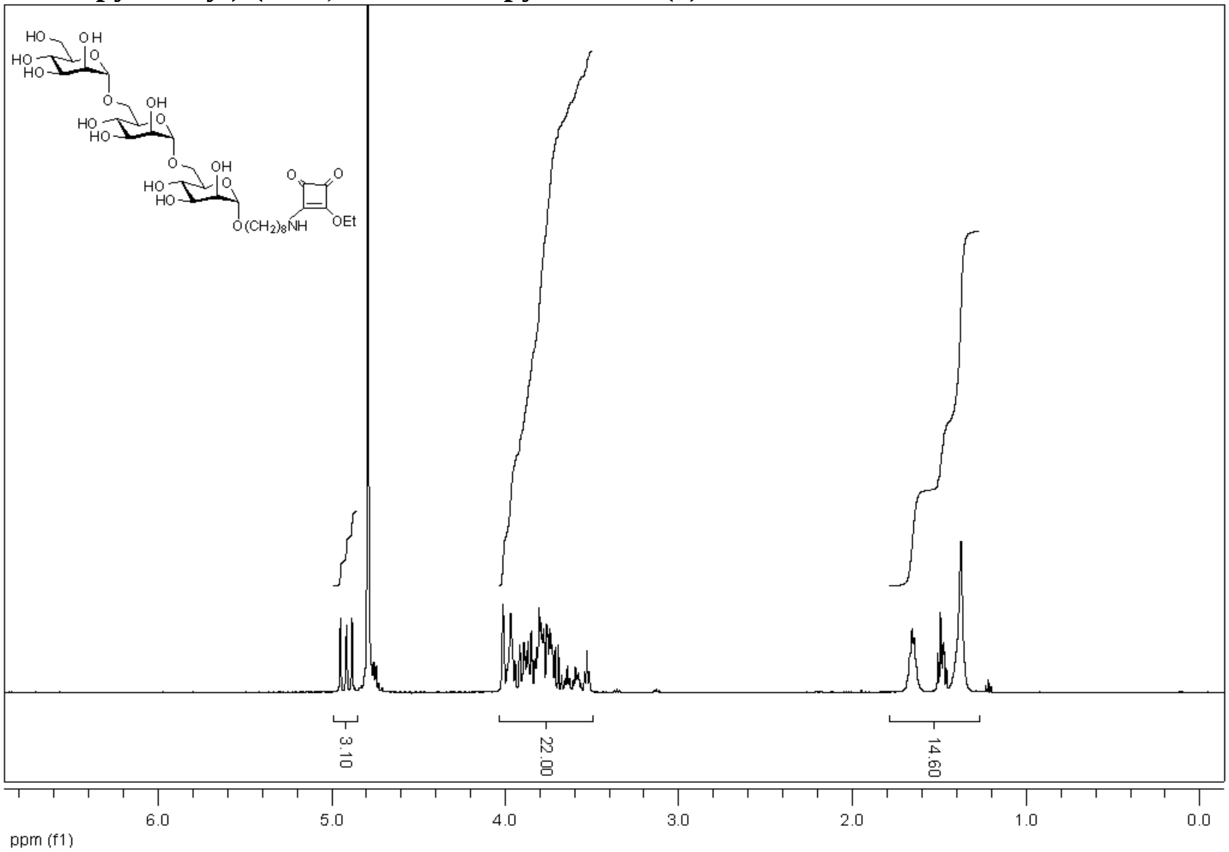

8-(2-Ethoxycylobutene-3,4-dione-1-ylamino)octyl (α-D-mannopyranosyl)-(1→2)-(α-D-mannopyranosyl)-(1→6)-α-D-mannopyranoside (9)

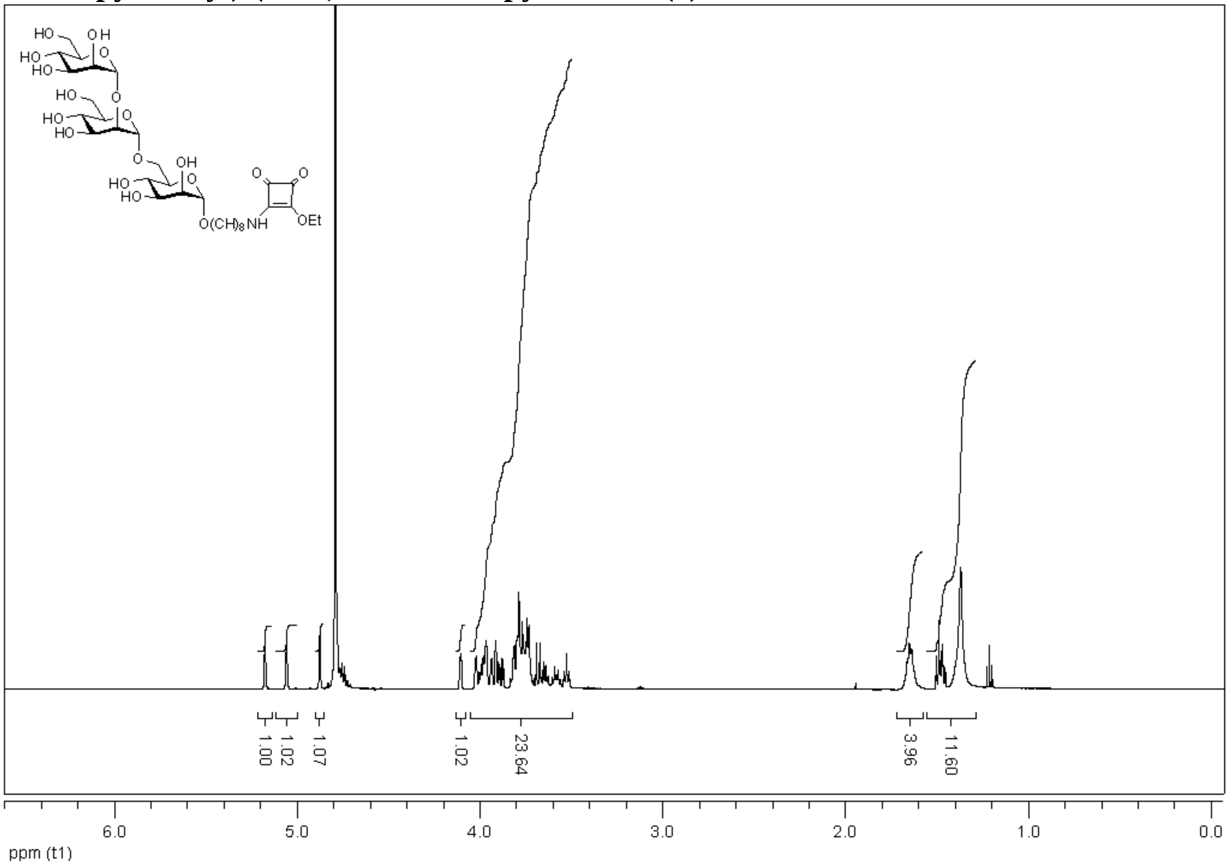

8-(2-Ethoxycylobutene-3,4-dione-1-ylamino)octyl 2,6-di-*O*-α-D-mannopyranosyl-α-D-mannopyranoside (10)

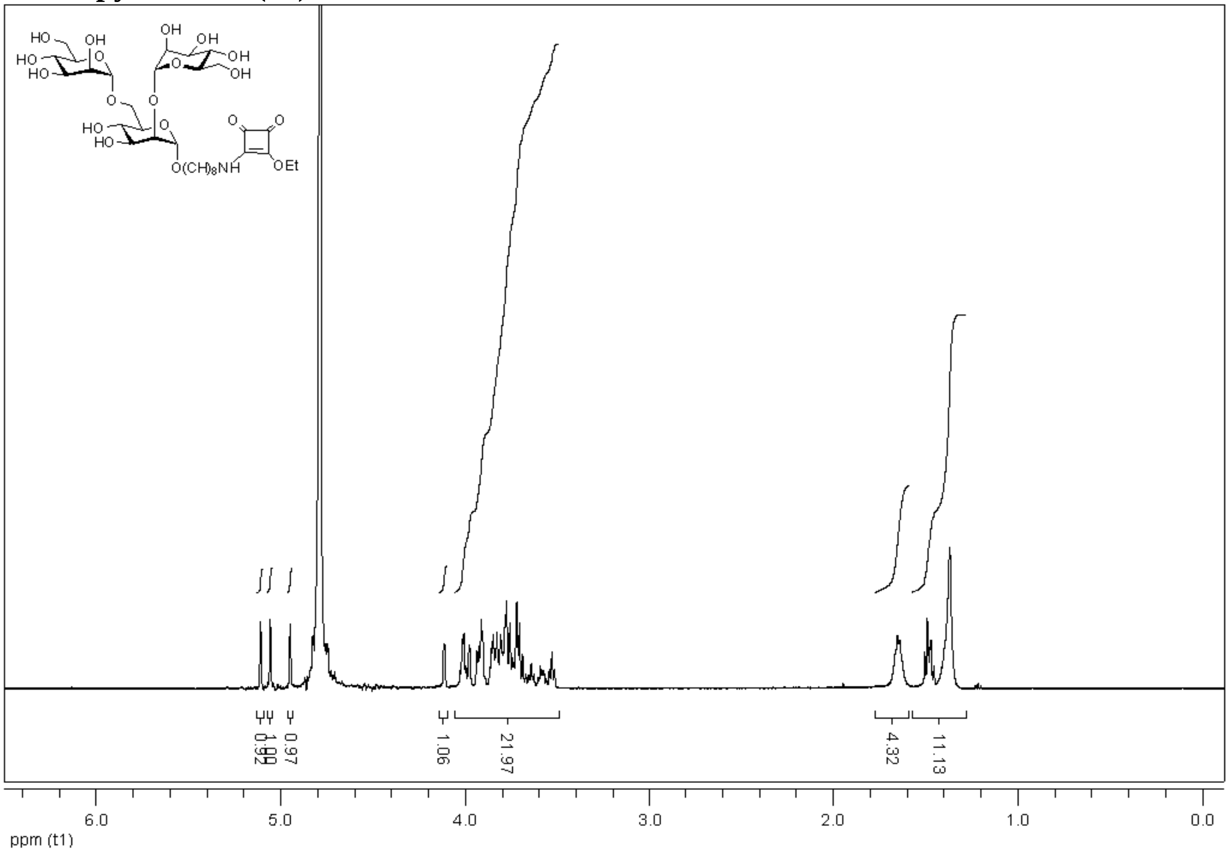

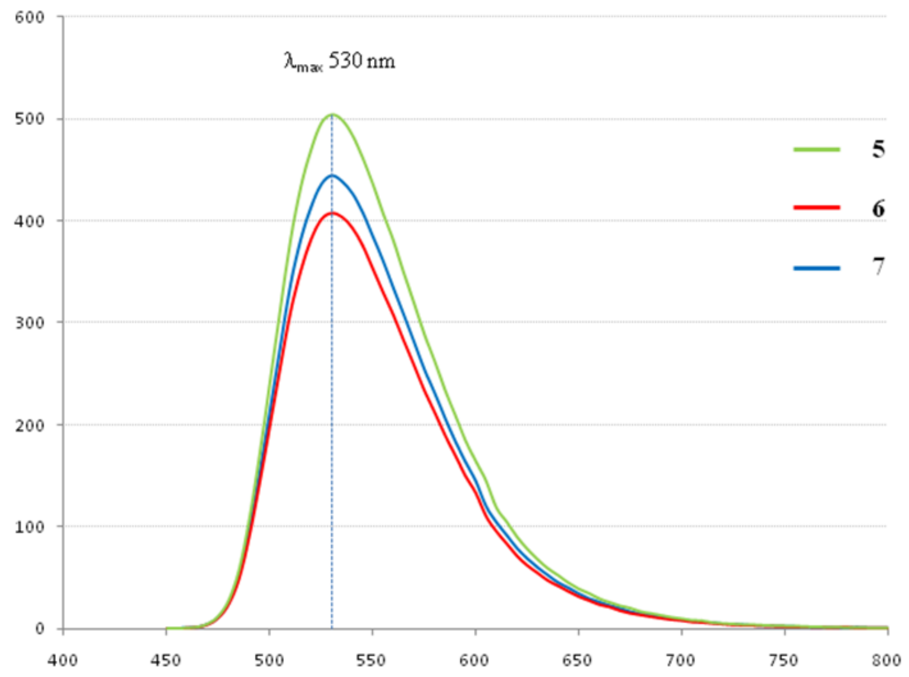

Figure 1: Fluorescence emission spectra of the NBD-labelled neoglycoconjugates **5**–**7**.

λ_{ex} = 400 nm at 0.1 mg ml^{-1} in water.

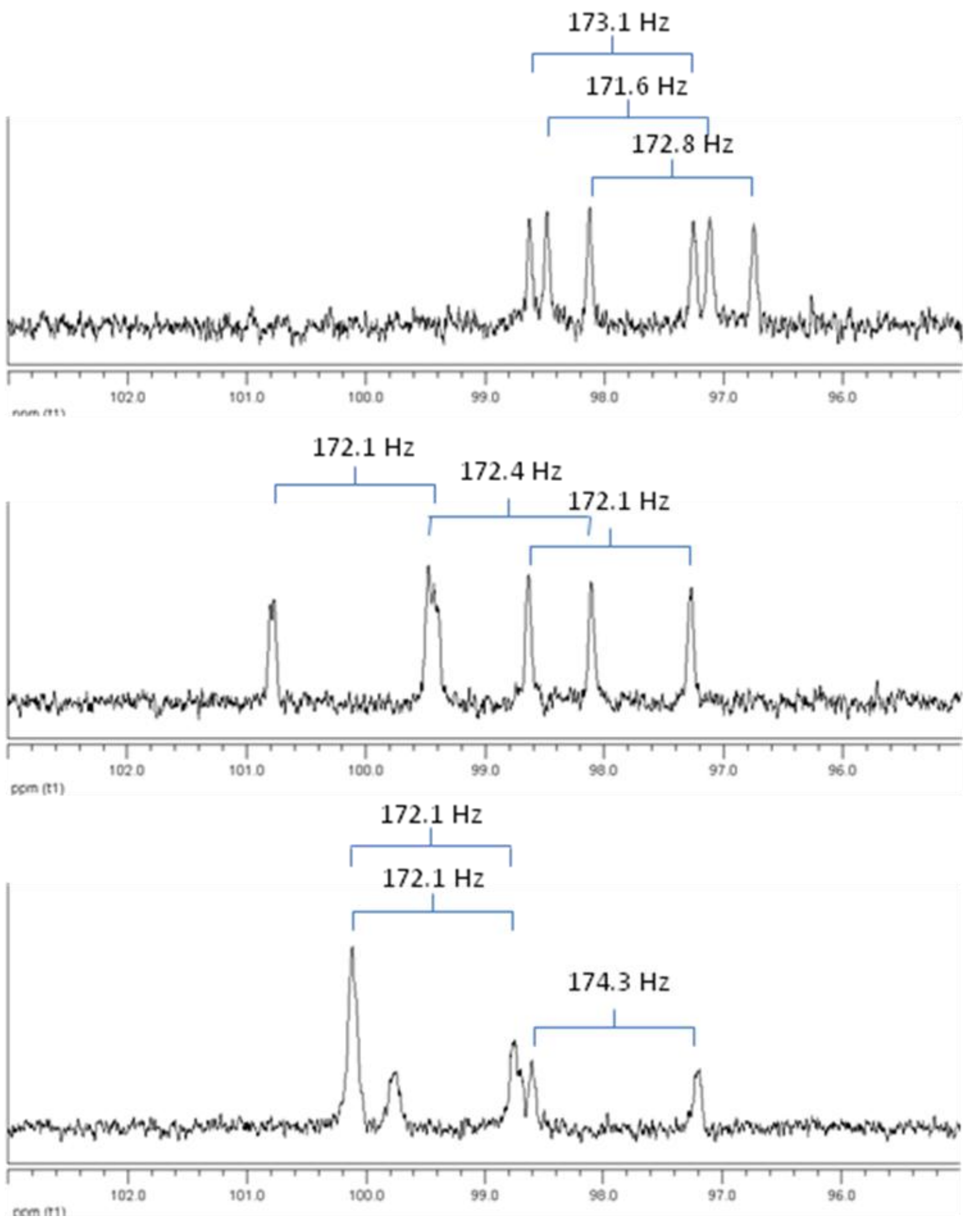

Figure 2: 125 MHz ^{1}H coupled ^{13}C spectra of trisaccharides **25**, **28** and **29**. $^{1}J_{C,H}$ values of anomeric carbons are indicated.
